# Climate and tree seed production predict the abundance of the European Lyme disease vector over a 15-year period

**DOI:** 10.1186/s13071-020-04291-z

**Published:** 2020-08-10

**Authors:** Cindy Bregnard, Olivier Rais, Maarten Jeroen Voordouw

**Affiliations:** 1 Laboratory of Ecology and Evolution of Parasites, Institute of Biology, University of Neuchâtel, Neuchâtel, Switzerland; 2 Laboratory of Ecology and Epidemiology of Parasites, Institute of Biology, University of Neuchâtel, Neuchâtel, Switzerland; 3 Department of Veterinary Microbiology, Western College of Veterinary Medicine, University of Saskatchewan, Saskatoon, Canada

**Keywords:** Beech tree, Climate change, *Fagus sylvatica*, *Ixodes ricinus*, Lyme disease, Mast years, Tick population ecology, Tick-borne disease

## Abstract

**Background:**

To predict the risk of tick-borne disease, it is critical to understand the ecological factors that determine the abundance of ticks. In Europe, the sheep tick (*Ixodes ricinus*) transmits a number of important diseases including Lyme borreliosis. The aim of this long-term study was to determine the abiotic and biotic factors driving the annual abundance of *I. ricinus* at a location in Switzerland where Lyme borreliosis is endemic.

**Methods:**

Over a 15-year period (2004 to 2018), we monitored the abundance of *I. ricinus* ticks on a monthly basis at three different elevations on Chaumont Mountain in Neuchâtel, Switzerland. We collected climate variables in the field and from nearby weather stations. We obtained data on beech tree seed production from the literature, as the abundance of *Ixodes* nymphs can increase dramatically two years after a masting event. We used AIC-based model selection to determine which ecological variables drive annual variation in tick density.

**Results:**

We found that elevation site, year, seed production by beech trees two years prior, and mean annual relative humidity together explained 73.2% of the variation in our annual estimates of nymph density. According to the parameter estimates of our models, (i) the annual density of nymphs almost doubled over the 15-year study period, (ii) changing the beech tree seed production index from very poor mast (1) to full mast (5) increased the abundance of nymphs by 86.2% two years later, and (iii) increasing the field-collected mean annual relative humidity from 50.0 to 75.0% decreased the abundance of nymphs by 46.4% in the same year. Climate variables collected in the field were better predictors of tick abundance than those from nearby weather stations indicating the importance of the microhabitat.

**Conclusions:**

From a public health perspective, the increase in nymph abundance is likely to have increased the risk of tick-borne disease in this region of Switzerland. Public health officials in Europe should be aware that seed production by deciduous trees is a critical driver of the abundance of *I. ricinus*, and hence the risk of tick-borne disease. 
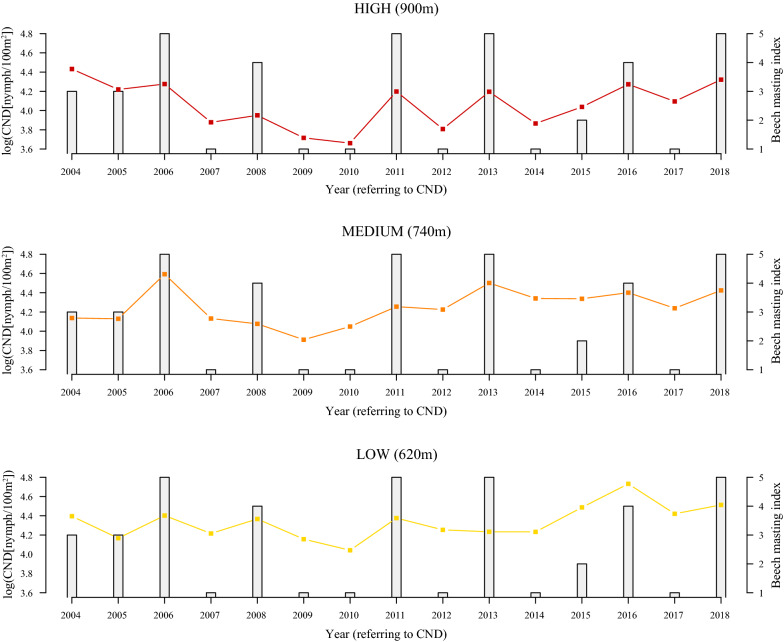

## Background

Climate change has resulted in dramatic changes in the distribution and abundance of medically important arthropod vectors, such as mosquitoes, ticks and biting flies [1, 2]. These range expansions are expected to increase the incidence of vector-borne diseases that are transmitted by these arthropod vectors with serious consequences for human health [3]. For example, the range expansion of *Ixodes* ticks into northern latitudes has resulted in a dramatic increase in the incidence of Lyme borrelosis and other tick-borne diseases in Canada and Scandinavia [4–9]. In contrast, whether climate change is affecting the abundance of *Ixodes* ticks in areas where they are endemic is not clear [10–17].

Climate change will influence the population ecology of *Ixodes* ticks *via* its effects on both abiotic factors (e.g. climate variables) and biotic factors (e.g. vegetation or host community). *Ixodes* ticks spend ~ 98% of their time off the host and have to cope with seasonal changes in temperature and precipitation. Their life history traits (development, survival and reproduction) are highly sensitive to climate variables that are changing due to global warming [18]. For example, tick development rates and survival increase with temperature and relative humidity, respectively [19–22]. However, we currently do not have a good understanding of how climate change will influence the abundance of *Ixodes* ticks in endemic areas [2, 20].

With respect to biotic factors, climate change is expected to drive changes in plant and animal communities that will feed back on the population ecology of *Ixodes* ticks. For example, climate influences seed production in beech and oak trees [23–26], which affects the population dynamics of animal species that use these seeds as a food resource, such as rodents and birds [27, 28]. In turn, fluctuations in rodent abundance can drive variation in the abundance of *Ixodes* ticks because ticks feed on rodents [10, 27, 29–31]. Previous work in North America has shown that the density of *Ixodes* ticks and the risk of Lyme disease depend on the abundance of rodents in the previous year and on the abundance of acorns two years previously [10, 31–33]. In contrast, in Europe there is no direct evidence demonstrating that tree seed production is driving the population dynamics of *Ixodes* ticks [34–36].

In Europe, the sheep tick (*Ixodes ricinus*) transmits a number of important diseases including Lyme borreliosis [37]. *Ixodes ricinus* is a generalist tick species that feeds on a variety of vertebrate hosts, including mammals, birds and reptiles [38]. *Ixodes* ticks have three active stages: larva, nymph and adult; each stage requires a single blood meal to develop to the next stage (female ticks take a blood meal to produce eggs). Blood meals often occur in different years, and the total life-cycle can range from 3 to 5 years. Thus, *Ixodes* tick population ecology is characterized by cohorts of larvae, nymphs and adults that were born in different years, but that search for hosts at the same time of year. In addition, *Ixodes* ticks have highly seasonal activity patterns; they search for hosts during the spring, summer, and fall and enter diapause during the winter. As most tick sampling methods only capture questing ticks (i.e. ticks searching for a blood meal), within-year field studies inevitably capture the dramatic seasonal changes in the abundance of questing ticks.

The aim of this study was to better understand how climate and altitude influence the ecology of *I. ricinus* ticks. We used a long-term study to test whether the density of *I. ricinus* has increased along an altitudinal gradient in Switzerland, and whether any such increase in tick abundance was correlated with climate change. Specifically, we used data from a 15-year study that monitored the monthly abundance of *I. ricinus* nymphs and adult ticks at four different elevations on Chaumont Mountain, in the canton of Neuchâtel, Switzerland. We collected climate variables that are known to be important for tick ecology (e.g. temperature, relative humidity, saturation deficit and precipitation) and we obtained data on seed production by beech trees from the literature. We expected tick abundance to be higher at the lower elevations compared to the higher elevations. We predicted tick abundance to increase over time at all elevations, and that this increase would be more pronounced at the higher elevations compared to the lower elevations. Finally, we predicted that the intensity of seed production by beech trees would drive the abundance of nymphs two years later and the abundance of adult ticks three years later.

## Methods

### Study location

The study was conducted on the south-facing slope of Chaumont Mountain, which is part of the Jura mountains, and is located in the canton of Neuchâtel, in western Switzerland. The forest on Chaumont Mountain is mainly composed of European beech (*Fagus sylvatica*; 28.6%), Norway spruce (*Picea abies*; 28.5%), European silver fir (*Abies alba*; 20.4%), sycamore maple (*Acer pseudoplatanus*; 5.9%), European ash (*Fraxinus excelsior*; 3.7%), Scots pine (*Pinus sylvestris*; 2.3%), sessile oak (*Quercus petraea*; 2.3%), willows (*Salix* spp.; 2.1%), common whitebeam (*Sorbus aria*; 1.6%), and European hornbeam (*Carpinus betulus*; 1.0%). There is logging activity in the area, and there are also hiking trails and recreation areas for the public.

Four tick sampling sites, referred to as low, medium, high and top, were established at elevations of 620, 740, 900 and 1073 m above sea level (ASL), respectively. The four sites have been previously described [39, 40]. During the study, the canton of Neuchâtel approved the construction of a network of mountain bike trails and an adventure park, which were located at a distance of 25 m from the top site. The construction of the network of mountain bike trails occurred from 2006 to 2010, and maintenance work on the trail is done every year. The construction of the adventure park started in 2011 and each year it is heavily used by the public from April to October. We believe that the construction of these recreation facilities reduced the quality of the forest habitat at the top elevation and compromised its integrity as a long-term field site.

### Tick collection

Questing *I. ricinus* nymphal and adult ticks were collected on a monthly basis over a period of 15 years from January 2004 to December 2018 at each of the four elevations. The sampling protocol has been previously described [41]. Briefly, a 1-m^2^ cotton flag was dragged across low vegetation over a distance of 100 m (low elevation) or 120 m (other elevations). The flag was inspected every 20 m and nymphs and adults were counted separately. The same person (Olivier Rais) conducted all of the 3427 drags (15 years × 12 months × 5 or 6 drags × 4 elevations = 4140 drags). Drags were not performed when there was snow on the ground (713 drags).

### Field-collected climate data

Temperature (T; units are °C) and relative humidity (RH; units are %) were recorded at 60 cm above ground at one moment in time on the day of tick collection (usually between 10:00 am and 2:00 pm) at each sampling site using a thermohygrometer (Model 615, Testo SA, Lonay, Switzerland). Thus, for each combination of elevation and year, we had a total of 12 field-collected measurements of temperature and relative humidity. The saturation deficit (SD) is a measure of the drying power of the atmosphere (units are mm of mercury) and is calculated using temperature (T) and relative humidity (RH) as follows: SD = (1 − RH/100) × 4.9463 × e^0.0621T^ [42, 43]. We confirmed that our field-collected climate data were accurate by comparing them to the Climap-net data (Additional file 1: Sections 1 and 2).

### Climap-net data

In addition to the field-collected data, we obtained climate data from the Climap-net database of the Swiss meteorological center (MeteoSuisse). Two weather stations close to our four elevation sites are located in Neuchâtel at 485 m ASL and in Chaumont at 1136 m ASL. These weather stations sample at 200 cm above ground the temperature and relative humidity every hour, and the total precipitation each day. We used the daily mean temperature (average of the 24-hourly measurements), the daily mean relative humidity, and the daily total precipitation. Thus, for each year, we had a total of 365 Climap-net measurements of the daily mean temperature, the daily mean relative humidity, and the daily total precipitation. The saturation deficit was calculated as previously described. For each of the four elevation sites, we calculated site-specific climate variables by interpolating the data between the two weather stations (Additional file 1: Section 3).

### Tree masting data

Mast refers to the fruit of forest trees, such as the acorns of oak trees and the beech nuts of beech trees. Data on masting (or seed production) were obtained for Neuchâtel from the MASTREE database for two important tree species: European beech (*Fagus sylvatica*) and Norway spruce (*Picea abies*) [44]. These two species accounted for 57.1% of the trees at our study location. In this database, the mast intensity is classified into five classes: 1, 2, 3, 4, and 5, which refer to very poor mast, poor mast, moderate mast, good mast, and full mast, respectively.

### Statistical methods

Statistical analyses were restricted to the three lower elevation sites (low, medium and high). The top elevation was excluded because tick abundance at this site declined dramatically following the construction of the network of mountain bike trails and the adventure park. In Additional file 1: Sections 4 and 5, the data are analyzed with all four elevation sites together.

#### Annual cumulative tick density

The cumulative nymph density (CND) is a measure of the total annual abundance of questing nymphs per 100 m^2^ and was estimated by integrating the area under the curve (AUC) of the 12 monthly questing nymph densities for each year [43, 45]. We used this AUC approach because it is less likely to be biased by missing data compared to calculating a simple average for each year. We assume that the CND represents a small unknown fraction of the nymphs that were actually present in the area. No dragging was performed on days when there was snow on the ground (hereafter referred to as snow days). Over the study period (15 years × 12 months = 180 days), a total of 34 snow days occurred, and these were coded as missing data. In summary, tick abundance data from 2581 individual drags were collapsed into 45 estimates of CND (15 years × 3 elevations = 45 annual estimates of abundance). The same approach was used to calculate the cumulative adult tick density (CAD).

#### Annual mean climate variables

To investigate the relationship between climate and our annual estimates of tick abundance, we collapsed our monthly or daily weather data into a single annual value. For the field-collected data, the annual means were calculated over the 12 monthly measurements (i.e. a single measurement for each month). For the Climap-net data, the annual means were calculated over 365 daily means (i.e. a total of 365 days × 24 measurements/day = 8760 hourly measurements). Thus, the Climap-net annual means were based on 730 times more data than the field-collected annual means. However, an important advantage of the field-collected data was that they were specific for each of the four elevation sites. In contrast, the Climap-net data came from two weather stations that were located at some distance from the four elevation sites. To facilitate comparison between the slopes of the climate variables, we standardized the climate variables to z-scores (mean of 0, standard deviation of 1).

#### Annual tree masting variables

The model by Ostfeld et al. [10] predicts a 2-year time lag between masting events and the CND and a 3-year time lag between masting events and the CAD. To validate the model by Ostfeld et al. [10], we compared five different time lags where the CND is predicted by mast events that occurred 0, 1, 2, 3 and 4 years into the past (Additional file 1: Section 9). This analysis confirmed that the 2-year time lag is an order of magnitude better than all of the other time lags considered. The 2-year time lag has a partial *r*^2^ value of 26.3% and a *P*-value of 0.0002. In contrast, the partial *r*^2^ values of the other four time lags (0, 1, 3 and 4 years in the past) are much lower (range: 0.0–5.1%) and none of the *P*-values are statistically significant. This preliminary analysis validates our decision to model the CND and CAD as a function of the tree mast scores (of European beech and Norway spruce) two years previously (year y-2) and three years previously (year y-3), respectively. For example, tree mast scores from the year 2001 predicted the CND in year 2003 (2 years later) and the CAD in year 2004 (three years later).

#### Analysis of the annual variation in nymph and adult tick abundance using linear models

Count data follow a Poisson distribution or a negative binomial distribution. However, our estimates of the CND (or CAD) are summary statistics (sums or integrals) that are based on the counts of ~ 60 drags (12 dates × 5 drags per date). According to the central limit theorem of statistics, summary statistics will follow a normal distribution even if the observations on which they are based are drawn from a non-normal distribution. We therefore assumed that the residuals of our CND values follow a normal distribution. The CND values were log_10_-transformed to further improve their fit to the normal distribution. The log_10_-transformed CND values (*n* = 45) were analyzed using linear models (LM) with normal errors. In section 11 of Additional file 1, we show that the analysis of the CND using generalized linear models with negative binomial errors (which are appropriate for overdispersed count data) gives the same results.

The explanatory variables included the elevation site (3 levels: low, medium, high), the covariate year (rescaled as 1, 2, 3, … 15), the covariate beech mast score (range: 1–5), the covariate spruce mast score (range: 1–5), mean annual temperature, mean annual RH, mean annual SD, and mean annual precipitation (all annual climate variables were transformed to z-scores). We used mean annual climate variables based on the field-collected data and on the Climap-net data to determine which climate variables were better for explaining variation in tick abundance. As time lags are known to be important in tick ecology, we modelled the CND as a function of the mean climate variable in the present year or the previous year. The same approach was used to model the CAD (*n* = 45).

#### Model selection approach

We used a model selection approach based on the Akaike information criterion (AIC) to find the most parsimonious model. Models were ranked according to their AIC values and the Akaike weights were calculated for each model. We used the Akaike weights to calculate the model-averaged parameter estimates and their 95% confidence intervals (CIs) (Additional file 1: Section 6). For the best model from the model selection table for the log_10_-transformed CND (and CAD), we confirmed that the residuals met the assumptions of normality and equal variances (Additional file 1: Section 7). This result shows that we were justified in using linear models to analyze the CND and CAD.

We used R version 3.6.1 for all statistical analyses [46]. We used the *auc()* function (integrates the area under the curve) in the *flux* package to estimate the CND and the CAD for each year. We used the *lm()* function in the basic package to model the log_10_-transformed CND as a linear model of the explanatory variables. We used the *mod.sel()* function and the *model.av()* function in the *MuMIn* package. The *mod.sel()* function creates an automated model selection table using previously generated models (linear models with normal errors in our case), and the *mod.av()* function calculates model-averaged parameter estimates and their standard errors. The raw data used for these statistical analyses can be found in Additional file 2: Table S1.

## Results

### Differences in climate between the elevation sites

The three elevation sites differed in climate. Across the 15 years of the study, the mean annual temperature was warmest at the low elevation site (15.4 °C), intermediate at the medium elevation site (14.3 °C), and coldest at the high elevation site (13.2 °C) and these differences were significant (ANOVA: *F*_(2, 42)_ = 9.431, *P* < 0.0001). The mean annual relative humidity was lowest at the low elevation site (61.9%), intermediate at the medium elevation site (63.7%), and highest at the high elevation site (66.5%), but these differences were not significant (ANOVA: *F*_(2, 42)_ = 1.948, *P* = 0.155). The mean annual saturation deficit was highest at the low elevation site (6.3 mmHg), intermediate at the medium elevation site (5.5 mmHg), and lowest at the high elevation site (4.6 mmHg), and these differences were significant (ANOVA: *F*_(2, 42)_ = 7.199, *P* = 0.002). These analyses show that the three elevation sites differed with respect to temperature, humidity and saturation deficit.

### Total number of ticks collected

Over the 15 years of the study, we collected a total of 39,255 *I. ricinus* ticks: 31,067 nymphs and 8188 adult ticks (4168 males and 4020 females) at the three lower elevation sites.

### Variation in nymph abundance among elevation sites

We used a one-way ANOVA to compare the mean CND between the three elevations (i.e. this analysis ignores the other explanatory variables). The interpretation of the CND is the theoretical number of questing nymphs that would have been collected in an area of 100 m^2^ over the course of the year if we had sampled the tick population each day (i.e. 365 sampling occasions per year). The mean CND was significantly different between the three elevation sites (ANOVA: *F*_(2, 42)_ = 6661.4, *P* < 0.0001). The mean CND (and 95% CI) for the low, medium, and high elevations were as follows: 21,532 (95% CI: 16,936–27,374), 17,793 (95% CI: 13,996–22,621), and 11,535 (95% CI: 9073–14,665). The CND can be converted to a daily mean number of nymphs per 100 m^2^ by dividing by 365 days. Thus, the mean number of nymphs collected in an area of 100 m^2^ (and 95% CI) for the low, medium, and high elevations were as follows: 59.0 (95% CI: 46.4–75.0), 48.7 (95% CI: 38.3–62.0), and 31.6 (95% CI: 24.9–40.2). In summary, the mean nymph density was inversely related to the altitudinal gradient and it was highest at the low elevation site and lowest at the high elevation site.

### Effect of the climate variables and mast years on variation in nymph abundance among years

For the log_10_-transformed CND, the best six models had a combined support of 95.0% (Table 1). The best model had 76.0% of the support (Table 1), explained 73.2% of the variation in the log_10_-transformed CND, and contained the explanatory variables of elevation site (*partial r*^2^ = 26.6%), year (*partial r*^2^ = 14.8%), beech mast score 2 years prior (*partial r*^2^ = 26.9%), and the field-collected relative humidity from the same year (i.e. no time lag; *partial r*^2^ = 7.6%). The five next-best models all contained the same explanatory variables of elevation site, year, and beech mast score, but differed with respect to the fourth explanatory variable, which included the site:year interaction, field-collected SD from the same year, field-collected temperature from the same year, field-collected RH from the previous year, and field-collected SD from the previous year (Table 1). The full model selection analysis with 52 models is presented in Additional file 1: Section 6.

**Table 1:** Model selection results for the linear models of the log_10_-transformed cumulative nymphal density

Rank	Model structure	*df*	logLik	AIC	ΔAIC	Weight1	Weight2	*r*^2^
1	log_10_(CND) ~ S+Y+B+RH2	7	33.0	− 48.7	0.0	76.0	76.0	73.2
2	log_10_(CND) ~ S+Y+B+RH2+S:Y	9	33.6	− 43.6	5.2	6.0	82.0	72.4
3	log_10_(CND) ~ S+Y+B+SD2	7	30.4	− 43.5	5.3	5.0	87.0	69.7
4	log_10_(CND) ~ S+Y+B+T2	7	30.4	− 43.4	5.3	5.0	92.0	69.6
5	log_10_(CND) ~ S+Y+B+RH2_y-1_	7	29.4	− 41.4	7.3	2.0	94.0	68.1
6	log_10_(CND) ~ S+Y+B+SD2_y-1_	7	29.1	− 40.8	7.9	1.0	95.0	67.7

*Notes*: Model selection results are shown for the linear models with normal errors of the log_10_-transformed CND response variable. The explanatory variables were elevation site, year, beech masting 2 years prior, and the climate variables from the field and the Climap-net database. The models are ranked according to their Akaike Information Criterion (AIC). Of the 52 models in the set, only the 6 top models are shown for which the cumulative support (Weight 2) is 95%. Shown for each model are the model rank (Rank), model structure (see below for explanation of variable acronyms), model degrees of freedom (Df), log-likelihood (logLik), Akaike information criterion (AIC), difference in the AIC value from the top model (ΔAIC), model weight (Weight1), cumulative model weight (Weight2), and adjusted r-squared value (*r*^2^). Additional file 1: Section 6 shows the results from the full model selection. The acronyms for the explanatory variables are as follows: S, site; Y, year; B, beech mast score 2 years prior; S:Y, interaction between site and year; RH2, mean annual relative humidity from the field data in the same year; SD2, mean annual saturation deficit from the field data in the same year; T2, mean annual temperature from the field data in the same year; RH2_y-1_, mean annual relative humidity from the field data in the previous year (y-1); SD2_y-1_, mean annual saturation deficit from the field data in the previous year (y-1)

Using the parameter estimates from the top models in Table 1, the effect sizes were calculated on the original scale of the CND with respect to the following baseline: the site was low elevation, the year was 2004, the beech tree mast index 2 years prior was set to 1, and the field-collected relative humidity from the same year was set to 50.0%. For simplicity, we present the parameter estimates from the top model in Table 1 (Additional file 1: Section 8), but we note that these are qualitatively similar to the model-averaged parameter estimates for the 52 models in the full model selection analysis (Additional file 1: Section 6).

**Fig. 1:**
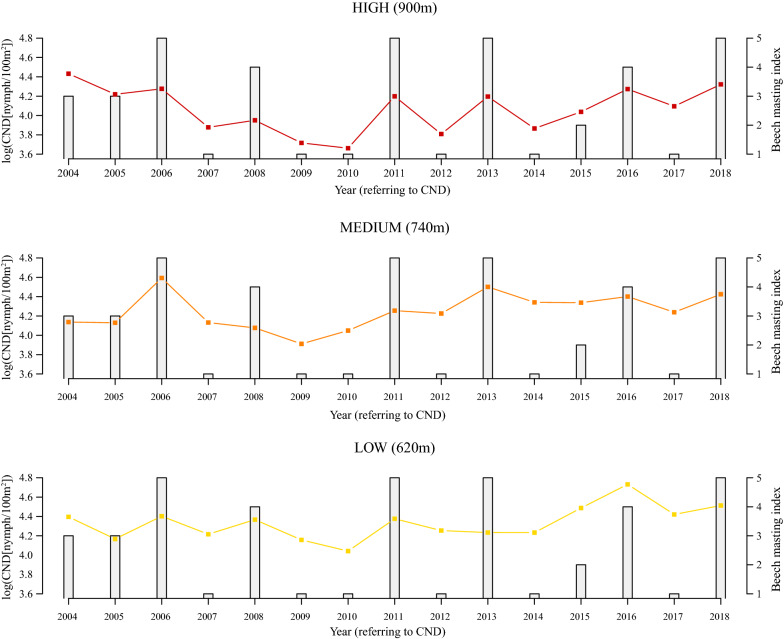
Annual variation in log_10_-transformed cumulative nymphal density (CND) and beech tree mast score 2 years prior. The log_10_-transformed CND (dots and solid lines) and the beech tree mast score 2 years prior (grey bars) are shown over time for each of the three elevation sites (low, medium, high) on Chaumont Mountain. The log_10_-transformed CND increased significantly over the 15-year study period (2004–2018). Years of high seed production by beech trees (beech masting index) are strongly positively correlated with high log_10_-transformed CND two years later. The CND is an estimate of the annual abundance of *I. ricinus* nymphs per 100 m^2^ and is calculated by integrating the area under the curve of the 12 monthly estimates of the number of questing nymphs collected by dragging an area of 100 m^2^. Beech tree mast scores range from 1 to 5 (1, very poor mast; 2, poor mast; 3, moderate mast; 4, good mast; and 5, full mast)

**Fig. 2:**
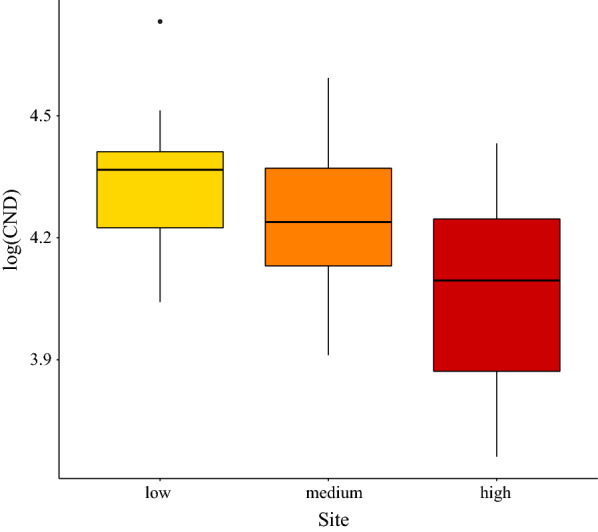
Effect of elevation on the log_10_-transformed cumulative nymphal density (CND). According to the model parameter estimates, the CND (on the original scale) at the low elevation was 11.4% higher than the medium elevation and 43.1% higher than the high elevation (partial *r*^2^ = 26.6%). The parameter estimates used to calculate the effect sizes were taken from the best model in Table 1, which had 76.0% of the support and explained 73.2% of the inter-annual variation in the log_10_-transformed CND

The log_10_-transformed CND was significantly different between the three elevation sites (Figs. 1 and 2; Additional file 1: Section 8). The CND (on the original scale) at the low elevation was 11.4% higher than the medium elevation (*Medium-Low contrast in Y-intercept* = -0.053, *SE* = 0.045, *P* = 0.253). The CDN (on the original scale) at the low elevation was 43.1% higher than the high elevation (*High-Low contrast in Y-intercept* = -0.245, *SE* = 0.005, *P* < 0.0001). In summary, the effect of elevation site in this multiple regression approach was the same as the results from the one-way ANOVA. The CND was inversely related to the altitudinal gradient; it was highest at the low elevation and lowest at the high elevation (Figs. 1 and 2; Additional file 1: Section 8).

**Fig. 3:**
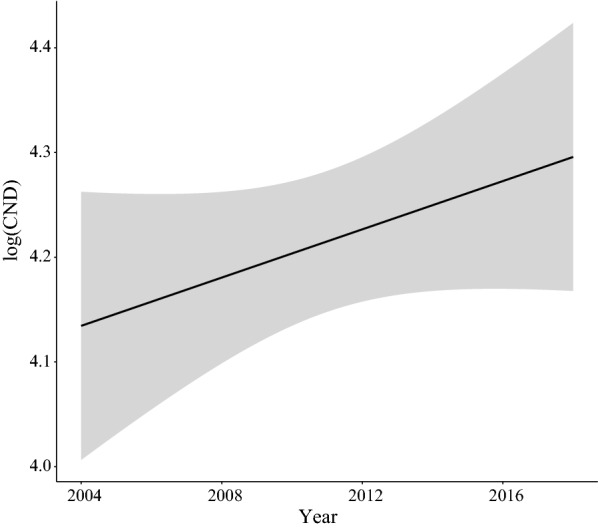
Effect of year on the log_10_-transformed cumulative nymphal density (CND). According to the model parameter estimates, the CND (on the original scale) increased by 88.4% over the 15-year study period (partial *r*^2^ = 14.8%). The parameter estimates used to calculate the effect sizes were taken from the best model in Table 1, which had 76.0% of the support and explained 73.2% of the inter-annual variation in the log_10_-transformed CND

The slope of the covariate year was positive (*slope* = 0.020 per year, *SE* = 0.005, *P* < 0.0001; Additional file 1: Section 8), indicating that the log_10_-transformed CND was increasing over time at the three elevation sites. According to the estimate of the general slope for the three elevation sites on Chaumont Mountain, the CND (on the original scale) increased by 88.4% over the 15-year period of the study (2004–2018) (Fig. 3).

**Fig. 4:**
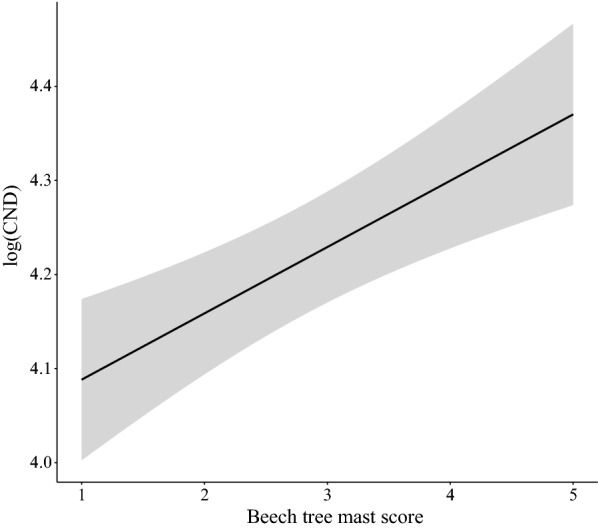
Effect of beech mast score 2 years prior on the log_10_-transformed cumulative nymphal density (CND). According to the model parameter estimates, increasing the beech mast score from 1 (poor mast) to 5 (full mast) increased the CND (on the original scale) by 86.2% (partial *r*^2^ = 26.9%). The parameter estimates used to calculate the effect sizes were taken from the best model in Table 1, which had 76.0% of the support and explained 73.2% of the inter-annual variation in the log_10_-transformed CND

The beech mast score had a strong positive effect on the log_10_-transformed CND two years later (*slope* = 0.067 per class, *SE* = 0.011, *P* < 0.0001; Fig. 1 and Additional file 1: Section 8). According to the estimate of the general slope for the three elevation sites on Chaumont Mountain, increasing the beech mast score from 1 (poor mast) to 5 (full mast) increased the CND (on the original scale) by 86.2% (Fig. 4). Over the 15-year study period, beech trees produced 6 years of good or full mast scores (2004, 2006, 2009, 2011, 2014 and 2016), where high CND years were expected to occur two years later (2006, 2008, 2011, 2013, 2016 and 2018, respectively). At the low, medium, and high site, these 6 good mast years produced 3 (2016, 2018 and 2006), 4 (2006, 2013, 2018 and 2016), and 4 (2018, 2006, 2016 and 2011) of the six-highest CND values two years later (Fig. 1). For the adult ticks, the beech tree mast score had a strong positive effect on the log_10_-transformed CAD three years later (*slope* = 0.101, *SE* = 0.019, *P* < 0.001, *partial r*^2^ = 21.0%; Additional file 1: Section 5).

**Fig. 5:**
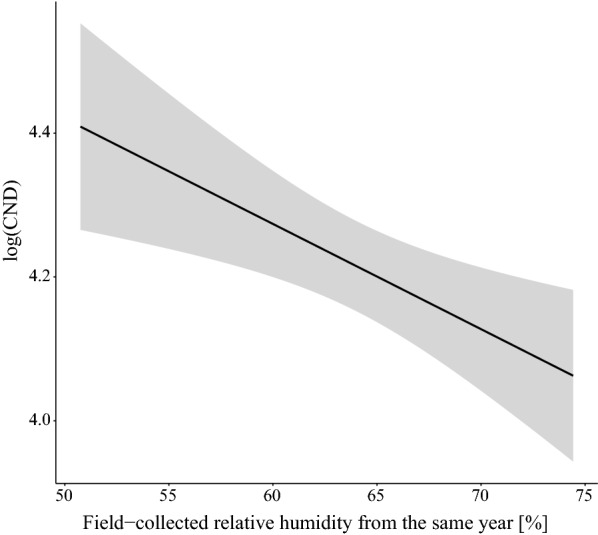
Effect of the field-collected mean annual relative humidity on the log_10_-transformed cumulative nymphal density (CND). According to the model parameter estimates, increasing the field-collected mean annual relative humidity from 50.0% to 75.0% decreased the CND from the same year (on the original scale) by 46.4% (partial *r*^2^ = 7.6%). The mean annual relative humidity was calculated for the same year as the CND (i.e. no time lag). The parameter estimates used to calculate the effect sizes were taken from the best model in Table 1, which had 76.0% of the support and explained 73.2% of the inter-annual variation in the log_10_-transformed CND

The field-collected mean annual relative humidity at 60 cm above ground had a negative effect on the log_10_-transformed CND in the same year (*slope* = -0.074 per standard deviation, *SE* = 0.021, *P* = 0.001; Additional file 1: Section 8). According to the estimate of the general slope for the three elevation sites on Chaumont Mountain, increasing the field-collected mean annual relative humidity from 50.0% to 75.0% decreased the CND from the same year (on the original scale) by 46.4% (Fig. 5).

The models with other climate variables had much less support (Table 1): field-collected mean annual SD from the same year (5%), field-collected mean annual temperature from the same year (5%), field-collected mean annual RH from the previous year (2%), and field-collected mean annual SD from the previous year (1%). We note here that field-collected mean annual RH (regardless of the time lag) always had a negative effect on nymph abundance, whereas field-collected mean annual temperature and field-collected mean annual SD always had a positive effect on tick abundance (Additional file 1: Section 6).

The analysis of the log_10_-transformed CND for the four elevation sites gave very similar results (Additional file 1: Section 4). The only difference was that the best model contained an interaction between elevation and year. This interaction was driven by the dramatic decline in CND over time at the top site. The best model for the four-site analysis contained the effects of elevation site, year, beech mast score, and mean annual relative humidity in the same year (i.e. no time lag), which is identical to the three-site analysis. The parameter estimates were also very similar between the 3-site and 4-site analyses. We believe that the dramatic decline in CND at the top site was caused by the construction of the recreation facilities during the study. For this reason, we presented the 3-site analysis of the CND in the main text. The analysis of the log_10_-transformed cumulative adult density (CAD) for all four elevation sites is also presented in Additional file 1: Section 5.

In summary, the CND increased significantly over time and with tree seed production two years earlier while it decreased significantly with elevation and with the field-collected relative humidity in the same year.

## Discussion

Climate change is driving the range expansion of arthropod vectors and the increased incidence of vector-borne diseases [3, 47]. In Europe and North America, there is currently much interest in determining which abiotic and biotic factors are influencing the abundance of *Ixodes* ticks and their associated pathogens. To address this question, we quantified the abundance of *I. ricinus* ticks over a period of 15 years in an area of Switzerland that is endemic for Lyme borreliosis. Variation in our annual estimates of cumulative nymph density was best explained by elevation site, year, abundance of beech nuts 2 years prior, and mean annual relative humidity in the same year. Nymph density increased by 88.4% (i.e. almost doubled) over the 15-year study period at the three elevation sites.

One of the most important results of this study is our demonstration of a strong positive association between seed production by beech trees and the density of nymphs two years later. The effect of beech seed production two years prior on the log_10_-transformed CND was highly statistically significant (*P* < 0.000001). According to our parameter estimates, the effect size was large; changing the beech mast score two years prior from 1 to 5 increased the CND by 86.2%. Beech nut abundance 2-years prior explained at least 26.9% of the variation in our annual estimates of nymph abundance. By accounting for large inter-annual fluctuations in the CND, the inclusion of the beech mast index in our models revealed the effects of more subtle ecological factors, such as mean annual relative humidity and year. In keeping with the slow life-cycle of *Ixodes* ticks, the beech seed production increased the abundance of adult ticks three years later (Additional file 1: Section 5). The synchronized seed production in beech trees is driven by climate variables such as temperature and precipitation [23–26]. Some recent studies have suggested that climate change is increasing the frequency of masting events [23, 25, 48]. If true, this suggests that climate change could be increasing tick abundance in endemic areas *via* indirect effects on tree seed production; more frequent masting events would increase the long-term abundance of reservoir hosts for ticks and tick-borne pathogens. Future long-term studies investigating whether climate change is affecting the abundance of *Ixodes* ticks and the incidence of tick-borne diseases should include the proper time-lagged estimates of tree seed production into their models.

The 2-year time lag between masting events and the abundance of nymphs was first discovered by Ostfeld and colleagues in the eastern USA where the blacklegged tick (*Ixodes scapularis*) is the vector of Lyme borreliosis [10, 31, 32]. The chain of causality is as follows. Mast seeding in year y increases the abundance of small mammals, deer, and larval ticks in year y + 1 [10, 27, 29, 31, 49, 50]. Higher densities of larval ticks coincide with and feed on higher densities of small mammals, which in turn increases the abundance of nymphs in year y + 2 [10, 31–33, 51–53]. In summary, there is good evidence in the eastern USA that mast events drive the abundance of *I. scapularis* nymphs with a 2-year time lag. In contrast, there are few studies in Europe that have demonstrated this phenomenon for *I. ricinus*. A 7-year study in Poland found the expected 2-year time lag between oak mast seeding and the incidence of Lyme borreliosis, but the causal link between tree seed production and nymphal abundance was not demonstrated [35]. A 10-year study in the Netherlands found that the mast scores of common oak trees and beech trees in year y had a negative effect on larvae, nymphs and adults in year y + 1 [34]. This result is expected because once ticks obtain a blood meal, they are no longer available to be sampled *via* dragging. An 18-year study in central Europe found a strong correlation between rodent densities in year y and tick-borne diseases in year y+1, but did not demonstrate a direct link to ticks or tree seed production [36]. Thus, our study is the first demonstration that mast seeding events are strongly associated with the inter-annual abundance of *I. ricinus* ticks in Europe, which in turn influences the risk of tick-borne diseases [35].

An important contribution of our study is the demonstration that only the 2-year time lag was important for explaining variation in the CND (Additional file 1; Section 9). Masting with a 2-year time lag had a *P*-value of < 0.000001. When the time lag between masting and the CND was changed to 0, 1, 3 or 4 years, the relationship was no longer statistically significant. Masting with a 2-year time lag had a partial *r*^2^ value of 26.3%. In contrast, for the other time lags (0, 1, 3 and 4 years in the past), the partial *r*^2^ values of masting ranged from 0.0 to 5.1%. This analysis clearly shows that variation in the CND at our study location was strongly associated with masting with a 2-year time lag and not with any other time lag.

Our long-term study is a rare demonstration that the abundance of *Ixodes* ticks has increased in a Lyme disease-endemic area. We found that the abundance of nymphs at the three lower elevation sites increased by 88.4% from 2004 to 2018. A 10-year study in the Netherlands found an increase in the abundance of *I. ricinus* larvae and adults [34]. In contrast, a 15-year study at a site in Neuchâtel, Switzerland, that is very close to the location of the present study found a significant decrease in the abundance of *I. ricinus* nymphs [11]. Another 8-year study in central New Jersey, USA, found no significant directional change in the abundance of *I. scapularis* nymphs [12]. Other long-term studies have found large inter-annual fluctuations in density, but none of them found that *Ixodes* tick density was increasing [10, 13, 14]. Increased tick abundance could have important consequences for the incidence of tick-borne diseases and public health. All else being equal, our study suggests that the risk of tick-borne disease has doubled at this location in Switzerland.

Theoretical models have predicted that *Ixodes* ticks will increase their distribution and abundance under global warming [6, 54–56]. Numerous empirical studies have shown that *Ixodes* ticks and tick-borne diseases are emerging public health problems at the northern limit of their geographic distribution [8, 57–59]. In contrast, fewer studies have investigated whether global warming has influenced *Ixodes* tick abundance in areas where Lyme disease is endemic [10–17]. The present study did not find direct links between climate change and the observed doubling in tick abundance. Mean annual relative humidity in the same year was the climate variable that had the greatest impact on the CND, but there was no evidence that it had undergone directional change over the 15-year study period (Additional file 1: Section 10). Conversely, the mean annual temperature increased significantly over the 15-year duration of the study (i.e. evidence of directional climate change; Additional file 1: Section 10), and while temperature had a significant and positive effect on the CND (Additional file 1: Section 6), models that contained temperature had low support (5.7%; Additional file 1: Section 6). In summary, despite finding a significant increase in temperature and ticks over the 15-year study period, the evidence for a causal link between the two is weak.

An interesting result is our demonstration that the mean annual relative humidity had a negative effect on the abundance of nymphs in the same year. This result was unexpected, as numerous studies have shown that survival of immature *Ixodes* ticks increases with relative humidity [18, 19, 60]. However, this result is not without precedent and other studies in Europe have found a negative relationship between relative humidity and the abundance of *I. ricinus* nymphs [61–65]. One explanation is that humid environments are favorable for the development of entomopathogenic fungi, which have been shown to cause high mortality in *Ixodes* ticks [66, 67]. A striking result was that the field-collected climate variables (measured at 60 cm above ground) were much more important for predicting tick abundance than climate variables from nearby weather stations (measured at 200 cm above ground). This result is even more remarkable when one considers that the annual means from the Climap-net data are based on 730 times more data (365 days × 24 hours = 8760 measurements per year) compared to the field-collected data (12 measurements per year). A recent long-term study at a nearby site in Neuchâtel [11] found the same result: field-collected climate variables were better than weather station climate variables at explaining inter-annual variation in tick abundance. The explanation is that the field-collected data measure the local conditions, whereas the weather stations are some distance away from the elevation sites. We suspect that microclimate factors like soil and vegetation are influencing the field-collected annual climate means to such an extent that they have only a weak resemblance to the weather station annual climate means (Additional file 1: Section 10). Our study suggests that researchers should make the effort to measure local climate variables rather than relying on data from nearby weather stations.

Ticks were found at all three elevations (620, 740 and 900 m ASL), but there were important differences in their abundance. Nymphal tick abundance was negatively correlated with elevation, which is in agreement with previous studies [39, 40]. A mechanistic explanation for this phenomenon is the relationship between temperature and tick development rates [18, 21]. At higher and colder elevations, eggs and larvae have much slower development rates, which ultimately reduces the number of larvae that reach the nymphal stage [20, 68, 69].

The nymphal tick abundance was lowest at the top site (1073 m ASL). At the top site, the CND decreased by 81.0% over the 15 years of the study, whereas at the other three elevation sites, the CND increased by 88.4% over the same time period (Additional file 1: Section 4). Thus, the population dynamics at the top site were very different from the low, medium, and high elevation sites. One explanation is the construction of recreation facilities at a distance of ~ 25 meters from our top site. These recreation facilities, which include a network of mountain bike trails (constructed from 2006 to 2010) and an adventure park with a zip line and outdoor laser games (constructed in 2011), have greatly increased the number of human visitors to the top of Chaumont Mountain. Thus, the destruction of the forest habitat and subsequent human disturbance may have caused the decline of the *I. ricinus* tick populations over time at our top site. An interesting alternative explanation is that the monthly tick sampling over a period of 15 years decreased the CND at the top site. Field studies typically assume that dragging removes a small fraction of the available tick population, but this assumption may not be true in habitats where tick density is already low.

## Conclusions

We found that the abundance of *I. ricinus* nymphs almost doubled over 15 years at our study location in Switzerland. From a public health perspective, this increase in nymph abundance is likely to have increased the risk of tick-borne disease in this region. Beech mast years at 2 and 3 years prior were strongly and positively associated with the abundance of nymphs and adult ticks, respectively. We found that relative humidity had a negative effect on nymph abundance, which was surprising because immature *Ixodes* ticks are known to be sensitive to desiccation. We found no direct association between climate change and the doubling of nymphal tick abundance. However, there could be indirect effects if climate change is increasing the frequency of masting events and thereby increasing the abundance of reservoir hosts and ticks. Public health officials should be aware that mast years are an important time-lagged predictor of tick abundance and the incidence of tick-borne diseases.

## Supplementary information

**Additional file 1: Section 1.** Validation of the field-collected temperature data. **Section 2.** Validation of the field-collected relative humidity data. **Section 3.** Interpolation of the Climap-net climate data. **Section 4.** Full statistical analysis of the cumulative nymph abundance (CND) for the four elevation sites. **Section 5.** Full statistical analysis of the cumulative adult abundance (CAD) for the four elevation sites. **Section 6.** Full model selection table, support of each individual explanatory variable, and model-averaged parameter estimates of the nymph abundance for the three lowest elevation sites. **Section 7.** Assumptions of the linear models for the best models from the AIC-based model selection approach of the nymph and adult abundance. **Section 8.** Parameter estimates of the top model in the model selection table of the nymph abundance for the three lowest elevation sites. **Section 9.** Analysis of different time lags between the cumulative nymph abundance and beech masting. **Section 10.** Climate change over the 15-year study period. **Section 11.** Analysis of CND using Generalized Linear Models with negative binomial errors.

**Additional file 2: Table S1.** Raw data used for all statistical analyses.

## Data Availability

The raw data for this study are stored in the Additional file 2. The climate data are available from the Climap-net database of the Federal Office for Meteorology and Climatology (http://www.meteosuisse.admin.ch/home/service-et-publications/conseil-et-service/portail-de-donnees-dedie-aux-specialistes.html. Accessed 4 Mar 2019). The tree seed production dataset is available in the Ecology–Ecological Society of America repository (http://onlinelibrary.wiley.com/doi/10.1002/ecy.1785/suppinfo).

## Abbreviations

AIC: Akaike information criterion
ASL: above sea level
CAD: cumulative adult density
CND: cumulative nymph density
LM: linear models
RH: relative humidity
SD: saturation deficit
SE: standard error
