# Supplementary material for: Climate and tree seed production predict the abundance of the European Lyme disease vector over a 15-year period
Source: Parasit Vectors. 2020 Aug 10;13:408. doi: 10.1186/s13071-020-04291-z (PMC7418309; doi:10.1186/s13071-020-04291-z)
Supplement: Supplementary file 1 — Additional file 1: Section 1. Validation of the field-collected temperature data. Section 2. Validation of the field-collected relative humidity data. Section 3. Interpolation of the Climap-net climate data. Section 4. Full statistical analysis of the cumulative nymph abundance (CND) for the four elevation sites. Section 5. Full statistical analysis of the cumulative adult abundance (CAD) for the four elevation sites. Section 6. Full model selection table, support of each individual explanatory variable, and model-averaged parameter estimates of the nymph abundance for the three lowest elevation sites. Section 7. Assumptions of the linear models for the best models from the AIC-based model selection approach of the nymph and adult abundance. Section 8. Parameter estimates of the top model in the model selection table of the nymph abundance for the three lowest elevation sites. Section 9. Analysis of different time lags between the cumulative nymph abundance and beech masting. Section 10. Climate change over the 15-year study period. Section 11. Analysis of CND using Generalized Linear Models with negative binomial errors. [file 13071_2020_4291_MOESM1_ESM.docx]

**Additional file 1**

**Climate and tree seed production predict the abundance of the European Lyme disease vector over a 15-year period**

Cindy Bregnard, Olivier Rais, and Maarten J. Voordouw

**Table of Contents**

[SECTION 1 - Validation of the field-collected temperature data 2](#_Toc40700114)

[SECTION 2 - Validation of the field-collected relative humidity data 9](#_Toc40700115)

[SECTION 3 – Interpolation of the Climap-net climate data 16](#_Toc40700116)

[SECTION 4 – Full statistical analysis of the cumulative nymph abundance (CND) for the four elevation sites 18](#_Toc40700117)

[SECTION 5 – Full statistical analysis of the cumulative adult abundance (CAD) for the four elevation sites 30](#_Toc40700118)

[SECTION 6 – Full model selection table, support of each individual explanatory variable, and model-averaged parameter estimates of the nymph abundance for the three lowest elevation sites 42](#_Toc40700119)

[SECTION 7 – Assumptions of the linear models for the best models from the AIC-based model selection approach of the nymph and adult abundance for the three lowest elevation sites 52](#_Toc40700120)

[SECTION 8 – Parameter estimates of the top model in the model selection table of the nymph abundance for the three lowest elevation sites 53](#_Toc40700121)

[SECTION 9 – Time lag between the nymph abundance and beech masting 54](#_Toc40700122)

[SECTION 10 – Climate change over the 15-year study period 60](#_Toc40700123)

[SECTION 11 – Analysis of CND using Generalized Linear Models with Negative Binomial Errors 74](#_Toc40700124)

# SECTION 1 - Validation of the field-collected temperature data

Methods – Correlation between field-collected and Climap-net data: To validate our field-collected temperature data, we compared it to the Climap-net data that we obtained from two weather stations that are close to our four elevation sites and that are located at 485 m ASL in Neuchâtel and at 1136 m ASL in Chaumont. We used Pearson’s correlation tests to show that the mean daily temperature was correlated between the field-collected data and each of the two weather stations. We also used a paired samples t-test to determine the difference in the mean daily temperature between our field-collected data and each of the two weather stations.

**Results – Correlation between field-collected data and Climap-net data:** Across all four elevation sites, the mean daily temperature for the field-collected data and the Neuchâtel weather station was positively correlated (Pearson’s r = 0.907, n = 597, p < 0.001; Figure S1; Table S1). The daily mean temperature from the field-collected data was 13% higher than the Neuchâtel weather station data (mean difference ± standard error: 2.0 ± 0.01°C), and this difference was significant (Paired sample t-test: df = 596, t = -17.181, p < 0.001). Taking each elevation site independently, the mean daily temperature was positively correlated between the field-collected data and the Neuchâtel weather station (Figure S2; Table S1). The absolute difference in mean daily temperature between the field-collected data and the Neuchâtel weather station increased from the top site (0.3 °C) to the low elevation site (3.9 °C; Table S2). Across all four elevation sites, the mean daily temperature for the field-collected data and the Chaumont weather station was positively correlated (Pearson’s r = 0.914, n = 597, p < 0.001; Figure S1; Table S1). The daily mean temperature from the field-collected data was 34% higher than the Chaumont weather station data (mean difference ± standard error: 5.4 ± 0.11°C), and this difference was significant (Paired sample t-test: df = 596, t = -47.908, p < 0.001). Taking each elevation site independently, the mean daily temperature was positively correlated between the field-collected data and the Chaumont weather station (Figure S3; Table S1). The absolute difference in mean daily temperature between the field-collected data and the Chaumont weather station increased from the top site (3.0 °C) to the low elevation site (7.1 °C; Table S3). In summary, the strong correlations between the mean daily temperature for field-collected data and each of the two weather stations validate the use of our field-collected temperature data.

Table S1. The correlation in the daily mean temperature between the field-collected data and the Climap-net data is shown for each of the four elevation sites and the two weather stations. The four sites were located on the south side of Chaumont Mountain. The two weather stations in Neuchâtel and Chaumont were located at 485 m ASL and 1136 m ASL, respectively. The mean daily temperature for the field-collected data and the two weather stations were measured at 60 cm above ground and 200 cm above ground, respectively. Shown are Pearson’s correlation coefficient (r), the sample size (n), and the statistical significance of the correlation (p).

|  |  | **Neuchâtel^b^** |  |  | **Chaumont^c^** |  |
| --- | --- | --- | --- | --- | --- | --- |
| **Site** | **r** | **n** | **p** | **r** | **n** | **p** |
| Top | 0.907 | 142 | < 0.001 | 0.939 | 142 | < 0.001 |
| High | 0.924 | 147 | < 0.001 | 0.949 | 147 | < 0.001 |
| Medium | 0.946 | 154 | < 0.001 | 0.954 | 154 | < 0.001 |
| Low | 0.950 | 154 | < 0.001 | 0.928 | 154 | < 0.001 |
| All^a^ | 0.907 | 597 | < 0.001 | 0.914 | 597 | < 0.001 |

^a^ The daily mean temperatures were positively correlated between the field-collected data and the Climap-net data from both weather stations.

^b^ The mean daily temperature from the field-collected data was 13% higher than the Climap-net data from the Neuchâtel weather station (mean difference ± standard error: 2.0 ± 0.01 °C), and this difference was significant (Paired sample t-test: df = 596, t = -17.181, p < 0.001).

^c^ The mean daily temperature from the field-collected data was 34% higher than the Climap-net data from the Chaumont weather station (mean difference ± standard error: 5.4 ± 0.11 °C), and this difference was significant (Paired sample t-test: df = 596, t = -47.908, p < 0.001).

Table S2. Comparison of the mean daily temperature between the field-collected data and the Climap-net data from the Neuchâtel weather station is shown for each of the four elevation sites. The mean difference in temperature is calculated as the field-collected mean minus the Neuchâtel Climap-net mean. Shown are the mean temperatures for the two sources of data and the results of the paired sample t-test for each of the four elevation sites.

| **Site** | **Source** | **Mean (°C)** | **Diff (°C)** | **s.e. (°C)** | **t** | **df** | **p** |
| --- | --- | --- | --- | --- | --- | --- | --- |
| Top | Field-collected | 13.9 | -0.3 | 0.28 | 1.282 | 141 | 0.202 |
|  | Neuchâtel Climap-net | 14.2 |  |  |  |  |  |
| High | Field-collected | 15.7 | 1.7 | 0.19 | -8.093 | 146 | < 0.001 |
|  | Neuchâtel Climap-net | 14.0 |  |  |  |  |  |
| Medium | Field-collected | 16.1 | 2.7 | 0.13 | -14.622 | 153 | < 0.001 |
|  | Neuchâtel Climap-net | 13.4 |  |  |  |  |  |
| Low | Field-collected | 17.2 | 3.9 | 0.23 | -20.387 | 153 | < 0.001 |
|  | Neuchâtel Climap-net | 13.3 |  |  |  |  |  |

Table S3. Comparison of the mean daily temperature between the field-collected data and the Climap-net data from the Chaumont weather station is shown for each of the four elevation sites. The mean difference in temperature is calculated as the field-collected mean minus the Chaumont Climap-net mean. Shown are the mean temperatures for the two sources of data and the results of the paired sample t-test for each of the four elevation sites.

| **Site** | **Source** | **Mean** | **Diff** | **s.e.** | **t** | **df** | **p** |
| --- | --- | --- | --- | --- | --- | --- | --- |
| Top | Field-collected | 13.9 | 3.0 | 0.15 | -17.853 | 141 | < 0.001 |
|  | Chaumont Climap-net | 10.9 |  |  |  |  |  |
| High | Field-collected | 15.7 | 5.0 | 0.12 | -31.278 | 146 | < 0.001 |
|  | Chaumont Climap-net | 10.7 |  |  |  |  |  |
| Medium | Field-collected | 16.1 | 6.0 | 0.24 | -34.903 | 153 | < 0.001 |
|  | Chaumont Climap-net | 10.1 |  |  |  |  |  |
| Low | Field-collected | 17.1 | 7.1 | 0.21 | -31.332 | 153 | < 0.001 |
|  | Chaumont Climap-net | 10.0 |  |  |  |  |  |


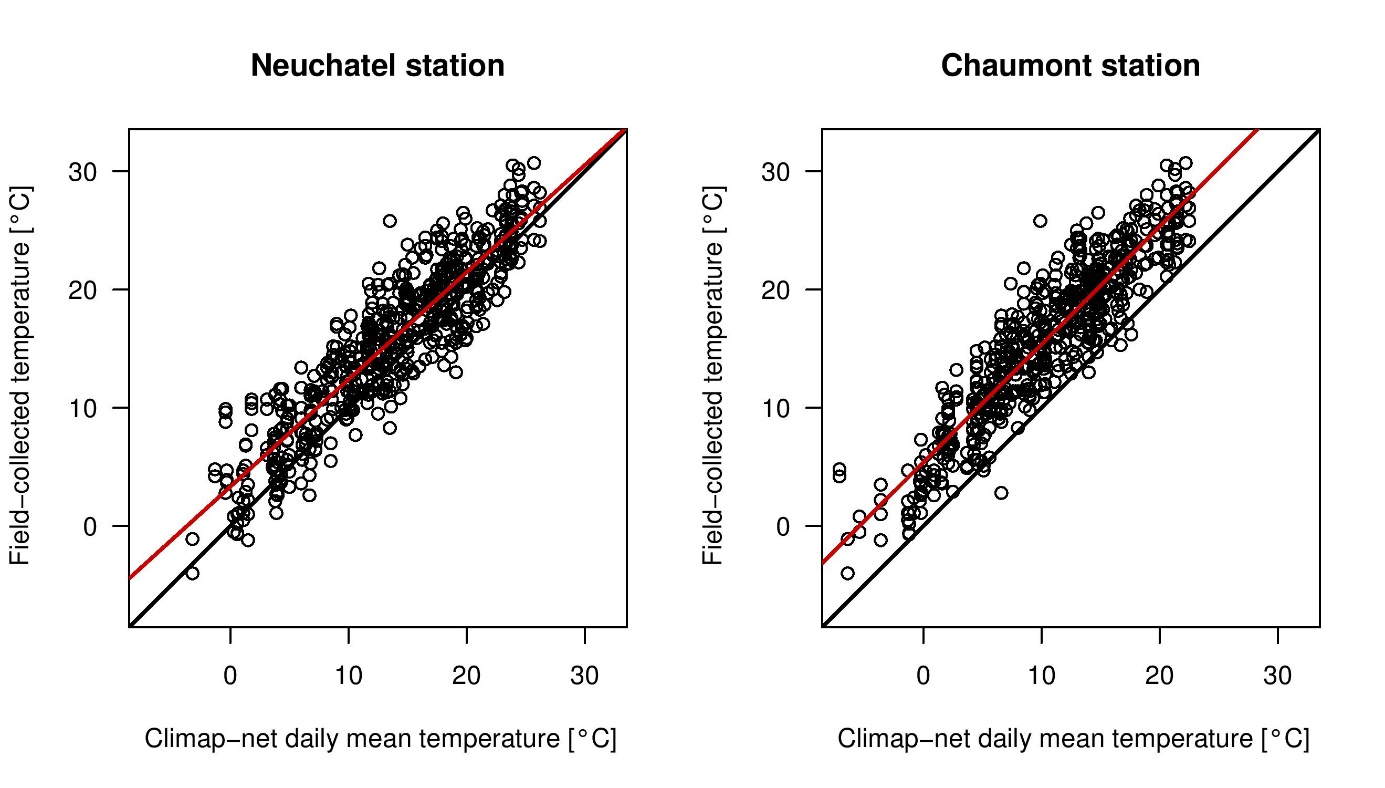
Figure S1. Linear regression of the mean daily temperature between the field-collected data and the Climap-net temperature data is shown for each of two weather stations: Neuchâtel (left panel) and Chaumont (right panel). The field-collected temperature data were sampled at four different elevation sites on Chaumont Mountain: low, medium, high, and top elevation site with an altitude of 620, 740, 900, and 1073, m ASL, respectively. The two weather stations in Neuchâtel and Chaumont were located at 485 m ASL and 1136 m ASL, respectively. The mean daily temperature for the field-collected data and the two weather stations were measured at 60 cm above ground and 200 cm above ground, respectively. The black line represents the 1:1 slope, whereas the red line represents the line of best fit from the linear regression.


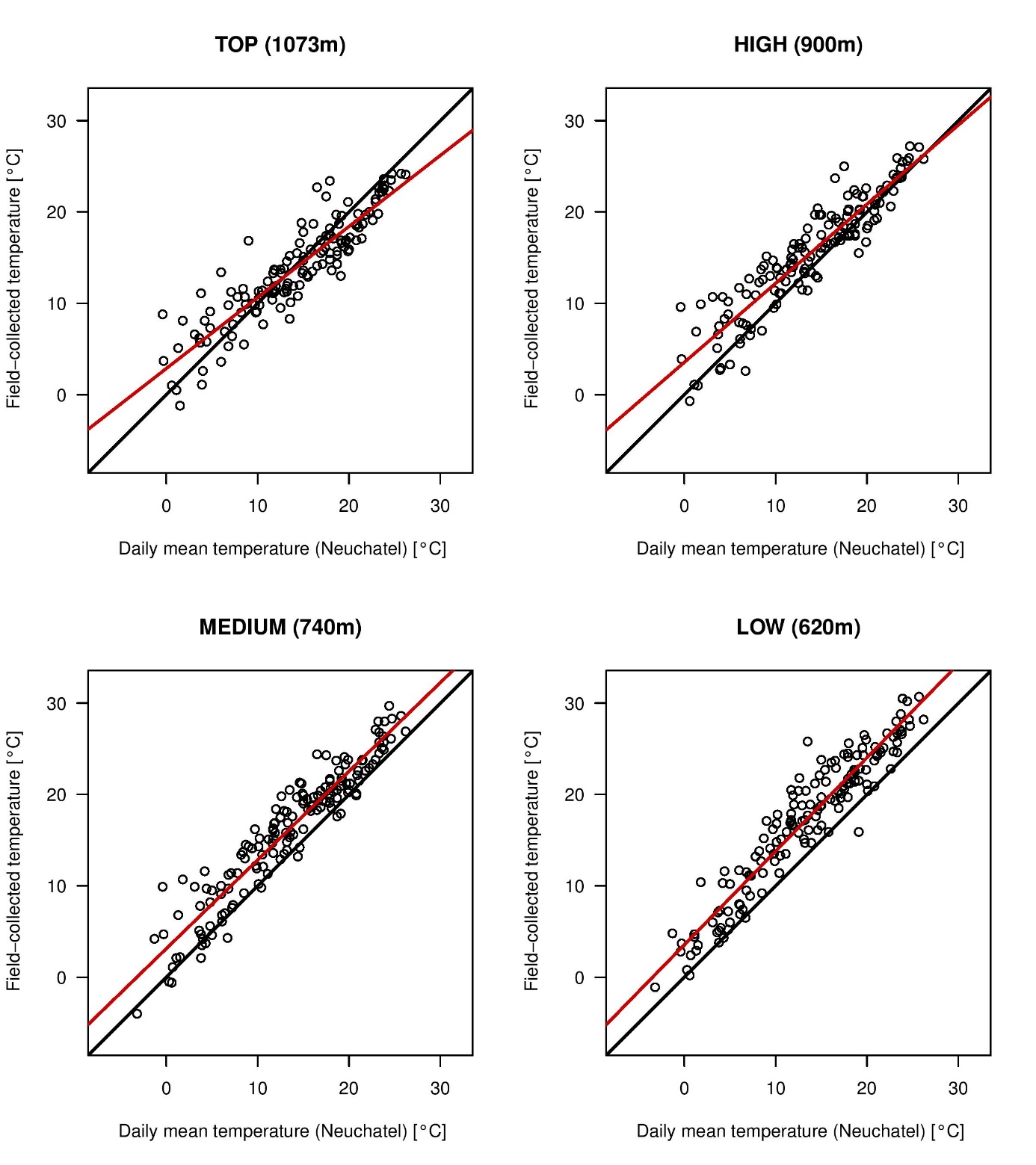
Figure S2. Linear regression of the mean daily temperature between the field-collected data and Climap-net temperature data from the Neuchâtel weather station is shown for each site. The field-collected temperature data were sampled at four different elevation sites on Chaumont Mountain: low, medium, high, and top elevation site with an altitude of 620, 740, 900, and 1073, m ASL, respectively. The weather station in Neuchâtel was located at 485 m ASL. The mean daily temperature for the field-collected data and the weather station were measured at 60 cm above ground and 200 cm above ground, respectively. The black line represents the 1:1 slope, whereas the red line represents the line of best fit from the linear regression.


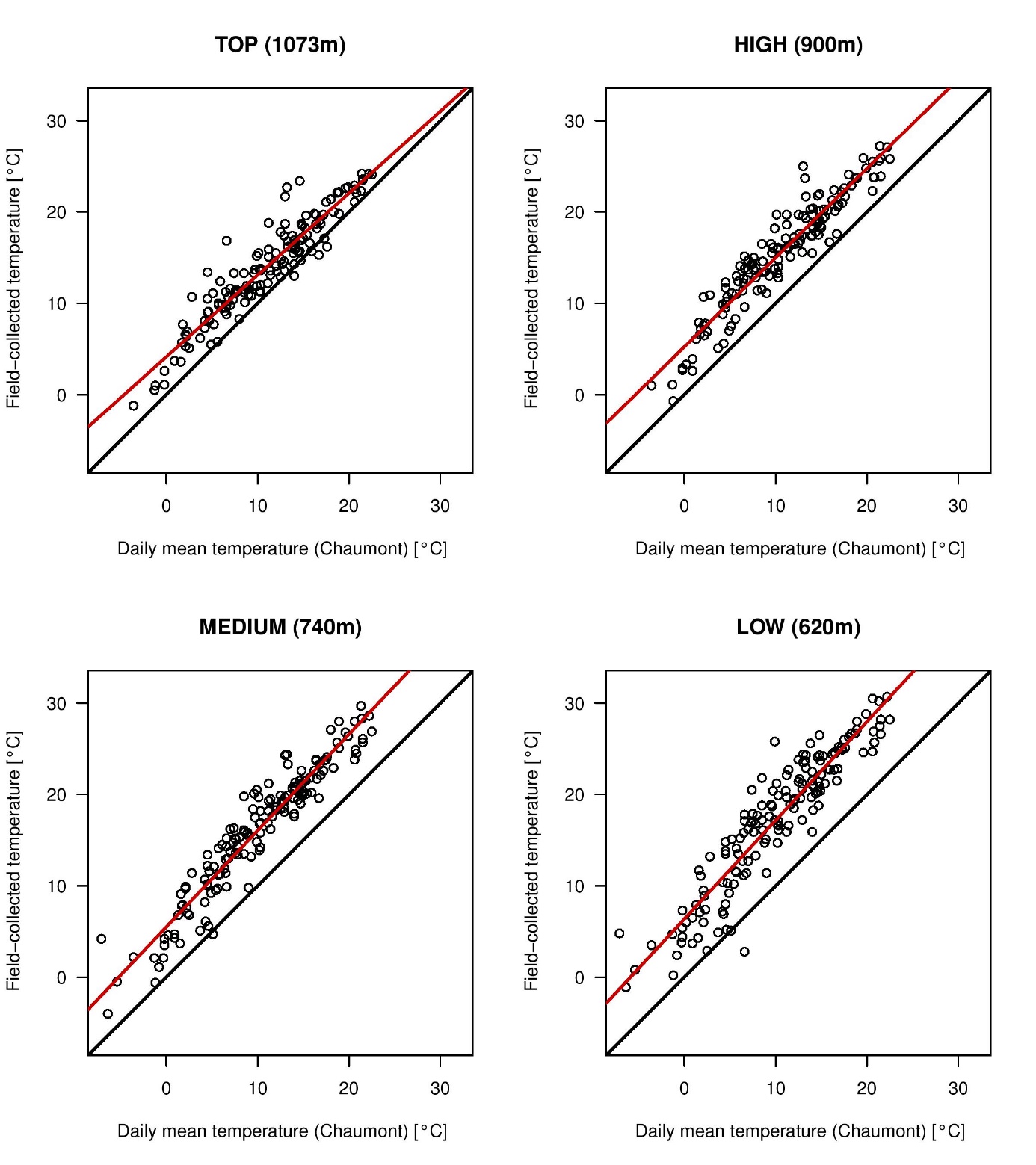
Figure S3. Linear regression of the mean daily temperature between the field-collected data and Climap-net temperature data from the Chaumont weather station is shown for each site. The field-collected temperature data were sampled at four different elevation sites on Chaumont Mountain: low, medium, high, and top elevation site with an altitude of 620, 740, 900, and 1073, m ASL, respectively. The weather station in Chaumont was located at 1136 m ASL. The mean daily temperature for the field-collected data and the weather station were measured at 60 cm above ground and 200 cm above ground, respectively. The black line represents the 1:1 slope, whereas the red line represents the line of best fit from the linear regression.

# SECTION 2 - Validation of the field-collected relative humidity data

Methods – Correlation between field-collected and Climap-net data: To validate our field-collected relative humidity data, we compared it to the Climap-net data that we obtained from two weather stations that are close to our four elevation sites and that are located at 485 m ASL in Neuchâtel and at 1136 m ASL in Chaumont. We used Pearson’s correlation tests to show that the mean daily relative humidity was correlated between the field-collected data and each of the two weather stations. We also used a paired samples t-test to determine the difference in the mean daily relative humidity between our field-collected data and each of the two weather stations.

**Results – Correlation between field-collected data and Climap-net data:** Across all four elevation sites, the mean daily relative humidity for the field-collected data and the Neuchâtel weather station was positively correlated (Pearson’s r = 0.597, n = 571, p < 0.001; Figure S4; Table S4). The daily mean relative humidity from the field-collected data was 8% lower than the Neuchâtel weather station data (mean difference ± standard error: -5.1 ± 0.41%), and this difference was significant (Paired sample t-test: df = 570, t = 9.523, p < 0.001). Taking each elevation site independently, the mean daily relative humidity was positively correlated between the field-collected data and the Neuchâtel weather station (Figure S5; Table S4). The absolute difference in mean daily relative humidity between the field-collected data and the Neuchâtel weather station increased from the top site (0.1%) to the low elevation site (9.1%; Table S5). Across all four elevation sites, the mean daily relative humidity for the field-collected data and the Chaumont weather station was positively correlated (Pearson’s r = 0.623, n = 571, p < 0.001; Figure S4; Table S4). The daily mean relative humidity from the field-collected data was 10% lower than the Chaumont weather station data (mean difference ± standard error: -6.4 ± 0.38%), and this difference was significant (Paired sample t-test: df = 570, t = 11.937, p < 0.001). Taking each elevation site independently, the mean daily relative humidity was positively correlated between the field-collected data and the Chaumont weather station (Figure S6; Table S4). The absolute difference in mean daily relative humidity between the field-collected data and the Chaumont weather station increased from the top site (1.2%) to the low elevation site (10.4%; Table S6). In summary, the strong correlations between the mean daily relative humidity for field-collected data and each of the two weather stations validate the use of our field-collected relative humidity data.

Table S4. The correlation in the daily mean relative humidity between the field-collected data and the Climap-net data is shown for each of the four elevation sites and the two weather stations. The four sites were located on the south side of Chaumont Mountain. The two weather stations in Neuchâtel and Chaumont were located at 485 m ASL and 1136 m ASL, respectively. The mean daily relative humidity for the field-collected data and the two weather stations were measured at 60 cm above ground and 200 cm above ground, respectively. Shown are Pearson’s correlation coefficient (r), the sample size (n), and the statistical significance of the correlation (p).

|  |  | **Neuchâtel^b^** |  |  | **Chaumont^c^** |  |
| --- | --- | --- | --- | --- | --- | --- |
| **Site** | **r** | **n** | **P** | **r** | **n** | **p** |
| Top | 0.376 | 135 | < 0.001 | 0.639 | 135 | < 0.001 |
| High | 0.516 | 141 | < 0.001 | 0.665 | 141 | < 0.001 |
| Medium | 0.704 | 148 | < 0.001 | 0.665 | 148 | < 0.001 |
| Low | 0.800 | 147 | < 0.001 | 0.603 | 147 | < 0.001 |
| All^a^ | 0.597 | 571 | < 0.001 | 0.623 | 571 | < 0.001 |

^a^ The daily mean relative humidity were positively correlated between the field-collected data and the Climap-net data from both weather stations.

^b^ The daily mean relative humidity from the field-collected data was 8% lower than the Climap-net data from the Neuchâtel weather station (mean difference ± standard error: -5.1 ± 0.41%), and this difference was significant (Paired sample t-test: df = 570, t = 9.523, p < 0.001).

^c^ The daily mean relative humidity from the field-collected data was 10% lower than the Climap-net data from the Chaumont weather station (mean difference ± standard error: -6.4 ± 0.38%), and this difference was significant (Paired sample t-test: df = 570, t = 11.937, p < 0.001).

Table S5. Comparison of the mean daily relative humidity between the field-collected data and the Climap-net data from the Neuchâtel weather station is shown for each of the four elevation sites. The mean difference in relative humidity is calculated as the field-collected mean minus the Neuchâtel Climap-net mean. Shown are the mean relative humidity for the two sources of data and the results of the paired sample t-test for each of the four elevation sites.

| **Site** | **Source** | **Mean** | **Diff** | **s.e.** | **T** | **df** | **p** |
| --- | --- | --- | --- | --- | --- | --- | --- |
| Top | Field-collected | 68.1 | -0.1 | 0.71 | 0.069 | 134 | 0.945 |
|  | Neuchâtel Climap-net | 68.2 |  |  |  |  |  |
| High | Field-collected | 64.1 | -4.2 | 0.60 | 3.715 | 140 | < 0.001 |
|  | Neuchâtel Climap-net | 68.3 |  |  |  |  |  |
| Medium | Field-collected | 61.5 | -7.2 | 0.80 | 7.657 | 147 | < 0.001 |
|  | Neuchâtel Climap-net | 68.7 |  |  |  |  |  |
| Low | Field-collected | 59.6 | -9.1 | 0.96 | 10.668 | 146 | < 0.001 |
|  | Neuchâtel Climap-net | 68.7 |  |  |  |  |  |

Table S6. Comparison of the mean daily relative humidity between the field-collected data and the Climap-net data from the Chaumont weather station is shown for each of the four elevation sites. The mean difference in relative humidity is calculated as the field-collected mean minus the Chaumont Climap-net mean. Shown are the mean relative humidity for the two sources of data and the results of the paired sample t-test for each of the four elevation sites.

| **Site** | **Source** | **Mean** | **Diff** | **s.e.** | **t** | **df** | **p** |
| --- | --- | --- | --- | --- | --- | --- | --- |
| Top | Field-collected | 68.1 | -1.2 | 0.66 | 1.168 | 134 | 0.245 |
|  | Chaumont Climap-net | 69.3 |  |  |  |  |  |
| High | Field-collected | 64.1 | -5.3 | 0.50 | 5.525 | 140 | < 0.001 |
|  | Chaumont Climap-net | 69.4 |  |  |  |  |  |
| Medium | Field-collected | 61.5 | -8.5 | 0.73 | 8.421 | 147 | < 0.001 |
|  | Chaumont Climap-net | 70.0 |  |  |  |  |  |
| Low | Field-collected | 59.6 | -10.4 | 0.91 | 8.967 | 146 | < 0.001 |
|  | Chaumont Climap-net | 70.0 |  |  |  |  |  |


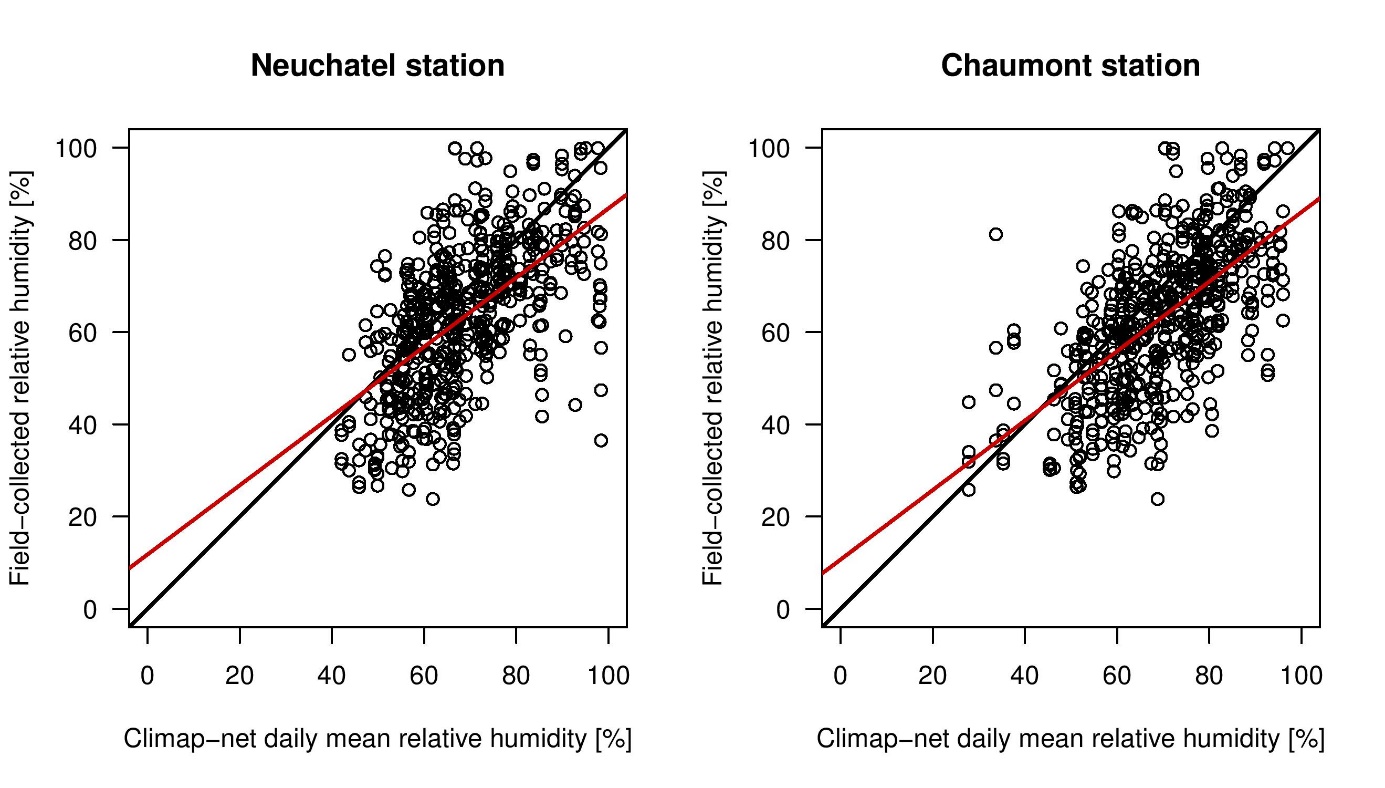
Figure S4. Linear regression of the mean daily relative humidity between the field-collected data and the Climap-net relative humidity data is shown for each of two weather stations: Neuchâtel (left panel) and Chaumont (right panel). The field-collected relative humidity data were sampled at four different elevation sites on Chaumont Mountain: low, medium, high, and top elevation site with an altitude of 620, 740, 900, and 1073, m ASL, respectively. The two weather stations in Neuchâtel and Chaumont were located at 485 m ASL and 1136 m ASL, respectively. The mean daily relative humidity for the field-collected data and the two weather stations were measured at 60 cm above ground and 200 cm above ground, respectively. The black line represents the 1:1 slope, whereas the red line represents the line of best fit from the linear regression.


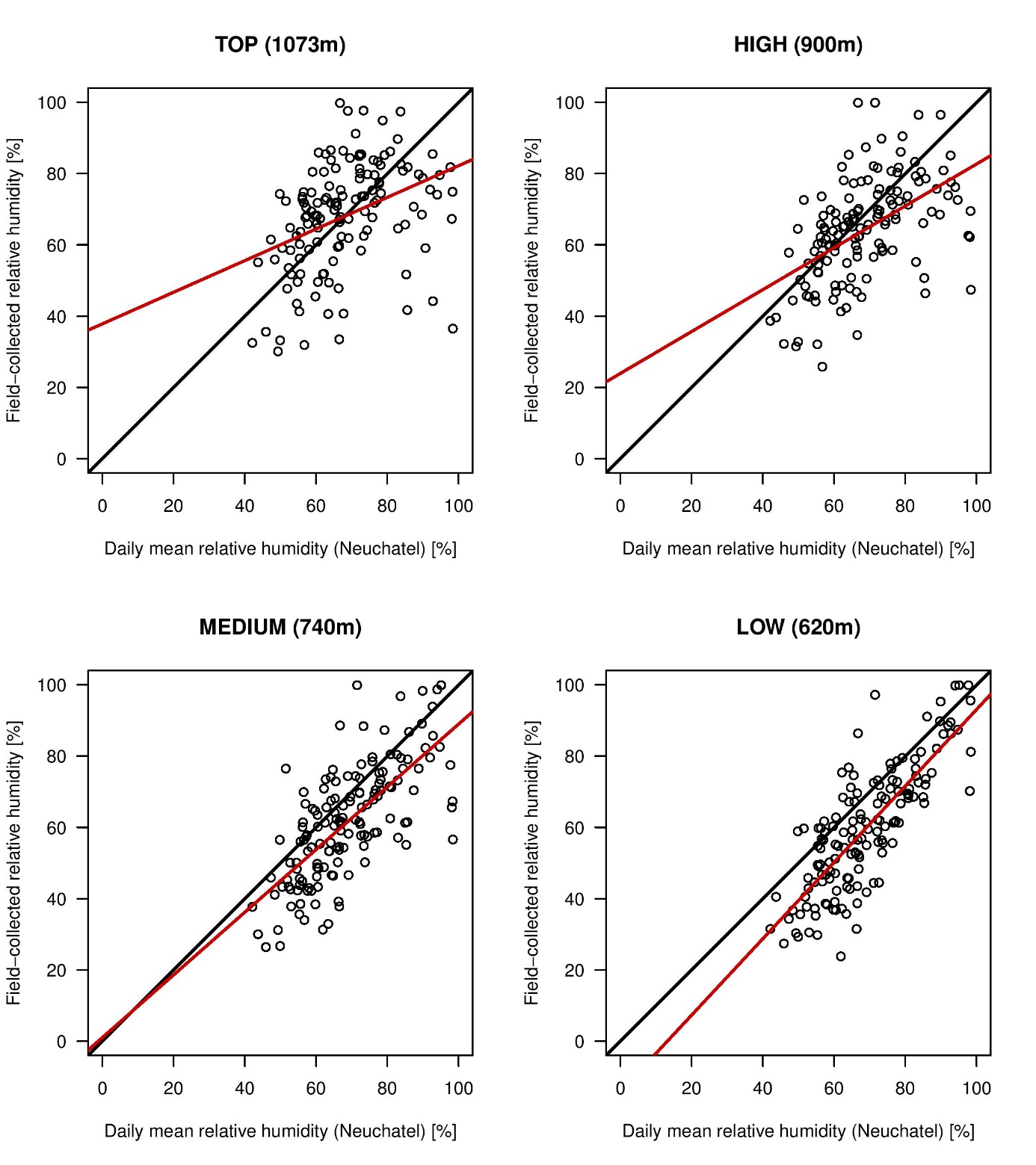
Figure S5. Linear regression of the mean daily relative humidity between the field-collected data and Climap-net relative humidity data from the Neuchâtel weather station is shown for each site. The field-collected relative humidity data were sampled at four different elevation sites on Chaumont Mountain: low, medium, high, and top elevation site with an altitude of 620, 740, 900, and 1073, m ASL, respectively. The weather station in Neuchâtel was located at 485 m ASL. The mean daily relative humidity for the field-collected data and the weather station were measured at 60 cm above ground and 200 cm above ground, respectively. The black line represents the 1:1 slope, whereas the red line represents the line of best fit from the linear regression.


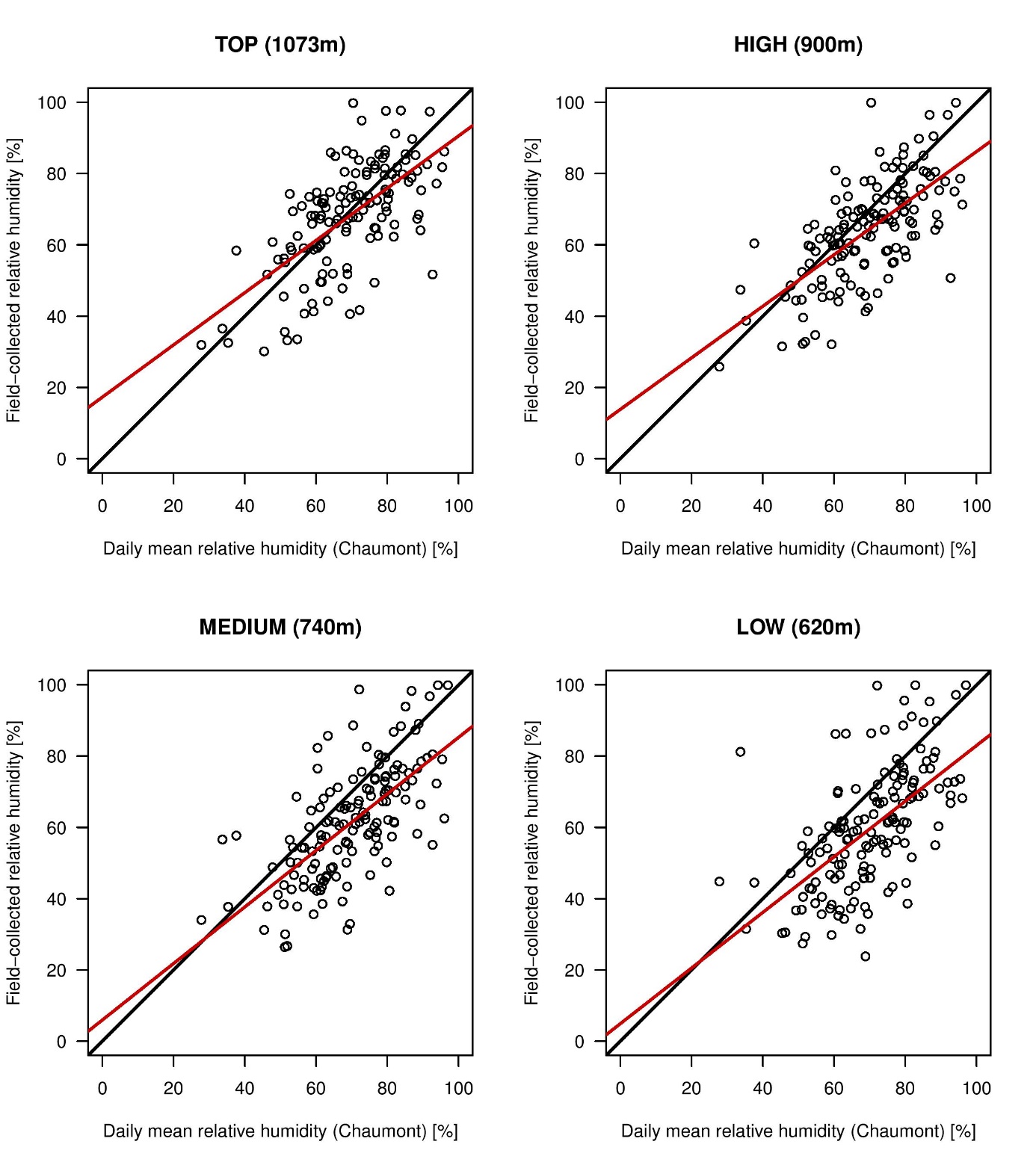
Figure S6. Linear regression of the mean daily relative humidity between the field-collected data and Climap-net relative humidity data from the Chaumont weather station is shown for each site. The field-collected relative humidity data were sampled at four different elevation sites on Chaumont Mountain: low, medium, high, and top elevation site with an altitude of 620, 740, 900, and 1073, m ASL, respectively. The weather station in Chaumont was located at 1136 m ASL. The mean daily relative humidity for the field-collected data and the weather station were measured at 60 cm above ground and 200 cm above ground, respectively. The black line represents the 1:1 slope, whereas the red line represents the line of best fit from the linear regression.

# SECTION 3 – Interpolation of the Climap-net climate data

**Methods:** Climap-net data were obtained from two weather stations that are close to our four elevation sites and that are located at 485 m ASL in Neuchâtel and at 1136 m ASL in Chaumont. To create a climate profile that was specific for each of the four elevation sites, we interpolated the values between the two weather stations using the relative elevation distance of each elevation site to the two weather stations. For example, the total elevation distance between the Neuchâtel and Chaumont weather stations is 651 meters, and the elevation distance between the top site and the Neuchâtel weather station is 620 meters, which represents 95.2% of the elevation distance. Thus, the climate at the top site is expected to be more similar to the Chaumont weather station (95.2%) compared to the Neuchâtel weather station (4.8%), whereas the reverse would be true for the low site. For each elevation site, the mean daily temperature, relative humidity, saturation deficit, and precipitation were calculated based on the interpolating percentages (Table S7).

Table S7. Climap-net data interpolation. Shown are the site, elevation, elevation distance with the Neuchâtel weather station (Dist 1), elevation distance between Neuchâtel and Chaumont weather station (Dist 2), the interpolating percentage from the Neuchâtel weather station (Neuchâtel), and the interpolating percentage from the Chaumont weather station (Chaumont).

| **Site** | **Elevation** | **Dist 1** | **Dist 2** | **Neuchâtel** | **Chaumont** |
| --- | --- | --- | --- | --- | --- |
| Top | 1073 | 620 | 651 | 4.80% | 95.20% |
| High | 900 | 447 | 651 | 31.30% | 68.70% |
| Medium | 740 | 287 | 651 | 55.90% | 44.10% |
| Low | 620 | 167 | 651 | 74.30% | 25.70% |

# SECTION 4 – Full statistical analysis of the cumulative nymph abundance (CND) for the four elevation sites

**Background:** In the main manuscript, we restricted the analysis to the cumulative nymph density (CND) at the low, medium, and high elevation sites. For simplicity, we did not include analyses of the cumulative adult tick abundance (CAD). We excluded the top site from the analysis of the CND because we believe that the construction of an adventure park caused the top site to have very different tick population dynamics over time compared to the other three elevation sites. In this section, we analyze the CND for all four sites. With respect to the CND, we show that the most important results remain the same. These results are that the CND increased over time at the three lower elevations, that CND is positive related to beech seed production two years prior, and that it is negatively related with elevation and relative humidity.

**Methods:** We used a model selection approach based on the Akaike information criterion (AIC) to find the most parsimonious model. Models were ranked according to their AIC values and the Akaike weights were calculated for each model. We used the Akaike weights to calculate the model-averaged parameter estimates and their 95% confidence intervals (CIs). The support for a given explanatory variable of interest was calculated as the sum of the Akaike weights of all the models in the set that included that particular explanatory variable. The support for a given explanatory variable ranged from low (0.0%) to high (100%).

**Results – Variation in CND among elevation sites:** We used a one-way ANOVA to compare the mean CND between the four elevations (i.e. this analysis ignores year). The CND was significantly different between the four elevation sites (F_4, 52_ = 5180.0, p < 0.001; Figure S7). The mean CND (and 95% CI) for the low, medium, high, and top elevations were as follows: 21311 (95% CI = 16465–27584), 18127 (95% CI = 14005–23463), 10854 (95% CI =8385–14048), 2421 (95% CI = 1871–3134). In summary, the mean nymph density was inversely related to the altitudinal gradient and it was highest at the low elevation and lowest at the top elevation (Figure S7).

**Results – Effects of the abiotic and biotic variables on the variation in the annual estimates of cumulative nymphal density (CND):** For the CND, the best six models had a combined support of 95.0% (Table S8). The other 46 models had a combined support of 5% (Table S8). The best six models all contained the explanatory variables of elevation site, year, the site:year interaction, and beech tree mast score, but they differed with respect to the identity of the climate variable. The best model had 57.0% of the support, explained 91.6% of the variation in the CND, and contained the explanatory variables of elevation site (partial r^2^ = 65.6%), year (partial r^2^ = 9.1%), the site:year interaction (partial r^2^ = 8.8%), beech tree mast score from 2 years prior (partial r^2^ = 6.9%), and the field-collected relative humidity from the same year (partial r^2^ = 1.4%) (Table S8). For the individual explanatory variables, there was strong support for the explanatory variables of site (100.0%), year (100.0%), site:year interaction (100.0%), beech tree mast score from 2 years prior (100.0%), and moderate support for the field-collected relative humidity from the same year (56.7%), the field-collected saturation deficit from the same year (22.8%; Table S9), and the field-collected temperature from the same year (9.6%; Table S9). None of the other explanatory variables had a support > 2.0% (Table S9).

We calculated the model-averaged parameter estimates to make robust inferences about the relationships between the explanatory variables and the CND (Table S10). We calculated the effect sizes with respect to the following baseline: the site was low elevation, the year was 2004, the beech tree mast was 1, and the field-collected relative humidity from the same year was 50.0%.

The CND was different between the four elevation sites, but this difference was not significant (Figures S7 and S8). The CND at the low elevation was 1.3% higher than the medium elevation (Medium – Low contrast = 0.001, 95% CI = -0.223 – 0.224; Table S10). The CND at the low elevation was 31.5% higher than the high elevation (High – Low = -0.153, 95% CI = -0.383 – 0.076; Table S10). The CND at the low elevation was 49.0% higher than the top elevation (Top – Low = -0.216, 95% CI = -0.460 – 0.028; Table S10). In summary, the CND was inversely related to altitude; it was highest at the low elevation and lowest at the top elevation (Figure S7 and Figure S8).

The site:year interaction indicated that the change in the CND over time differed between the four elevation sites (Figures S7 and S8, Table S10). Over the 15-year period (2004 – 2018), the CND increased by 123.1% at the low site (slope = 0.025, 95% CI = 0.008 – 0.042), increased by 82.8% at the medium site (Medium – Low contrast of the slope = -0.006, 95% CI = -0.030 – 0.018), increased by 55.3% at the high site (High – Low contrast of the slope = -0.011, 95% CI = -0.035 – 0.012), and decreased by 80.8% at the top site (Top – Low contrast of the slope = -0.076, 95% CI = -0.100 – -0.052). In summary, the CND increased over time at the low, medium, and high elevations but decreased at the top elevation (Figures S7 and S8, Table S10).

The covariate beech mast score 2 years prior had a strong positive effect on the CND (0.059 per class, 95% CI = 0.039 – 0.079; Figure S7, Table S10). Increasing the beech mast score from 1 (poor mast) to 5 (full mast) increased the CND by 72.5% at each of the four elevation sites on Chaumont Mountain (Figure S8).

Over the 15-year study period, beech trees produced 6 years of good or full mast scores (2004, 2006, 2009, 2011, 2014, and 2016), where high CND years were expected to occur two years later (2006, 2008, 2011, 2013, 2016, and 2018, respectively). At the low, medium, high, and top site, these 6 good mast years produced 3 (2016, 2018, and 2006), 4 (2006, 2013, 2018, and 2016), 4 (2018, 2006, 2016, and 2011), and 2 (2006 and 2011) of the 6 highest CND values two years later (Figure S7).

The slope of the field-collected relative humidity from the same year was negative (-0.057 per standard deviation; Table S10) and the 95% CI did not overlap zero (-0.096 – -0.019), indicating that the CND decreased with the field-collected relative humidity in the same year. Increasing the field-collected relative humidity from 50.0% to 75.0% decreased the CND from the same year by 38.2% at each of the four elevation sites on Chaumont Mountain (Figure S8; Table S10). Temperature had a positive effect on the CND (slope = 0.059 per standard deviation, 95% CI = 0.009 – 0.109; Table S10) and saturation deficit had a positive effect on the CND (slope = 0.063 per standard deviation, 95% CI = 0.016 – 0.111; Table S10). In summary, our study found that years with higher temperatures and lower relative humidity have higher annual estimates of the CND compared to years with lower temperatures and high relative humidity. According to our model selection table, the level of support for relative humidity (56.7% in Table S9) is 5.9 times higher than that of temperature (9.6% in Table S9). As expected, the saturation deficit has an intermediate level of support (22.8% in Table S9) because it is calculated using both the temperature and relative humidity.

In summary, the CND increased significantly over time at the three lower elevations and decreased at the top elevation. The CND increased with beech tree seed production two years earlier while it decreased significantly with the field-collected relative humidity in the same year (Table S10).

**Discussion – removal of the top elevation in the main manuscript:** We expected that tick abundance would increase more dramatically at the higher elevation sites. In contrast, we found that the CND actually decreased by 81.0% over time at the top elevation. One potential explanation is the construction of recreation facilities near the top of Chaumont Mountain with negative consequences for tick ecology (especially at our top site). Construction of a network of mountain bike trails occurred from 2006 to 2010 and construction of an adventure park, which includes a zip line and outdoor laser games, started in 2011. These recreation facilities have greatly increased the number of human visitors to the top of Chaumont Mountain [1, 2]. The top elevation site was the most affected because it is closest to the adventure park and the start of the mountain biking trails (a distance of 25 m), whereas the other elevation sites are much further away (770, 1500, and 2600 m, respectively; Figure S9). The alteration of the forest caused by the construction and maintenance of these recreational areas since the early 2000’s may have reduced the habitat suitability for ticks. Habitat modifications such as clearing bushes along forest trails have been shown to reduce the abundance of ticks [3]. A larger human footprint may also have reduced the abundance of vertebrate hosts by scaring them away. In summary, a plausible explanation for the 81.0% decrease in nymph abundance at the top elevation site on Chaumont Mountain is that the construction of the outdoor adventure park reduced the habitat quality for ticks and their vertebrate hosts. An interesting alternative explanation is that our monthly tick sampling over a period of 15 years decreased the nymphal tick abundance at the top site. Field studies typically assume that dragging removes a small fraction of the tick population, but this assumption may not be true in habitats where tick density is already low. For this reason, we restricted our analysis in the main manuscript to the three lower elevation sites.

Table S8. Model selection results are shown for the linear models with normal errors of the log10-transformed CND response variable. The explanatory variables were elevation site, year, tree masting variables obtained from MASTREE, and the climate variables obtained from the Climap-net and collected in the field. Shown for each model are the model rank (Rank), model structure (see below for explanation of explanatory variables), model degrees of freedom (Df), log-likelihood (logLik), Akaike information criterion (AIC), difference in the AIC value from the top model (ΔAIC), model weight (Weight1), cumulative weight (Weight2), and adjusted r-squared (r^2^).

| **Rank** | **Model structure** | **Df** | **logLik** | **AIC** | **ΔAIC** | **Weight1** | **Weight2** | **r^2^** |
| --- | --- | --- | --- | --- | --- | --- | --- | --- |
| 1 | CND ~ S+Y+S:Y+B+RH2 | 11 | 43.2 | -58.3 | 0.0 | 57.0 | 57.0 | 91.6 |
| 2 | CND ~ S+Y+S:Y+B+SD2 | 11 | 42.3 | -56.5 | 1.8 | 23.0 | 80.0 | 91.4 |
| 3 | CND ~ S+Y+S:Y+B+T2 | 11 | 41.4 | -54.8 | 3.5 | 10.0 | 90.0 | 91.1 |
| 4 | CND ~ S+Y+S:Y+B+SD2_y-1_ | 11 | 39.8 | -51.5 | 6.8 | 2.0 | 92.0 | 90.6 |
| 5 | CND ~ S+Y+S:Y+B | 10 | 38.2 | -51.5 | 6.8 | 2.0 | 94.0 | 90.2 |
| 6 | CND ~ S+Y+S:Y+B+RH2_y-1_ | 11 | 39.4 | -50.9 | 7.5 | 1.0 | 95.0 | 90.5 |
| 7 | CND ~ S+Y+S:Y+B+T1 | 11 | 39.2 | -50.5 | 7.9 | 1.0 | 96.0 | 90.4 |
| 8 | CND ~ S+Y+S:Y+B+PR | 11 | 39.1 | -50.2 | 8.1 | 1.0 | 97.0 | 90.3 |
| 9 | CND ~ S+Y+S:Y+B+PR_y-1_ | 11 | 38.6 | -49.2 | 9.2 | 1.0 | 98.0 | 90.2 |
| 10 | CND ~ S+Y+S:Y+B+SD1 | 11 | 38.5 | -48.9 | 9.4 | 1.0 | 99.0 | 90.1 |
| 11 | CND ~ S+Y+S:B+B+S:Y | 13 | 41.8 | -48.9 | 9.5 | 1.0 | 100.0 | 90.8 |
| 12 | CND ~ S+Y+S:Y+B+T2_y-1_ | 11 | 38.4 | -48.7 | 9.6 | 0.0 | 100.0 | 90.1 |
| 13 | CND ~ S+Y+S:Y+B+SD1_y-1_ | 11 | 38.3 | -48.6 | 9.7 | 0.0 | 100.0 | 90.1 |
| 14 | CND ~ S+Y+S:Y+B+RH1_y-1_ | 11 | 38.3 | -48.5 | 9.8 | 0.0 | 100.0 | 90.0 |
| 15 | CND ~ S+Y+S:Y+B+T1_y-1_ | 11 | 38.2 | -48.4 | 9.9 | 0.0 | 100.0 | 90.0 |
| 16 | CND ~ S+Y+S:Y+B+RH1 | 11 | 38.2 | -48.4 | 9.9 | 0.0 | 100.0 | 90.0 |
| 17 | CND ~ S+B+T1_y-1_+S:T1_y-1_ | 10 | 27.4 | -29.9 | 28.5 | 0.0 | 100.0 | 85.6 |
| 18 | CND ~ S+B+T1+S:T1 | 10 | 27.3 | -29.7 | 28.7 | 0.0 | 100.0 | 85.6 |
| 19 | CND ~ S+B | 6 | 19.3 | -24.9 | 33.4 | 0.0 | 100.0 | 82.3 |
| 20 | CND ~ S+Y+S:Y | 9 | 22.6 | -23.3 | 35.1 | 0.0 | 100.0 | 83.3 |
| 21 | CND ~ S+B+SD1+S:SD1 | 10 | 24.0 | -23.2 | 35.1 | 0.0 | 100.0 | 83.8 |
| 22 | CND ~ S+Y+B+RH2 | 8 | 20.7 | -22.4 | 35.9 | 0.0 | 100.0 | 82.5 |
| 23 | CND ~ S+Y+B | 7 | 19.3 | -22.3 | 36.0 | 0.0 | 100.0 | 82.0 |
| 24 | CND ~ S+B+SD1_y-1_+S:SD1_y-1_ | 10 | 23.5 | -22.1 | 36.2 | 0.0 | 100.0 | 83.5 |
| 25 | CND ~ S+Y+B+SD2 | 8 | 20.5 | -21.9 | 36.4 | 0.0 | 100.0 | 82.4 |
| 26 | CND ~ S+Y+B+T2 | 8 | 20.3 | -21.5 | 36.8 | 0.0 | 100.0 | 82.3 |
| 27 | CND ~ S+Y+B+PR | 8 | 20.1 | -21.1 | 37.3 | 0.0 | 100.0 | 82.1 |
| 28 | CND ~ S+B+RH2+S:RH2 | 10 | 22.8 | -20.6 | 37.7 | 0.0 | 100.0 | 83.1 |
| 29 | CND ~ S+Y+B+T1 | 8 | 19.7 | -20.4 | 37.9 | 0.0 | 100.0 | 81.9 |
| 30 | CND ~ S+B+S:B | 9 | 21.2 | -20.4 | 37.9 | 0.0 | 100.0 | 82.4 |
| 31 | CND ~ S+Y+B+RH1_y-1_ | 8 | 19.6 | -20.2 | 38.1 | 0.0 | 100.0 | 81.8 |
| 32 | CND ~ S+Y+B+RH2_y-1_ | 8 | 19.6 | -20.1 | 38.2 | 0.0 | 100.0 | 81.8 |
| 33 | CND ~ S+Y+B+SD2_y-1_ | 8 | 19.6 | -20.1 | 38.2 | 0.0 | 100.0 | 81.8 |
| 34 | CND ~ S+Y+B+SD1 | 8 | 19.5 | -20.0 | 38.4 | 0.0 | 100.0 | 81.7 |
| 35 | CND ~ S+Y+B+PR_y-1_ | 8 | 19.4 | -19.7 | 38.6 | 0.0 | 100.0 | 81.7 |
| 36 | CND ~ S+Y+B+T2_y-1_ | 8 | 19.4 | -19.7 | 38.6 | 0.0 | 100.0 | 81.7 |
| 37 | CND ~ S+Y+B+T1_y-1_ | 8 | 19.4 | -19.7 | 38.7 | 0.0 | 100.0 | 81.7 |
| 38 | CND ~ S+Y+B+SD1_y-1_ | 8 | 19.3 | -19.6 | 38.7 | 0.0 | 100.0 | 81.6 |
| 39 | CND ~ S+Y+B+RH1 | 8 | 19.3 | -19.6 | 38.8 | 0.0 | 100.0 | 81.6 |
| 40 | CND ~ S+B+SD2+S:SD2 | 10 | 21.9 | -19.0 | 39.4 | 0.0 | 100.0 | 82.5 |
| 41 | CND ~ S+B+PR_y-1_+S:PR_y-1_ | 10 | 21.7 | -18.5 | 39.9 | 0.0 | 100.0 | 82.4 |
| 42 | CND ~ S+B+T2+S:T2 | 10 | 21.6 | -18.2 | 40.1 | 0.0 | 100.0 | 82.3 |
| 43 | CND ~ S+Y+B+S:B | 10 | 21.2 | -17.5 | 40.9 | 0.0 | 100.0 | 82.1 |
| 44 | CND ~ S+B+PR+S:PR | 10 | 21.1 | -17.4 | 41.0 | 0.0 | 100.0 | 82.0 |
| 45 | CND ~ S+B+RH1_y-1_+S:RH1_y-1_ | 10 | 20.4 | -15.8 | 42.5 | 0.0 | 100.0 | 81.5 |
| 46 | CND ~ S+B+RH2_y-1_+S:RH2_y-1_ | 10 | 20.2 | -15.5 | 42.8 | 0.0 | 100.0 | 81.4 |
| 47 | CND ~ S+B+RH1+S:RH1 | 10 | 20.1 | -15.4 | 43.0 | 0.0 | 100.0 | 81.4 |
| 48 | CND ~ S+B+SD2_y-1_+S:SD2_y-1_ | 10 | 19.8 | -14.7 | 43.6 | 0.0 | 100.0 | 81.2 |
| 49 | CND ~ S+B+T2_y-1_+S:T2_y-1_ | 10 | 19.4 | -14.0 | 44.4 | 0.0 | 100.0 | 80.9 |
| 50 | CND ~ S+Y | 6 | 10.3 | -6.9 | 51.5 | 0.0 | 100.0 | 75.6 |
| 51 | CND ~ B | 3 | -29.5 | 65.6 | 123.9 | 0.0 | 100.0 | 4.5 |
| 52 | CND ~ P | 3 | -31.0 | 68.6 | 126.9 | 0.0 | 100.0 | 0.0 |

The acronyms for the explanatory variables are as follows: S = site, Y = year, B = beech tree mast score, P = spruce tree mast score, T1 = temperature from the Climap-net data, T1_y-1_ = temperature in year y-1 from the Climap-net data, RH1 = relative humidity from the Climap-net data, RH1_y-1_ = relative humidity in year y-1 from the Climap-net data, SD1 = saturation deficit from the Climap-net data, SD1_y-1_ = saturation deficit in year y-1 from the Climap-net data, PR = precipitation from the Climap-net data, and PR_y-1_ = precipitation in year y-1 from the Climap-net data, T2 = temperature from the field-collected data, T2_y-1_ = temperature in year y-1 from the field-collected data, RH2 = relative humidity from the field-collected data, RH2_y-1_ = relative humidity in year y-1 from the field-collected data, SD2 = saturation deficit from the field-collected data, SD2_y-1_ = saturation deficit in year y-1 from the field-collected data.

Table S9. The support for each individual explanatory variable is shown for the CND. This support is calculated as the sum of the Akaike weights for all the models in the set that include that particular explanatory variable.

| **Rank** | **Explanatory variable of interest** | **Support (%)** |
| --- | --- | --- |
| 1 | Site | 100.0 |
| 2 | Beech tree mast score | 100.0 |
| 3 | Year | 100.0 |
| 4 | Site:Year | 100.0 |
| 5 | RH2 | 56.7 |
| 6 | SD2 | 22.8 |
| 7 | T2 | 9.6 |
| 8 | SD2y-1 | 1.9 |
| 9 | RH2y-1 | 1.4 |
| 10 | T1 | 1.1 |
| 11 | PR | 1.0 |
| 12 | PRy-1 | < 1.0 |
| 13 | SD1 | < 1.0 |
| 14 | T2y-1 | < 1.0 |
| 15 | SD1y-1 | < 1.0 |
| 16 | RH1y-1 | < 1.0 |
| 17 | T1y-1 | < 1.0 |
| 18 | RH1 | < 1.0 |
| 19 | Site:T1y-1 | < 1.0 |
| 20 | Site:T1 | < 1.0 |
| 21 | Site:SD1 | < 1.0 |
| 22 | Site:SD1y-1 | < 1.0 |
| 23 | Site:RH2 | < 1.0 |
| 24 | Site:SD2 | < 1.0 |
| 25 | Site:PRy-1 | < 1.0 |
| 26 | Site:T2 | < 1.0 |
| 27 | Site:PR | < 1.0 |
| 28 | Site:RH1y-1 | < 1.0 |
| 29 | Site:RH2y-1 | < 1.0 |
| 30 | Site:RH1 | < 1.0 |
| 31 | Site:SD2y-1 | < 1.0 |
| 32 | Site:T2y-1 | < 1.0 |
| 33 | Spruce tree mast score | < 1.0 |
| 34 | Site:Beech tree mast score | < 1.0 |

The acronyms for the explanatory variables are as follows: T1 = temperature from the Climap-net data, T1_y-1_ = temperature in year y-1 from the Climap-net data, RH1 = relative humidity from the Climap-net data, RH1_y-1_ = relative humidity in year y-1 from the Climap-net data, SD1 = saturation deficit from the Climap-net data, SD1_y-1_ = saturation deficit in year y-1 from the Climap-net data, PR = precipitation from the Climap-net data, and PR_y-1_ = precipitation in year y-1 from the Climap-net data, T2 = temperature from the field-collected data, T2_y-1_ = temperature in year y-1 from the field-collected data, RH2 = relative humidity from the field-collected data, RH2_y-1_ = relative humidity in year y-1 from the field-collected data, SD2 = saturation deficit from the field-collected data, SD2_y-1_ = saturation deficit in year y-1 from the field-collected data.

Table S10. Model-averaged parameter estimates are shown for the linear models of the log10-transformed CND response variable. Shown are the parameter types, the parameter names, the parameter estimates, and the 95% confidence limits (LL = lower limit and UL = upper limit). Estimate 1 is averaged over all the models in the set. Estimate 2 is averaged over the subset of models with a cumulative support of 95%. The 95% confidence limits are for estimate 2. Statistically significant parameter estimates are shown in bold-face type.

| **Type** | **Name** | **Estimate 1** | **Estimate 2** | **95% LL** | **95% UL** |
| --- | --- | --- | --- | --- | --- |
| Intercept | Low site | 3.919 | 3.919 | 3.747 | 4.090 |
| Contrast 1 | Medium site | 0.001 | 0.001 | -0.223 | 0.224 |
| Contrast 2 | High site | -0.153 | -0.153 | -0.383 | 0.076 |
| Contrast 3 | Top site | -0.216 | -0.216 | -0.460 | 0.028 |
| **Slope 1** | **Year** | **0.025** | **0.025** | **0.008** | **0.042** |
| **Slope 2** | **Beech tree mast score** | **0.059** | **0.059** | **0.039** | **0.079** |
| Slope 3 | Spruce tree mast score | 0.000 | 0.034 | -0.055 | 0.124 |
| Slope 4 | T1 | 0.001 | 0.054 | -0.029 | 0.138 |
| Slope 5 | RH1 | 0.000 | -0.003 | -0.051 | 0.045 |
| Slope 6 | SD1 | 0.000 | 0.021 | -0.043 | 0.085 |
| Slope 7 | PR | 0.000 | 0.025 | -0.016 | 0.065 |
| Slope 8 | T1_y-1_ | 0.000 | -0.008 | -0.095 | 0.079 |
| Slope 9 | RH1_y-1_ | 0.000 | 0.009 | -0.051 | 0.068 |
| Slope 10 | SD1_y-1_ | 0.000 | 0.017 | -0.058 | 0.092 |
| Slope 11 | PR_y-1_ | 0.000 | -0.017 | -0.061 | 0.026 |
| **Slope 12** | **T2** | **0.006** | **0.059** | **0.009** | **0.109** |
| **Slope 13** | **RH2** | **-0.033** | **-0.057** | **-0.096** | **-0.019** |
| **Slope 14** | **SD2** | **0.014** | **0.063** | **0.016** | **0.111** |
| Slope 15 | T2_y-1_ | 0.000 | 0.014 | -0.041 | 0.069 |
| Slope 16 | RH2_y-1_ | 0.000 | -0.032 | -0.077 | 0.013 |
| Slope 17 | SD2_y-1_ | 0.001 | 0.045 | -0.011 | 0.101 |
| Contrast 4 | Medium site:Year | -0.006 | -0.006 | -0.030 | 0.018 |
| Contrast 5 | High site:Year | -0.011 | -0.011 | -0.035 | 0.012 |
| **Contrast 6** | **Top site:Year** | **-0.076** | **-0.076** | **-0.100** | **-0.052** |
| Contrast 7 | Medium site:Beech mast score | 0.000 | 0.016 | -0.041 | 0.073 |
| Contrast 8 | High site: Beech mast score | 0.000 | 0.053 | -0.004 | 0.110 |
| Contrast 9 | Top site: Beech mast score | 0.000 | -0.012 | -0.068 | 0.045 |
| Contrast 10 | Medium site:T1 | 0.000 | -0.079 | -0.322 | 0.163 |
| Contrast 11 | High site:T1 | 0.000 | -0.072 | -0.310 | 0.166 |
| Contrast 12 | Top site:T1 | 0.000 | -0.395 | -0.628 | -0.162 |
| Contrast 13 | Medium site:RH1 | 0.000 | 0.074 | -0.112 | 0.261 |
| Contrast 14 | High site:RH1 | 0.000 | 0.002 | -0.183 | 0.188 |
| Contrast 15 | Top site:RH1 | 0.000 | 0.080 | -0.100 | 0.260 |
| Contrast 16 | Medium site:SD1 | 0.000 | -0.065 | -0.253 | 0.124 |
| Contrast 17 | High site:SD1 | 0.000 | 0.005 | -0.192 | 0.201 |
| Contrast 18 | Top site:SD1 | 0.000 | -0.264 | -0.469 | -0.060 |
| Contrast 19 | Medium site:PR | 0.000 | 0.050 | -0.095 | 0.195 |
| Contrast 20 | High site:PR | 0.000 | -0.017 | -0.163 | 0.128 |
| Contrast 21 | Top site:PR | 0.000 | 0.071 | -0.073 | 0.214 |
| Contrast 22 | Medium site:T1_y-1_ | 0.000 | -0.136 | -0.395 | 0.122 |
| Contrast 23 | High site:T1_y-1_ | 0.000 | -0.178 | -0.431 | 0.074 |
| Contrast 24 | Top site:T1_y-1_ | 0.000 | -0.458 | -0.705 | -0.212 |
| Contrast 25 | Medium site:RH1_y-1_ | 0.000 | 0.014 | -0.194 | 0.222 |
| Contrast 26 | High site:RH1_y-1_ | 0.000 | -0.040 | -0.246 | 0.166 |
| Contrast 27 | Top site:RH1_y-1_ | 0.000 | 0.067 | -0.132 | 0.266 |
| Contrast 28 | Medium site:SD1_y-1_ | 0.000 | -0.038 | -0.264 | 0.187 |
| Contrast 29 | High site:SD1_y-1_ | 0.000 | -0.008 | -0.241 | 0.224 |
| Contrast 30 | Top site:SD1_y-1_ | 0.000 | -0.290 | -0.528 | -0.052 |
| Contrast 31 | Medium site:PR_y-1_ | 0.000 | -0.001 | -0.150 | 0.147 |
| Contrast 32 | High site:PR_y-1_ | 0.000 | -0.002 | -0.151 | 0.146 |
| Contrast 33 | Top site:PR_y-1_ | 0.000 | 0.118 | -0.029 | 0.265 |
| Contrast 34 | Medium site:T2 | 0.000 | -0.060 | -0.274 | 0.154 |
| Contrast 35 | High site:T2 | 0.000 | 0.050 | -0.162 | 0.262 |
| Contrast 36 | Top site:T2 | 0.000 | -0.073 | -0.285 | 0.138 |
| Contrast 37 | Medium site:RH2 | 0.000 | -0.052 | -0.198 | 0.094 |
| Contrast 38 | High site:RH2 | 0.000 | -0.104 | -0.262 | 0.053 |
| Contrast 39 | Top site:RH2 | 0.000 | 0.038 | -0.123 | 0.199 |
| Contrast 40 | Medium site:SD2 | 0.000 | 0.046 | -0.117 | 0.208 |
| Contrast 41 | High site:SD2 | 0.000 | 0.145 | -0.039 | 0.330 |
| Contrast 42 | Top site:SD2 | 0.000 | 0.041 | -0.175 | 0.256 |
| Contrast 43 | Medium site:T2_y-1_ | 0.000 | 0.014 | -0.215 | 0.242 |
| Contrast 44 | High site:T2_y-1_ | 0.000 | 0.014 | -0.213 | 0.241 |
| Contrast 45 | Top site:T2_y-1_ | 0.000 | -0.008 | -0.235 | 0.219 |
| Contrast 46 | Medium site:RH2_y-1_ | 0.000 | 0.016 | -0.136 | 0.168 |
| Contrast 47 | High site:RH2_y-1_ | 0.000 | -0.056 | -0.219 | 0.107 |
| Contrast 48 | Top site:RH2_y-1_ | 0.000 | 0.021 | -0.144 | 0.185 |
| Contrast 49 | Medium site:SD2_y-1_ | 0.000 | 0.004 | -0.167 | 0.174 |
| Contrast 50 | High site:SD2_y-1_ | 0.000 | 0.053 | -0.138 | 0.243 |
| Contrast 51 | Top site:SD2_y-1_ | 0.000 | 0.044 | -0.172 | 0.261 |

The acronyms for the explanatory variables are as follows: T1 = temperature from the Climap-net data, T1_y-1_ = temperature in year y-1 from the Climap-net data, RH1 = relative humidity from the Climap-net data, RH1_y-1_ = relative humidity in year y-1 from the Climap-net data, SD1 = saturation deficit from the Climap-net data, SD1_y-1_ = saturation deficit in year y-1 from the Climap-net data, PR = precipitation from the Climap-net data, and PR_y-1_ = precipitation in year y-1 from the Climap-net data, T2 = temperature from the field-collected data, T2_y-1_ = temperature in year y-1 from the field-collected data, RH2 = relative humidity from the field-collected data, RH2_y-1_ = relative humidity in year y-1 from the field-collected data, SD2 = saturation deficit from the field-collected data, SD2_y-1_ = saturation deficit in year y-1 from the field-collected data.


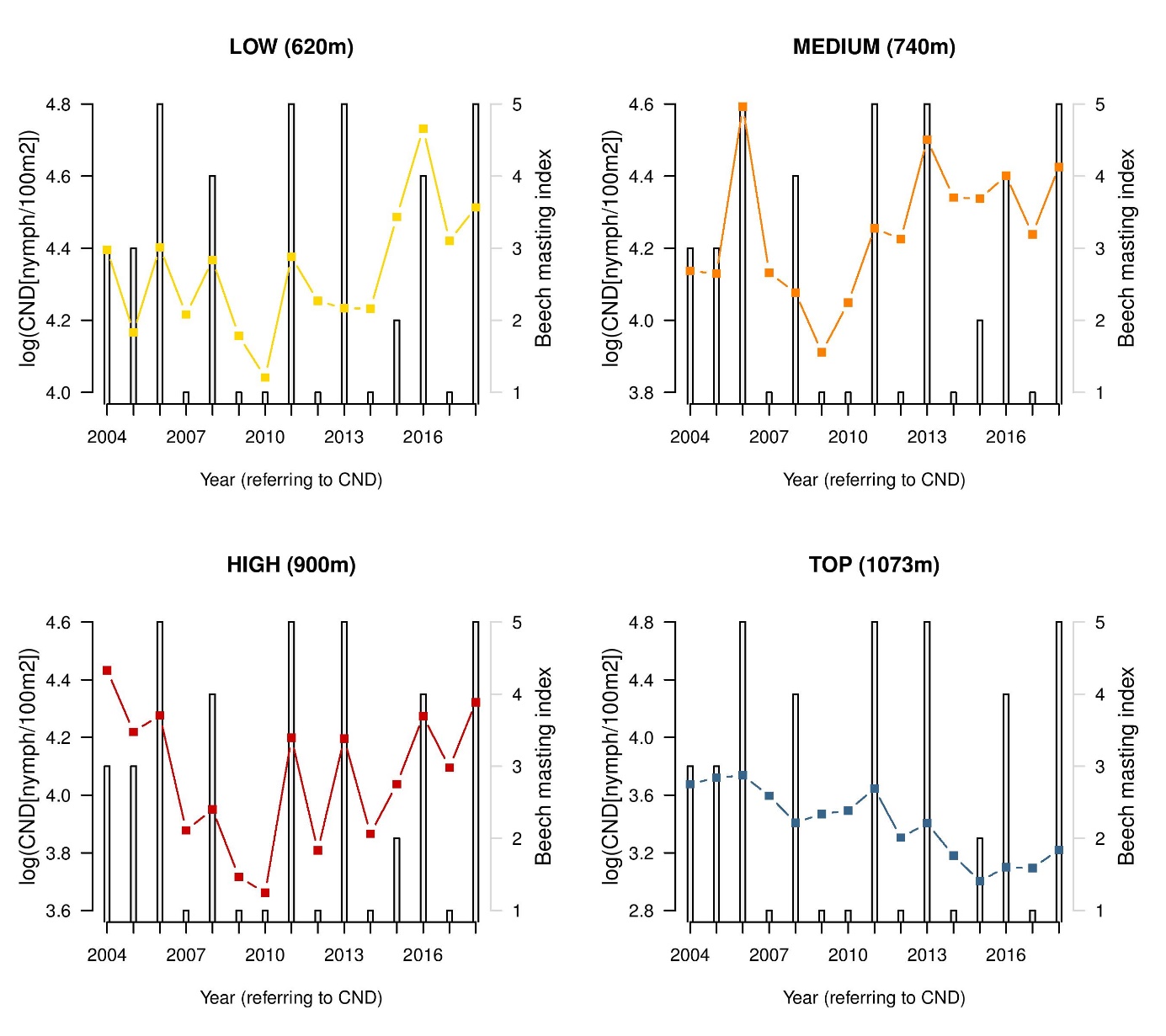
Figure S7. The log10-transformed cumulative nymphal density (CND; points and solid lines) and the beech tree mast score (grey bars) are shown over time for each of the four elevations on Chaumont Mountain. The CND increased significantly over the 15-year study period (2004–2018) at the low, medium, and high elevation sites, but it decreased significantly at the top elevation. Years of high seed production by beech trees cause high CND two years later. The CND is the total number of questing *I. ricinus* nymphs sampled by the dragging method each year. Beech tree mast score ranges from 1 to 5 (class 1 = very poor, 2 = poor, 3 = moderate, 4 = good, and class 5 = full mast year).


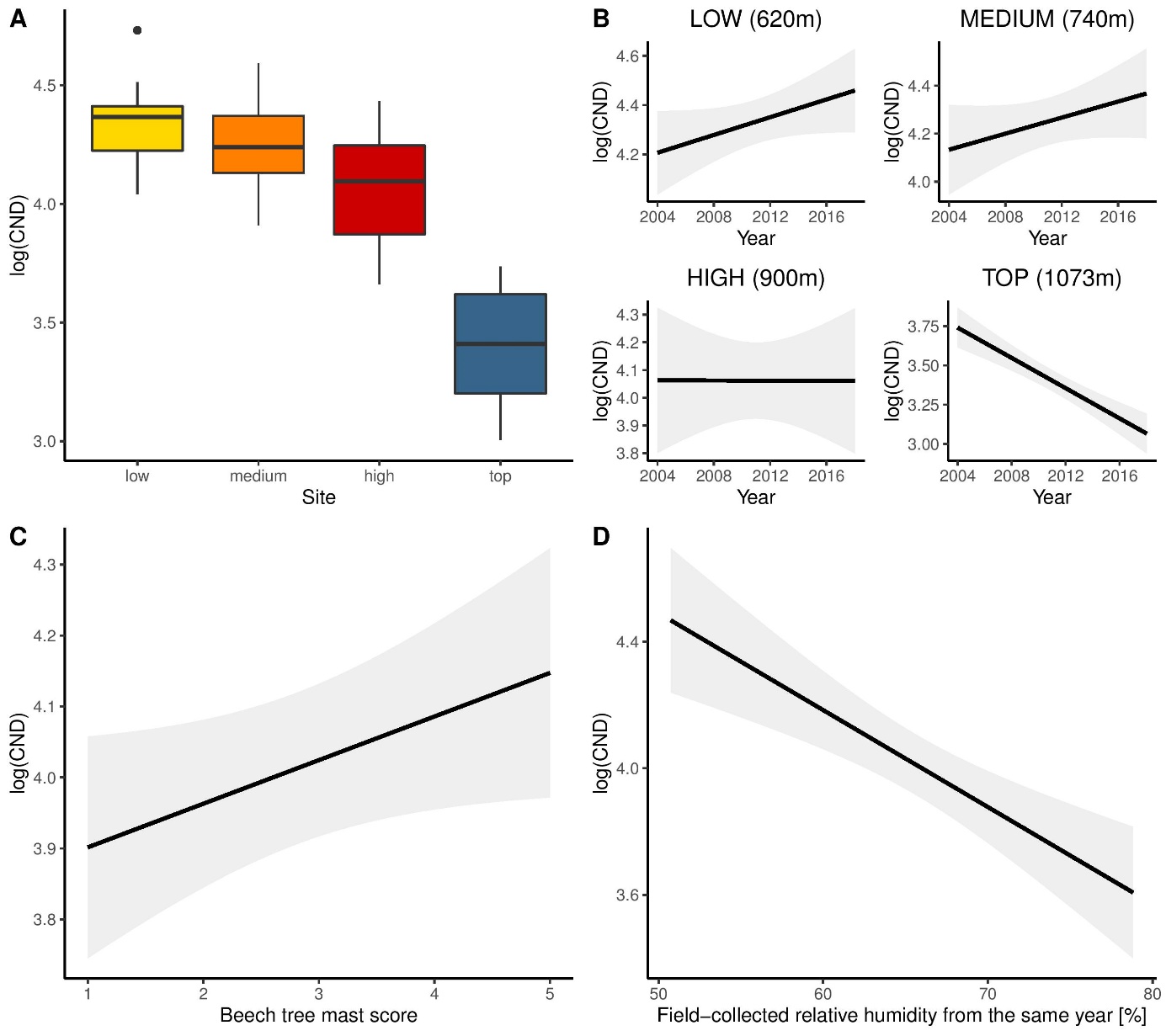


Figure S8. Effect sizes of the explanatory variables (elevation, year, site:year interaction, beech mast score 2 years prior, and mean annual relative humidity in the same year) on the log10-transformed cumulative nymphal density (CND). The parameter estimates used to calculate the effect sizes were taken from the model-averaged in Table S10 (A) Effect of elevation on the CND. The CND at the low elevation was 1.3% higher than the medium elevation, 31.5% higher than the high elevation, and 49.0% higher than the top elevation (partial r^2^ = 65.6%). (B) Effect of year (e.g. time) on the CND. The CND increased by 123.1% at the low elevation, 82.8% at the medium elevation, and 55.3% at the high elevation, but decreased by 80.8% at the top elevation (partial r^2^ = 8.8%). (C) Effect of beech mast score on the CND. Increasing the beech mast score from 1 (poor mast) to 5 (full mast) increased the CND by 72.5% (partial r^2^ = 6.9%). (D) Effect of the mean annual field-collected relative humidity on the CND in the same year. Increasing the field-collected relative humidity from 50.0% to 75.0% decreased the CND by 38.2% (partial r^2^ = 1.4%).


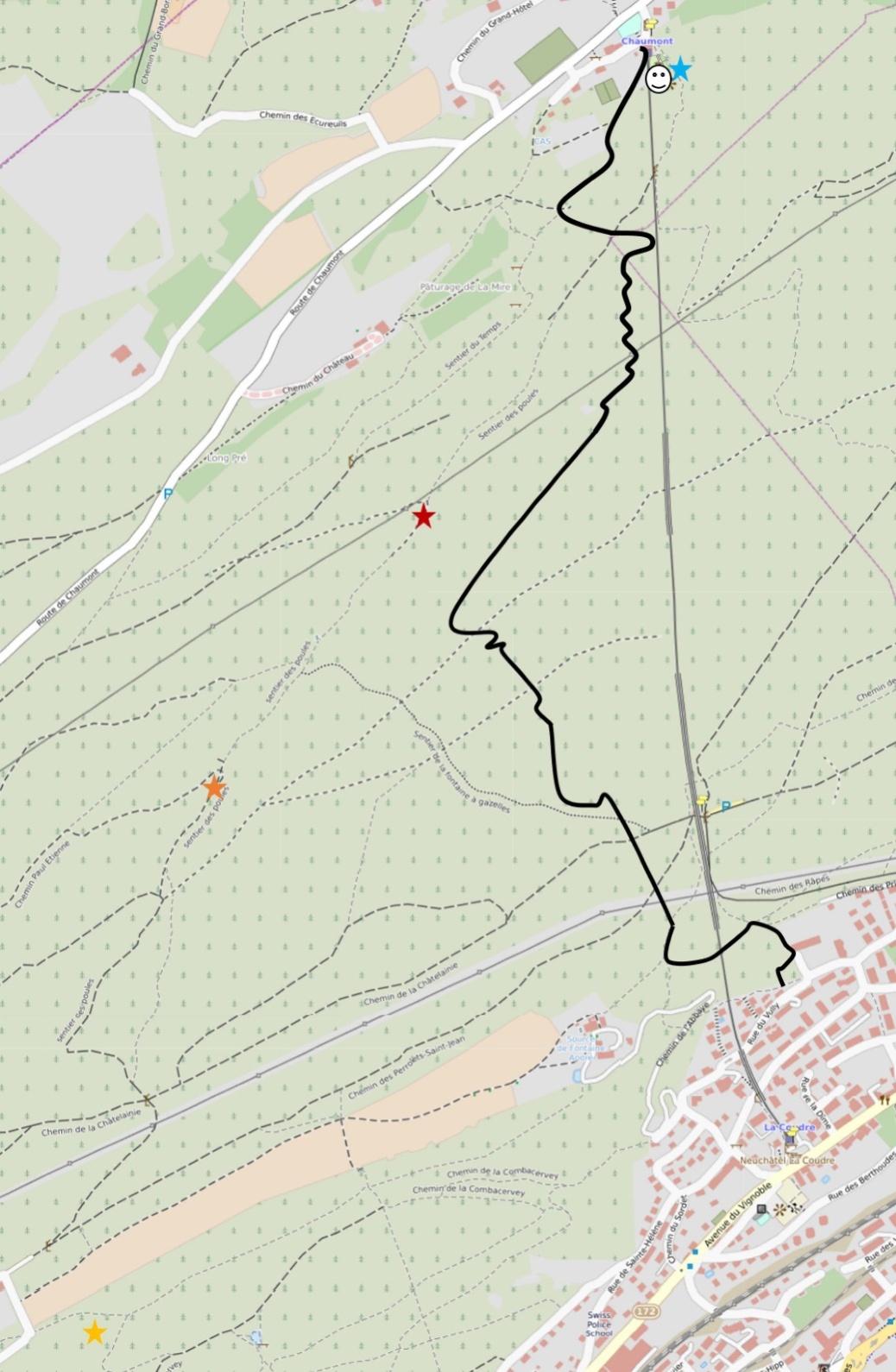


Figure S9. Map of the four elevation sites on Chaumont Mountain, canton Neuchâtel, Switzerland. The stars represent the 4 different elevation sites: low elevation site (yellow star), medium elevation site (orange) star, high elevation site (red star), and top elevation site (blue star). The smiley face indicates the location of the outdoor adventure park, and the solid black line represent the mountain biking trail.

# SECTION 5 – Full statistical analysis of the cumulative adult abundance (CAD) for the four elevation sites

**Background:** In the main manuscript, we restricted the analysis to the cumulative nymph density (CND) at the low, medium, and high elevation sites. For simplicity, we did not include analyses of the cumulative adult tick abundance (CAD). We excluded the top site from the analysis of the CND because we believe that the construction of an adventure park caused the top site to have very different tick population dynamics over time compared to the other three elevation sites. In this section, we analyze the CAD for all four sites.

**Methods:** We used a model selection approach based on the Akaike information criterion (AIC) to find the most parsimonious model. Models were ranked according to their AIC values and the Akaike weights were calculated for each model. We used the Akaike weights to calculate the model-averaged parameter estimates and their 95% confidence intervals (CIs). The support for a given explanatory variable of interest was calculated as the sum of the Akaike weights of all the models in the set that included that particular explanatory variable. The support for a given explanatory variable ranged from low (0.0%) to high (100%).

**Results – Variation in CAD among elevation sites:** We used a one-way ANOVA to compare the mean CAD between the four elevations (i.e. this analysis ignores year). The CAD was significantly different between the four elevation sites (F_4, 52_ = 1785.2, p < 0.001; Figure S10). The mean CAD (and 95% CI) for the low, medium, high, and top elevations were as follows: 2718 (95% CI = 1861–3970), 5689 (95% CI = 3895–8309), 3368 (95% CI = 2306–4919), and 1281 (95% CI =877–1871). In summary, the mean adult density was highest in the medium and high elevations and lowest at the low and top elevations (Figure S10). The ratios of nymphs to adult ticks at the low, medium, high, and top elevations were as follows: 7.84, 3.19, 3.22, and 1.89.

**Results – Effects of the abiotic and biotic variables on the variation in the annual estimates of cumulative adult density (CAD):** For the CAD, the best fourteen models had a combined support of 95.0% (Table S11). The other 38 models had a combined support of 5% (Table S11). The best fourteen models all contained the explanatory variables of elevation site, year, the site:year interaction, and beech tree mast score, but they differed with respect to the identity of the climate variable. The best model had 14.0% of the support, explained 64.6% of the variation in the CAD, and contained the explanatory variables of site (partial r^2^ = 47.7%), year (partial r^2^ = 10.6%), the site:year interaction (partial r^2^ = 9.8%), and beech tree mast score from 2 years prior (partial r^2^ = 21.0%) (Table S11). For the individual explanatory variables, there was strong support for the main effects of site (100.0%), beech tree mast score from 2 years prior (100.0%), year (99.1%), and the site:year interaction (98.2%;Table S12). None of the other explanatory variables had a support > 13.0% (Table S12).

We calculated the model-averaged parameter estimates to make robust inferences about the relationships between the explanatory variables and the CAD (Table S13). We also calculated the effect sizes with respect to the following baseline: the site was low elevation, the year was 2004, and the beech tree mast score was 1.

The CAD was different between the four elevation sites (Figures S10 and S11, Table S13). The CAD at the medium site was 256.6% higher than the low site (Medium – Low contrast = 0.583, 95% CI = 0.177 – 0.990). The CAD at the high site was 224.6% higher than the low site (High – Low contrast = 0.567, 95% CI = 0.134 – 1.001). The CAD at the top site was 108.0% higher than the low site (High – Low contrast = 0.404, 95% CI = -0.094 – 0.903). In summary, the CAD was highest at the medium and high site and lowest at the low and top site (Figures S10 and S11, Table S13).

The site:year interaction indicated that the change in the CAD over time differed between the four elevation sites (Figures S10 and S11, Table S13). Over the 15-year period (2004 – 2018), the CAD increased by 280.3% at the low site (slope = 0.041, 95% CI = 0.010 – 0.073), increased by 38.9% at the medium site (Medium – Low contrast of the slope = -0.031, 95% CI = -0.074 – 0.011), decreased by 37.7% at the high site (High – Low contrast of the slope = -0.056, 95% CI = -0.099 – -0.014), and decreased by 76.5% at the top site (Top – Low contrast of the slope = -0.086, 95% CI = -0.129 – -0.044). In summary, the CAD increased over time at the low and medium elevations but decreased at the high and top elevations (Figures S10 and S11, Table S13).

The covariate beech mast score 3 years prior had a strong positive effect on the CAD (0.100 per class, 95% CI = 0.060– 0.139; Figure S10, Table S13). Increasing the beech mast score from 1 (poor mast) to 5 (full mast) increased the CAD by 150.4% at each of the four elevation sites on Chaumont Mountain (Figure S11).

Over the 15-year study period, beech trees produced 6 years of good or full mast scores (2004, 2006, 2009, 2011, 2014, and 2016), where high CAD years were expected to occur three years later (2007, 2009, 2012, 2014, 2017, and 2019, respectively). At the low, medium, high, and top site, these 5 good mast years (e.g. no CAD data for 2019) produced 4 (2017, 2007, 2014, and 2012), 4 (2017, 2014, 2007, and 2012), 2 (2007 and 2017), and 3 (2007, 2012, and 2014) of the 5 highest CAD values three years later (Figure S10).

In summary, the CAD increased significantly over time at the low and medium elevations but decreased at the high and top elevations. The CAD increased with beech tree seed production three years earlier (Table S13).

Table S11. Model selection results are shown for the linear models with normal errors of the log10-transformed CAD response variable. The explanatory variables were elevation site, year, tree masting variables obtained from MASTREE, and the climate variables obtained from the Climap-net and collected in the field. Shown for each model are the model rank (Rank), model structure (see below for explanation of explanatory variables), model degrees of freedom (Df), log-likelihood (logLik), Akaike information criterion (AIC), difference in the AIC value from the top model (ΔAIC), model weight (Weight1), cumulative weight (Weight2), and adjusted r-squared (r^2^).

| **Rank** | **Model structure** | **Df** | **logLik** | **AIC** | **ΔAIC** | **Weight1** | **Weight2** | **r^2^** |
| --- | --- | --- | --- | --- | --- | --- | --- | --- |
| 1 | CAD ~ S+Y+S:Y+B | 10 | 8.8 | 7.3 | 0.0 | 14.0 | 14.0 | 64.6 |
| 2 | CAD ~ S+Y+S:Y+B+RH2 | 11 | 10.2 | 7.5 | 0.2 | 12.0 | 26.0 | 65.7 |
| 3 | CAD ~ S+Y+S:Y+B+PR_y-1_ | 11 | 10.1 | 7.9 | 0.6 | 10.0 | 36.0 | 65.4 |
| 4 | CAD ~ S+Y+S:Y+B+SD2_y-1_ | 11 | 10.0 | 7.9 | 0.6 | 10.0 | 46.0 | 65.4 |
| 5 | CAD ~ S+Y+S:Y+B+PR | 11 | 10.0 | 8.1 | 0.7 | 9.0 | 55.0 | 65.3 |
| 6 | CAD ~ S+Y+S:Y+B+RH2_y-1_ | 11 | 9.9 | 8.2 | 0.9 | 9.0 | 64.0 | 65.2 |
| 7 | CAD ~ S+Y+S:Y+B+SD2 | 11 | 9.3 | 9.4 | 2.1 | 5.0 | 69.0 | 64.5 |
| 8 | CAD ~ S+Y+S:Y+B+RH1_y-1_ | 11 | 9.3 | 9.5 | 2.2 | 5.0 | 74.0 | 64.4 |
| 9 | CAD ~ S+Y+S:Y+B+T1 | 11 | 9.2 | 9.6 | 2.3 | 4.0 | 78.0 | 64.4 |
| 10 | CAD ~ S+Y+S:Y+B+T2_y-1_ | 11 | 9.1 | 9.7 | 2.4 | 4.0 | 82.0 | 64.3 |
| 11 | CAD ~ S+Y+S:Y+B+SD1 | 11 | 9.0 | 9.9 | 2.6 | 4.0 | 86.0 | 64.2 |
| 12 | CAD ~ S+Y+S:Y+B+T1_y-1_ | 11 | 8.8 | 10.4 | 3.1 | 3.0 | 89.0 | 63.9 |
| 13 | CAD ~ S+Y+S:Y+B+T2 | 11 | 8.8 | 10.4 | 3.1 | 3.0 | 92.0 | 63.8 |
| 14 | CAD ~ S+Y+S:Y+B+SD1_y-1_ | 11 | 8.8 | 10.4 | 3.1 | 3.0 | 95.0 | 63.8 |
| 15 | CAD ~ S+Y+S:Y+B+RH1 | 11 | 8.8 | 10.4 | 3.1 | 3.0 | 98.0 | 63.8 |
| 16 | CAD ~ S+Y+S:Y+B +S:B | 13 | 10.6 | 13.4 | 6.1 | 1.0 | 99.0 | 64.6 |
| 17 | CAD ~ S+B | 6 | -0.4 | 14.5 | 7.1 | 0.0 | 99.0 | 54.8 |
| 18 | CAD ~ S+B+T1+S:T1 | 10 | 4.8 | 15.2 | 7.9 | 0.0 | 99.0 | 59.2 |
| 19 | CAD ~ S+Y+B | 7 | -0.3 | 16.9 | 9.5 | 0.0 | 99.0 | 54.0 |
| 20 | CAD ~ S+Y+B+PR_y-1_ | 8 | 0.9 | 17.2 | 9.9 | 0.0 | 99.0 | 55.1 |
| 21 | CAD ~ S+Y+B+PR | 8 | 0.9 | 17.2 | 9.9 | 0.0 | 99.0 | 55.0 |
| 22 | CAD ~ S+B+T1_y-1_+S:T1_y-1_ | 10 | 3.5 | 17.8 | 10.5 | 0.0 | 99.0 | 57.3 |
| 23 | CAD ~ S+Y+B+RH1_y-1_ | 8 | 0.5 | 18.0 | 10.7 | 0.0 | 99.0 | 54.4 |
| 24 | CAD ~ S+Y+B+RH2 | 8 | 0.5 | 18.2 | 10.8 | 0.0 | 99.0 | 54.3 |
| 25 | CAD ~ S+Y+B+SD2_y-1_ | 8 | 0.3 | 18.5 | 11.2 | 0.0 | 99.0 | 54.0 |
| 26 | CAD ~ S+Y+B+RH2_y-1_ | 8 | 0.2 | 18.6 | 11.3 | 0.0 | 99.0 | 53.9 |
| 27 | CAD ~ S+B+RH2+S:RH2 | 10 | 3.1 | 18.6 | 11.3 | 0.0 | 99.0 | 56.7 |
| 28 | CAD ~ S+Y+B+T1 | 8 | 0.1 | 18.9 | 11.5 | 0.0 | 99.0 | 53.7 |
| 29 | CAD ~ S+Y+B+SD2 | 8 | 0.0 | 19.1 | 11.7 | 0.0 | 99.0 | 53.6 |
| 30 | CAD ~ S+Y+B+T2_y-1_ | 8 | 0.0 | 19.1 | 11.8 | 0.0 | 99.0 | 53.5 |
| 31 | CAD ~ S+B+T2+S:T2 | 10 | 2.8 | 19.2 | 11.9 | 0.0 | 99.0 | 56.2 |
| 32 | CAD ~ S+Y+B+SD1 | 8 | -0.1 | 19.3 | 12.0 | 0.0 | 99.0 | 53.3 |
| 33 | CAD ~ S+Y+B+T1_y-1_ | 8 | -0.2 | 19.5 | 12.2 | 0.0 | 99.0 | 53.2 |
| 34 | CAD ~ S+Y+B+T2 | 8 | -0.2 | 19.5 | 12.2 | 0.0 | 99.0 | 53.2 |
| 35 | CAD ~ S+Y+B+SD1_y-1_ | 8 | -0.3 | 19.6 | 12.3 | 0.0 | 99.0 | 53.1 |
| 36 | CAD ~ S+Y+B+RH1 | 8 | -0.3 | 19.6 | 12.3 | 0.0 | 99.0 | 53.1 |
| 37 | CAD ~ S+B+SD2_y-1_+S:SD2_y-1_ | 10 | 2.6 | 19.7 | 12.4 | 0.0 | 99.0 | 55.9 |
| 38 | CAD ~ S+B+SD1+S:SD1 | 10 | 2.5 | 19.9 | 12.6 | 0.0 | 99.0 | 55.7 |
| 39 | CAD ~ S+B+RH2_y-1_+S:RH2_y-1_ | 10 | 2.4 | 20.2 | 12.9 | 0.0 | 99.0 | 55.5 |
| 40 | CAD ~ S+B+SD2+S:SD2 | 10 | 2.1 | 20.7 | 13.4 | 0.0 | 99.0 | 55.0 |
| 41 | CAD ~ S+B+SD1_y-1_+S:SD1_y-1_ | 10 | 2.1 | 20.7 | 13.4 | 0.0 | 99.0 | 55.0 |
| 42 | CAD ~ S+B+S:B | 9 | 0.3 | 21.3 | 14.0 | 0.0 | 99.0 | 53.1 |
| 43 | CAD ~ S+B+PR_y-1_+S:PR_y-1_ | 10 | 1.3 | 22.2 | 14.9 | 0.0 | 99.0 | 53.8 |
| 44 | CAD ~ S+B+PR+S:PR | 10 | 1.3 | 22.3 | 14.9 | 0.0 | 99.0 | 53.8 |
| 45 | CAD ~ S+B+RH1+S:RH1 | 10 | 0.9 | 23.2 | 15.9 | 0.0 | 99.0 | 53.0 |
| 46 | CAD ~ S+B+RH1_y-1_+S:RH1_y-1_ | 10 | 0.7 | 23.6 | 16.2 | 0.0 | 99.0 | 52.7 |
| 47 | CAD ~ S+Y+B+S:B | 10 | 0.4 | 24.0 | 16.7 | 0.0 | 99.0 | 52.3 |
| 48 | CAD ~ S+B+T2_y-1_+S:T2_y-1_ | 10 | 0.3 | 24.4 | 17.0 | 0.0 | 99.0 | 52.0 |
| 49 | CAD ~ S+Y+S:Y | 9 | -4.6 | 31.2 | 23.8 | 0.0 | 99.0 | 44.1 |
| 50 | CAD ~ S+Y | 6 | -10.6 | 34.8 | 27.5 | 0.0 | 99.0 | 34.9 |
| 51 | CAD ~ B | 3 | -18.5 | 43.5 | 36.2 | 0.0 | 99.0 | 18.3 |
| 52 | CAD ~ P | 3 | -21.8 | 50.1 | 42.7 | 0.0 | 99.0 | 8.2 |

The acronyms for the explanatory variables are as follows: S = site, Y = year, B = beech tree mast score, P = spruce tree mast score, T1 = temperature from the Climap-net data, T1_y-1_ = temperature in year y-1 from the Climap-net data, RH1 = relative humidity from the Climap-net data, RH1_y-1_ = relative humidity in year y-1 from the Climap-net data, SD1 = saturation deficit from the Climap-net data, SD1_y-1_ = saturation deficit in year y-1 from the Climap-net data, PR = precipitation from the Climap-net data, and PR_y-1_ = precipitation in year y-1 from the Climap-net data, T2 = temperature from the field-collected data, T2_y-1_ = temperature in year y-1 from the field-collected data, RH2 = relative humidity from the field-collected data, RH2_y-1_ = relative humidity in year y-1 from the field-collected data, SD2 = saturation deficit from the field-collected data, SD2_y-1_ = saturation deficit in year y-1 from the field-collected data.

Table S12. The support for each individual explanatory variable is shown for the CAD. This support is calculated as the sum of the Akaike weights for all the models in the set that include that particular explanatory variable.

| **Rank** | **Explanatory variable of interest** | **Support (%)** |
| --- | --- | --- |
| 1 | Site | 100.0 |
| 2 | Beech tree mast score | 100.0 |
| 3 | Year | 99.1 |
| 4 | Site:Year | 98.2 |
| 5 | RH2 | 12.5 |
| 6 | PRy-1 | 10.4 |
| 7 | SD2y-1 | 10.1 |
| 8 | PR | 9.5 |
| 9 | RH2y-1 | 8.7 |
| 10 | SD2 | 4.9 |
| 11 | T1 | 4.7 |
| 12 | RH1y-1 | 4.7 |
| 13 | T2y-1 | 4.1 |
| 14 | SD1 | 3.8 |
| 15 | T1y-1 | 3.0 |
| 16 | T2 | 3.0 |
| 17 | SD1y-1 | 2.9 |
| 18 | RH1 | 2.9 |
| 19 | Site:T1 | < 1.0 |
| 20 | Site:T1y-1 | < 1.0 |
| 21 | Site:RH2 | < 1.0 |
| 22 | Site:T2 | < 1.0 |
| 23 | Site:SD2y-1 | < 1.0 |
| 24 | Site:SD1 | < 1.0 |
| 25 | Site:RH2y-1 | < 1.0 |
| 26 | Site:SD2 | < 1.0 |
| 27 | Site:SD1y-1 | < 1.0 |
| 28 | Site:PRy-1 | < 1.0 |
| 29 | Site:PR | < 1.0 |
| 30 | Site:RH1 | < 1.0 |
| 31 | Site:RH1y-1 | < 1.0 |
| 32 | Site:T2y-1 | < 1.0 |
| 33 | Spruce tree mast score | < 1.0 |
| 34 | Site:Beech tree mast score | < 1.0 |

The acronyms for the explanatory variables are as follows: T1 = temperature from the Climap-net data, T1_y-1_ = temperature in year y-1 from the Climap-net data, RH1 = relative humidity from the Climap-net data, RH1_y-1_ = relative humidity in year y-1 from the Climap-net data, SD1 = saturation deficit from the Climap-net data, SD1_y-1_ = saturation deficit in year y-1 from the Climap-net data, PR = precipitation from the Climap-net data, and PR_y-1_ = precipitation in year y-1 from the Climap-net data, T2 = temperature from the field-collected data, T2_y-1_ = temperature in year y-1 from the field-collected data, RH2 = relative humidity from the field-collected data, RH2_y-1_ = relative humidity in year y-1 from the field-collected data, SD2 = saturation deficit from the field-collected data, SD2_y-1_ = saturation deficit in year y-1 from the field-collected data.

Table S13. Model-averaged parameter estimates are shown for the linear models of the log10-transformed CAD response variable. Shown are the parameter types, the parameter names, the parameter estimates, and the 95% confidence limits (LL = lower limit and UL = upper limit). Estimate 1 is averaged over all the models in the set. Estimate 2 is averaged over the subset of models with a cumulative support of 95%. The 95% confidence limits are for estimate 2. Statistically significant parameter estimates are shown in bold-face type.

| **Type** | **Name** | **Estimate 1** | **Estimate 2** | **95% LL** | **95% UL** |
| --- | --- | --- | --- | --- | --- |
| **Intercept** | **Low site** | **2.818** | **2.818** | **2.483** | **3.153** |
| **Contrast 1** | **Medium site** | **0.583** | **0.583** | **0.177** | **0.990** |
| **Contrast 2** | **High site** | **0.567** | **0.567** | **0.134** | **1.001** |
| Contrast 3 | Top site | 0.404 | 0.404 | -0.094 | 0.903 |
| **Slope 1** | **Year** | **0.041** | **0.041** | **0.010** | **0.073** |
| **Slope 2** | **Beech tree mast score** | **0.100** | **0.100** | **0.060** | **0.139** |
| **Slope 3** | **Spruce tree mast score** | **0.000** | **0.095** | **0.017** | **0.173** |
| Slope 4 | T1 | -0.002 | -0.050 | -0.223 | 0.123 |
| Slope 5 | RH1 | 0.000 | 0.002 | -0.081 | 0.084 |
| Slope 6 | SD1 | -0.001 | -0.035 | -0.146 | 0.075 |
| Slope 7 | PR | 0.005 | 0.049 | -0.021 | 0.119 |
| Slope 8 | T1_y-1_ | 0.000 | -0.008 | -0.172 | 0.157 |
| Slope 9 | RH1_y-1_ | 0.002 | 0.040 | -0.051 | 0.131 |
| Slope 10 | SD1_y-1_ | 0.000 | -0.003 | -0.126 | 0.121 |
| Slope 11 | PR_y-1_ | 0.005 | 0.052 | -0.020 | 0.123 |
| Slope 12 | T2 | 0.000 | -0.004 | -0.101 | 0.092 |
| Slope 13 | RH2 | -0.007 | -0.055 | -0.126 | 0.017 |
| Slope 14 | SD2 | 0.002 | 0.040 | -0.048 | 0.127 |
| Slope 15 | T2_y-1_ | 0.001 | 0.034 | -0.058 | 0.126 |
| Slope 16 | RH2_y-1_ | -0.004 | -0.048 | -0.121 | 0.025 |
| Slope 17 | SD2_y-1_ | 0.006 | 0.064 | -0.027 | 0.155 |
| Contrast 4 | Medium site:Year | -0.031 | -0.031 | -0.074 | 0.011 |
| **Contrast 5** | **High site:Year** | **-0.055** | **-0.056** | **-0.099** | **-0.014** |
| **Contrast 6** | **Top site:Year** | **-0.085** | **-0.086** | **-0.129** | **-0.044** |
| Contrast 7 | Medium site:Beech tree mast score | 0.000 | 0.010 | -0.098 | 0.118 |
| Contrast 8 | High site:Beech tree mast score | 0.000 | -0.068 | -0.176 | 0.040 |
| Contrast 9 | High site:Beech tree mast score | 0.000 | -0.046 | -0.155 | 0.062 |
| Contrast 10 | Medium site:T1 | 0.000 | -0.050 | -0.412 | 0.312 |
| Contrast 11 | High site:T1 | 0.000 | -0.144 | -0.498 | 0.211 |
| Contrast 12 | Top site:T1 | -0.001 | -0.451 | -0.799 | -0.103 |
| Contrast 13 | Medium site:RH1 | 0.000 | 0.006 | -0.257 | 0.269 |
| Contrast 14 | High site:RH1 | 0.000 | -0.078 | -0.340 | 0.183 |
| Contrast 15 | Top site:RH1 | 0.000 | 0.103 | -0.151 | 0.356 |
| Contrast 16 | Medium site:SD1 | 0.000 | -0.023 | -0.300 | 0.254 |
| Contrast 17 | High site:SD1 | 0.000 | 0.006 | -0.283 | 0.295 |
| Contrast 18 | Top site:SD1 | 0.000 | -0.283 | -0.583 | 0.018 |
| Contrast 19 | Medium site:PR | 0.000 | 0.015 | -0.193 | 0.222 |
| Contrast 20 | High site:PR | 0.000 | 0.016 | -0.191 | 0.223 |
| Contrast 21 | Top site:PR | 0.000 | 0.077 | -0.128 | 0.282 |
| Contrast 22 | Medium site:T1_y-1_ | 0.000 | -0.173 | -0.569 | 0.223 |
| Contrast 23 | High site:T1_y-1_ | 0.000 | -0.298 | -0.684 | 0.089 |
| Contrast 24 | Top site:T1_y-1_ | 0.000 | -0.471 | -0.848 | -0.093 |
| Contrast 25 | Medium site:RH1_y-1_ | 0.000 | 0.009 | -0.286 | 0.305 |
| Contrast 26 | High site:RH1_y-1_ | 0.000 | 0.046 | -0.246 | 0.339 |
| Contrast 27 | Top site:RH1_y-1_ | 0.000 | 0.072 | -0.210 | 0.354 |
| Contrast 28 | Medium site:SD1_y-1_ | 0.000 | -0.075 | -0.406 | 0.256 |
| Contrast 29 | High site:SD1_y-1_ | 0.000 | -0.222 | -0.564 | 0.119 |
| Contrast 30 | Top site:SD1_y-1_ | 0.000 | -0.323 | -0.672 | 0.026 |
| Contrast 31 | Medium site:PR_y-1_ | 0.000 | -0.026 | -0.240 | 0.188 |
| Contrast 32 | High site:PR_y-1_ | 0.000 | -0.023 | -0.236 | 0.191 |
| Contrast 33 | Top site:PR_y-1_ | 0.000 | 0.052 | -0.160 | 0.264 |
| Contrast 34 | Medium site:T2 | 0.000 | 0.179 | -0.119 | 0.478 |
| Contrast 35 | High site:T2 | 0.000 | 0.091 | -0.206 | 0.387 |
| Contrast 36 | Top site:T2 | 0.000 | -0.122 | -0.418 | 0.174 |
| Contrast 37 | Medium site:RH2 | 0.000 | -0.137 | -0.344 | 0.070 |
| Contrast 38 | High site:RH2 | 0.000 | -0.097 | -0.321 | 0.127 |
| Contrast 39 | Top site:RH2 | 0.000 | 0.081 | -0.147 | 0.310 |
| Contrast 40 | Medium site:SD2 | 0.000 | 0.166 | -0.065 | 0.398 |
| Contrast 41 | High site:SD2 | 0.000 | 0.135 | -0.128 | 0.398 |
| Contrast 42 | Top site:SD2 | 0.000 | -0.067 | -0.375 | 0.241 |
| Contrast 43 | Medium site:T2_y-1_ | 0.000 | 0.092 | -0.230 | 0.415 |
| Contrast 44 | High site:T2_y-1_ | 0.000 | 0.086 | -0.234 | 0.406 |
| Contrast 45 | Top site:T2_y-1_ | 0.000 | 0.013 | -0.307 | 0.333 |
| Contrast 46 | Medium site:RH2_y-1_ | 0.000 | -0.149 | -0.358 | 0.061 |
| Contrast 47 | High site:RH2_y-1_ | 0.000 | -0.135 | -0.359 | 0.090 |
| Contrast 48 | Top site:RH2_y-1_ | 0.000 | 0.011 | -0.215 | 0.237 |
| Contrast 49 | Medium site:SD2_y-1_ | 0.000 | 0.200 | -0.031 | 0.432 |
| Contrast 50 | High site:SD2_y-1_ | 0.000 | 0.221 | -0.038 | 0.480 |
| Contrast 51 | Top site:SD2_y-1_ | 0.000 | 0.115 | -0.178 | 0.408 |

The acronyms for the explanatory variables are as follows: T1 = temperature from the Climap-net data, T1_y-1_ = temperature in year y-1 from the Climap-net data, RH1 = relative humidity from the Climap-net data, RH1_y-1_ = relative humidity in year y-1 from the Climap-net data, SD1 = saturation deficit from the Climap-net data, SD1_y-1_ = saturation deficit in year y-1 from the Climap-net data, PR = precipitation from the Climap-net data, and PR_y-1_ = precipitation in year y-1 from the Climap-net data, T2 = temperature from the field-collected data, T2_y-1_ = temperature in year y-1 from the field-collected data, RH2 = relative humidity from the field-collected data, RH2_y-1_ = relative humidity in year y-1 from the field-collected data, SD2 = saturation deficit from the field-collected data, SD2_y-1_ = saturation deficit in year y-1 from the field-collected data.


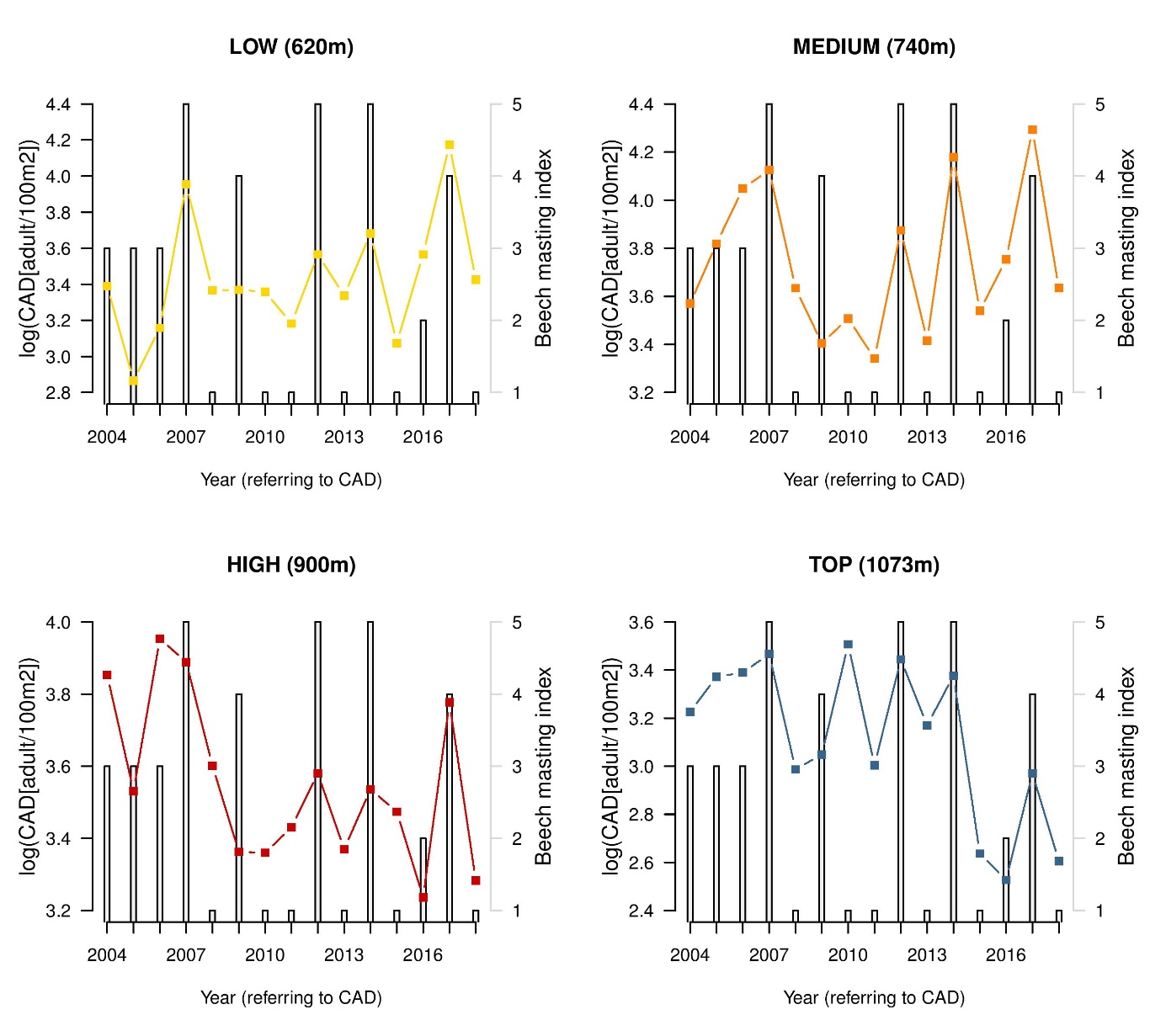
Figure S10. The log10-transformed cumulative adult density (CAD; points and solid lines) and the beech tree mast score (grey bars) are shown over time for each of the four elevations on Chaumont Mountain. The CAD increased significantly over the 15-year study period (2004 – 2018) at the low elevation site, but it decreased significantly at the high and top elevation. Years of high seed production by beech trees cause high CAD three years later. The CAD is the total number of questing *I. ricinus* adults sampled by the dragging method each year. Beech tree mast score ranges from 1 to 5 (class 1 = very poor, 2 = poor, 3 = moderate, 4 = good, and class 5 = full mast year).


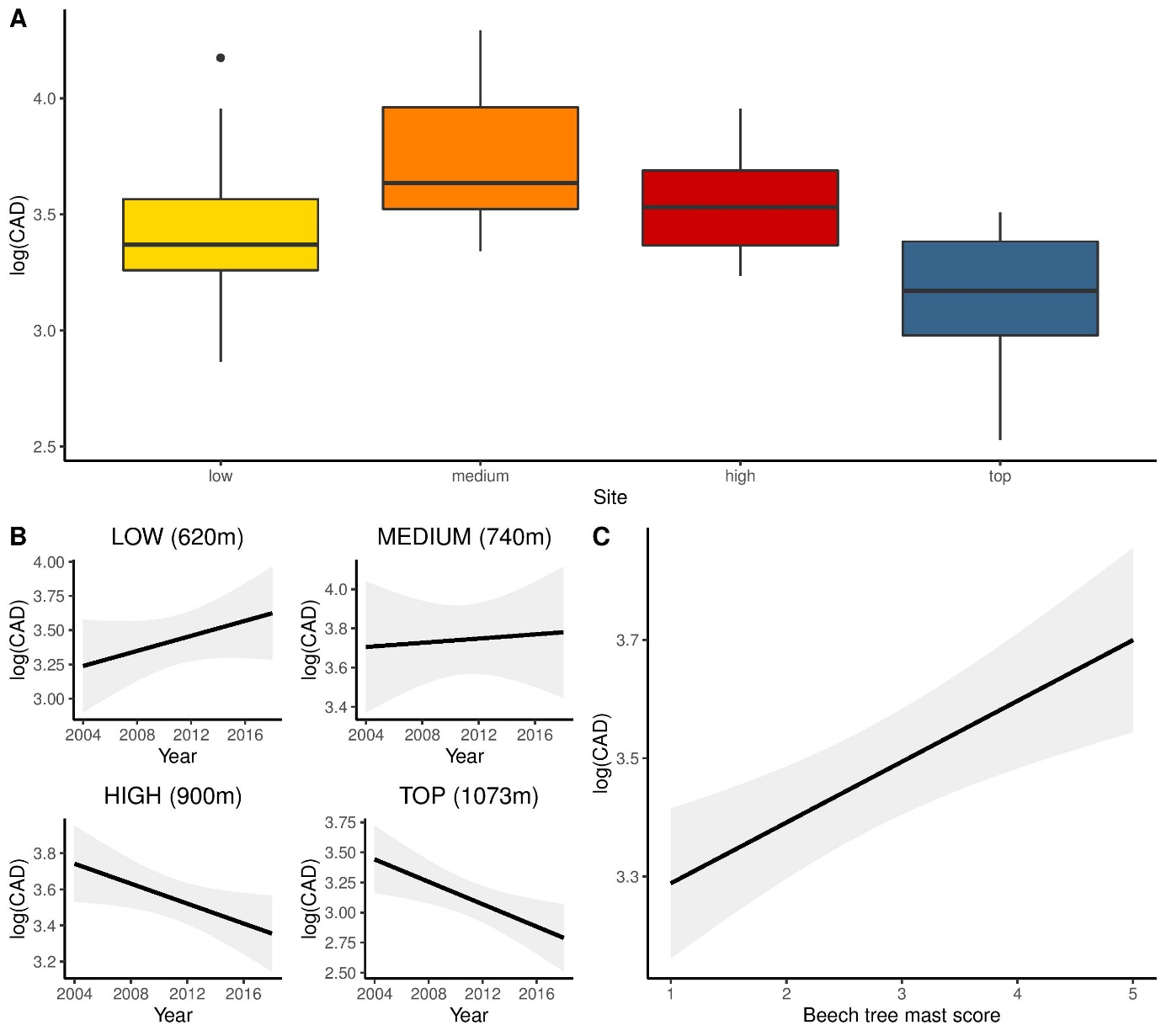
Figure S11. Effect sizes of the explanatory variables (elevation, year, site:year interaction, and beech mast score 2 years prior) on the log10-transformed cumulative adult density (CAD). The parameter estimates used to calculate the effect sizes were taken from the model-averaged in Table S13. (A) Effect of elevation on the CAD. The CAD at the medium, high, and top elevation was 256.6%, 224.6%, and 108.0% higher than the low elevation, respectively (partial r^2^ = 47.7%). (B) Effect of year (e.g. time) on the CAD. The CAD increased by 280.3% at the low elevation and by 38.9% at the medium elevation, but decreased by 37.7% at the high elevation and by 76.5% at the top elevation (partial r2 = 9.8%). (C) Effect of beech mast score on the CAD. Increasing the beech mast score from 1 (poor mast) to 5 (full mast) increased the CAD by 150.4% (partial r2 = 21.0%).

# SECTION 6 – Full model selection table, support of each individual explanatory variable, and model-averaged parameter estimates of the nymph abundance for the three lowest elevation sites

**Methods:** We used a model selection approach based on the Akaike information criterion (AIC) to find the most parsimonious model. Models were ranked according to their AIC values and the Akaike weights were calculated for each model. We used the Akaike weights to calculate the model-averaged parameter estimates and their 95% confidence intervals (CIs). The support for a given explanatory variable of interest was calculated as the sum of the Akaike weights of all the models in the set that included that particular explanatory variable. The support for a given explanatory variable ranged from low (0.0%) to high (100%).

**Results:** For the CND, the best six models had a combined support of 95.0% (Table S14). The other 46 models had a combined support of 5% (Table S14). The best six models all contained the explanatory variables of elevation site, year, and beech mast score, but they differed with respect to the identity of the climate variable. The best model had 76.0% of the support, explained 73.2% of the variation in the CND, and contained the explanatory variables of elevation site (partial r^2^ = 26.6%), year (partial r^2^ = 14.8%), beech tree mast score from 2 years prior (partial r^2^ = 26.9%), and the field-collected relative humidity from the same year (partial r^2^ = 7.6%) (Table S14). For the individual explanatory variables, there was strong support for the main effects of site (100.0%), beech tree mast score from 2 years prior (100.0%), year (99.9%), and field-collected relative humidity from the same year (81.6%; Table S15). None of the other explanatory variables had a support > 7.0% (Table S15).

We calculated the model-averaged parameter estimates to make robust inferences about the relationships between the explanatory variables and the CND (Table S16). We calculated the effect sizes with respect to the following baseline: the site was low elevation, the year was 2004, the beech tree mast was 1, and the field-collected relative humidity from the same year was 50.0% (Table S17).

The CND was significantly different between the three elevation sites (Figure S12). The CND at the low elevation was 10.3% higher than the medium elevation (Medium – Low contrast = -0.047, 95% CI = -0.159 – 0.065; Figure S13, Tables S18 and S19). The CND at the low elevation was 41.9% higher than the high elevation (High – Low = -0.236, 95% CI = -0.363 – 0.109; Figure S13, Tables S18 and S19). In summary, the CND was inversely related to altitude; it was highest at the low elevation and lowest at the high elevation (Figure S12 and Figure S13).

The slope of the covariate year was positive (0.020 per year; Table S16) and the 95% CI did not overlap zero (0.009– 0.031), indicating that the CND was increasing over time at Chaumont Mountain (Figure S12). Over the 15-year period of the study (2004 – 2018), the CND increased by 90.5% at the low site (slope = 0.020, 95% CI = 0.009 – 0.031), at the medium site (slope = -0.006, 95% CI = -0.030 – 0.017), and at the high site (slope = -0.011, 95% CI = -0.035 – 0.012) (Figure S13).

The covariate beech mast score had a strong positive effect on the CND two years later (0.067 per class; Table S16) and the 95% CI did not overlap zero (0.045– 0.089), indicating that the CND increased with masting 2 years prior (Figure S12). Increasing the beech mast score from 1 (poor mast) to 5 (full mast) increased the CND by 85.6% at each of the three elevation sites on Chaumont Mountain (Figure S13; Table 19).

The slope of the field-collected relative humidity from the same year was negative (-0.074 per standard deviation; Table S16) and the 95% CI did not overlap zero (-0.117– -0.032), indicating that the CND decreased with the field-collected relative humidity in the same year. Increasing the field-collected relative humidity from 50.0% to 75.0% decreased the CND from the same year by 46.4% at each of the three elevation sites on Chaumont Mountain (Figure S13; Table S17). Temperature had a positive effect on the CND (slope = 0.077 per standard deviation, 95% CI = 0.018 – 0.137; Table S16) and saturation deficit had a positive effect on the CND (slope = 0.068 per standard deviation, 95% CI = 0.015 – 0.120; Table S16). In summary, our study found that years with higher temperatures and lower relative humidity have higher annual estimates of the CND compared to years with lower temperatures and high relative humidity. According to our model selection table, the level of support for relative humidity (81.6% in Table S15) is 14.3 and 14.1 times higher than that of temperature (5.7% in Table S15) and saturation deficit (5.8% in Table S15), respectively.

In summary, the CND increased significantly over time at the three lower elevations. The CND increased with beech tree seed production two years earlier while it decreased significantly with the field-collected relative humidity in the same year (Table S17).

Table S14. Model selection results are shown for the linear models with normal errors of the log10-transformed CND response variable. The explanatory variables were elevation site, year, tree masting variables obtained from MASTREE, and the climate variables obtained from the Climap-net and collected in the field. Shown for each model are the model rank (Rank), model structure (see below for explanation of explanatory variables), model degrees of freedom (Df), log-likelihood (logLik), Akaike information criterion (AIC), difference in the AIC value from the top model (ΔAIC), model weight (Weight1), cumulative weight (Weight2), and adjusted r-squared (r^2^).

| **Rank** | **Model structure** | **Df** | **logLik** | **AIC** | **ΔAIC** | **Weight1** | **Weight2** | **r^2^** |
| --- | --- | --- | --- | --- | --- | --- | --- | --- |
| 1 | CND ~ S+Y+B+RH2 | 7 | 33.0 | -48.7 | 0.0 | 76.0 | 76.0 | 73.2 |
| 2 | CND ~ S+Y+B+RH2+S:Y | 9 | 33.6 | -43.6 | 5.2 | 6.0 | 82.0 | 72.4 |
| 3 | CND ~ S+Y+B+SD2 | 7 | 30.4 | -43.5 | 5.3 | 5.0 | 87.0 | 69.7 |
| 4 | CND ~ S+Y+B+T2 | 7 | 30.4 | -43.4 | 5.3 | 5.0 | 92.0 | 69.6 |
| 5 | CND ~ S+Y+B+RH2_y-1_ | 7 | 29.4 | -41.4 | 7.3 | 2.0 | 94.0 | 68.1 |
| 6 | CND ~ S+Y+B+SD2_y-1_ | 7 | 29.1 | -40.8 | 7.9 | 1.0 | 95.0 | 67.7 |
| 7 | CND ~ S+Y+B | 6 | 26.6 | -38.9 | 9.8 | 1.0 | 96.0 | 64.7 |
| 8 | CND ~ S+Y+B+T1 | 7 | 28.0 | -38.6 | 10.1 | 0.0 | 96.0 | 65.9 |
| 9 | CND ~ S+Y+B+PR | 7 | 27.9 | -38.6 | 10.1 | 0.0 | 96.0 | 65.9 |
| 10 | CND ~ S+Y+B+T2+S:Y | 9 | 30.9 | -38.3 | 10.5 | 0.0 | 96.0 | 68.7 |
| 11 | CND ~ S+Y+B+SD2+S:Y | 9 | 30.8 | -38.0 | 10.7 | 0.0 | 96.0 | 68.5 |
| 12 | CND ~ S+Y+B+T2_y-1_ | 7 | 27.2 | -37.1 | 11.6 | 0.0 | 96.0 | 64.7 |
| 13 | CND ~ S+Y+B+S:B | 8 | 28.6 | -36.8 | 11.9 | 0.0 | 96.0 | 66.0 |
| 14 | CND ~ S+Y+B+SD1 | 7 | 26.9 | -36.6 | 12.2 | 0.0 | 96.0 | 64.2 |
| 15 | CND ~ S+Y+B+RH1_y-1_ | 7 | 26.9 | -36.5 | 12.2 | 0.0 | 96.0 | 64.2 |
| 16 | CND ~ S+Y+B+T1_y-1_ | 7 | 26.9 | -36.4 | 12.3 | 0.0 | 96.0 | 64.1 |
| 17 | CND ~ S+Y+B+RH2_y-1_+S:Y | 9 | 30.0 | -36.4 | 12.3 | 0.0 | 96.0 | 67.3 |
| 18 | CND ~ S+Y+B+Pr_y-1_ | 7 | 26.8 | -36.3 | 12.4 | 0.0 | 96.0 | 64.0 |
| 19 | CND ~ S+Y+B+SD1_y-1_ | 7 | 26.7 | -36.2 | 12.6 | 0.0 | 96.0 | 63.9 |
| 20 | CND ~ S+Y+B+RH1 | 7 | 26.7 | -36.1 | 12.7 | 0.0 | 96.0 | 63.8 |
| 21 | CND ~ S+Y+B+SD2_y-1_+S:Y | 9 | 29.7 | -35.7 | 13.0 | 0.0 | 96.0 | 66.7 |
| 22 | CND ~ S+Y+B+S:Y | 8 | 27.2 | -34.0 | 14.7 | 0.0 | 96.0 | 63.6 |
| 23 | CND ~ S+Y+B+T1+S:Y | 9 | 28.5 | -33.5 | 15.3 | 0.0 | 96.0 | 64.9 |
| 24 | CND ~ S+Y+B+PR+S:Y | 9 | 28.4 | -33.2 | 15.5 | 0.0 | 96.0 | 64.8 |
| 25 | CND ~ S+B+T1+S:T1 | 8 | 26.5 | -32.7 | 16.0 | 0.0 | 96.0 | 62.5 |
| 26 | CND ~ S+Y+B+T2_y-1_+S:Y | 9 | 27.7 | -31.9 | 16.9 | 0.0 | 96.0 | 63.6 |
| 27 | CND ~ S+B+RH2+S:RH2 | 8 | 25.9 | -31.5 | 17.2 | 0.0 | 96.0 | 61.4 |
| 28 | CND ~ S+Y+B+S:Y+S:B | 10 | 29.2 | -31.3 | 17.4 | 0.0 | 96.0 | 65.0 |
| 29 | CND ~ S+Y+B+SD1+S:Y | 9 | 27.4 | -31.3 | 17.5 | 0.0 | 96.0 | 63.1 |
| 30 | CND ~ S+Y+B+T1_y-1_+S:Y | 9 | 27.4 | -31.2 | 17.5 | 0.0 | 96.0 | 63.0 |
| 31 | CND ~ S+Y+B+RH1_y-1_+S:Y | 9 | 27.4 | -31.1 | 17.6 | 0.0 | 96.0 | 62.9 |
| 32 | CND ~ S+Y+B+PR_y-1_+S:Y | 9 | 27.3 | -31.1 | 17.7 | 0.0 | 96.0 | 62.9 |
| 33 | CND ~ S+Y+B+SD1_y-1_+S:Y | 9 | 27.3 | -30.9 | 17.8 | 0.0 | 96.0 | 62.7 |
| 34 | CND ~ S+B | 5 | 21.3 | -30.8 | 17.9 | 0.0 | 96.0 | 55.6 |
| 35 | CND ~ S+Y+B+RH1+S:Y | 9 | 27.2 | -30.8 | 17.9 | 0.0 | 96.0 | 62.6 |
| 36 | CND ~ S+B+T1_y-1_+S:T1_y-1_ | 8 | 25.2 | -30.1 | 18.7 | 0.0 | 96.0 | 60.1 |
| 37 | CND ~ S+B+SD2+S:SD2 | 8 | 24.7 | -29.0 | 19.8 | 0.0 | 96.0 | 59.0 |
| 38 | CND ~ S+B+T2+S:T2 | 8 | 24.4 | -28.4 | 20.4 | 0.0 | 96.0 | 58.4 |
| 39 | CND ~ S+B+S:B | 7 | 22.7 | -28.2 | 20.5 | 0.0 | 96.0 | 56.4 |
| 40 | CND ~ S+B+SD1+S:SD1 | 8 | 24.0 | -27.6 | 21.1 | 0.0 | 96.0 | 57.7 |
| 41 | CND ~ S+B+SD1_y-1_+S:SD1_y-1_ | 8 | 22.6 | -24.9 | 23.9 | 0.0 | 96.0 | 54.8 |
| 42 | CND ~ S+B+Pr_y-1_+S:Pr_y-1_ | 8 | 22.4 | -24.5 | 24.2 | 0.0 | 96.0 | 54.4 |
| 43 | CND ~ S+B+RH2_y-1_+S:RH2_y-1_ | 8 | 22.3 | -24.3 | 24.5 | 0.0 | 96.0 | 54.2 |
| 44 | CND ~ S+B+RH1+S:RH1 | 8 | 22.1 | -23.8 | 24.9 | 0.0 | 96.0 | 53.7 |
| 45 | CND ~ S+B+PR+S:PR | 8 | 22.1 | -23.8 | 25.0 | 0.0 | 96.0 | 53.6 |
| 46 | CND ~ S+B+RH1_y-1_+S:RH1_y-1_ | 8 | 21.6 | -22.9 | 25.9 | 0.0 | 96.0 | 52.6 |
| 47 | CND ~ S+B+SD2_y-1_+S:SD2_y-1_ | 8 | 21.6 | -22.8 | 25.9 | 0.0 | 96.0 | 52.6 |
| 48 | CND ~ S+B+T2_y-1_+S:T2_y-1_ | 8 | 21.4 | -22.3 | 26.4 | 0.0 | 96.0 | 52.0 |
| 49 | CND ~ S+Y | 5 | 13.3 | -15.0 | 33.7 | 0.0 | 96.0 | 35.3 |
| 50 | CND ~ B | 3 | 9.7 | -12.8 | 36.0 | 0.0 | 96.0 | 26.9 |
| 51 | CND ~ S+Y+S:Y | 7 | 13.6 | -9.9 | 38.8 | 0.0 | 96.0 | 32.6 |
| 52 | CND ~ P | 3 | 2.8 | 0.9 | 49.7 | 0.0 | 96.0 | 0.0 |

The acronyms for the explanatory variables are as follows: S = site, Y = year, B = beech tree mast score, P = spruce tree mast score, T1 = temperature from the Climap-net data, T1_y-1_ = temperature in year y-1 from the Climap-net data, RH1 = relative humidity from the Climap-net data, RH1_y-1_ = relative humidity in year y-1 from the Climap-net data, SD1 = saturation deficit from the Climap-net data, SD1_y-1_ = saturation deficit in year y-1 from the Climap-net data, PR = precipitation from the Climap-net data, and PR_y-1_ = precipitation in year y-1 from the Climap-net data, T2 = temperature from the field-collected data, T2_y-1_ = temperature in year y-1 from the field-collected data, RH2 = relative humidity from the field-collected data, RH2_y-1_ = relative humidity in year y-1 from the field-collected data, SD2 = saturation deficit from the field-collected data, SD2_y-1_ = saturation deficit in year y-1 from the field-collected data.

Table S15. The support for each individual explanatory variable is shown for the CND. This support is calculated as the sum of the Akaike weights for all the models in the set that include that particular explanatory variable.

| **Rank** | **Explanatory variable of interest** | **Support (%)** |
| --- | --- | --- |
| 1 | Site | 100.0 |
| 2 | Beech tree mast score | 100.0 |
| 3 | Year | 99.9 |
| 4 | RH2 | 81.6 |
| 5 | Site:Year | 7.0 |
| 6 | SD2 | 5.8 |
| 7 | T2 | 5.7 |
| 8 | RH2_y-1_ | 2.1 |
| 9 | SD2_y-1_ | 1.6 |
| 10 | Spruce tree mast score | < 1.0 |
| 11 | T1 | < 1.0 |
| 12 | RH1 | < 1.0 |
| 13 | SD1 | < 1.0 |
| 14 | PR | < 1.0 |
| 15 | T1_y-1_ | < 1.0 |
| 16 | RH1_y-1_ | < 1.0 |
| 17 | SD1_y-1_ | < 1.0 |
| 18 | PR_y-1_ | < 1.0 |
| 19 | T2_y-1_ | < 1.0 |
| 20 | Site:Beech tree mast score | < 1.0 |
| 21 | Site:T1 | < 1.0 |
| 22 | Site:RH1 | < 1.0 |
| 23 | Site:SD1 | < 1.0 |
| 24 | Site:PR | < 1.0 |
| 25 | Site:T1_y-1_ | < 1.0 |
| 26 | Site:RH1_y-1_ | < 1.0 |
| 27 | Site:SD1_y-1_ | < 1.0 |
| 28 | Site:PR_y-1_ | < 1.0 |
| 29 | Site:T2 | < 1.0 |
| 30 | Site:RH2 | < 1.0 |
| 31 | Site:SD2 | < 1.0 |
| 32 | Site:T2_y-1_ | < 1.0 |
| 33 | Site:RH2_y-1_ | < 1.0 |
| 34 | Site:SD2_y-1_ | < 1.0 |

The acronyms for the explanatory variables are as follows: T1 = temperature from the Climap-net data, T1_y-1_ = temperature in year y-1 from the Climap-net data, RH1 = relative humidity from the Climap-net data, RH1_y-1_ = relative humidity in year y-1 from the Climap-net data, SD1 = saturation deficit from the Climap-net data, SD1_y-1_ = saturation deficit in year y-1 from the Climap-net data, PR = precipitation from the Climap-net data, and PR_y-1_ = precipitation in year y-1 from the Climap-net data, T2 = temperature from the field-collected data, T2_y-1_ = temperature in year y-1 from the field-collected data, RH2 = relative humidity from the field-collected data, RH2_y-1_ = relative humidity in year y-1 from the field-collected data, SD2 = saturation deficit from the field-collected data, SD2_y-1_ = saturation deficit in year y-1 from the field-collected data.

Table S16. Model-averaged parameter estimates are shown for the linear models of the log10-transformed CND response variable. Shown are the parameter types, the parameter names, the parameter estimates, and the 95% confidence limits (LL = lower limit and UL = upper limit). Estimate 1 is averaged over all the models in the set. Estimate 2 is averaged over the subset of models with a cumulative support of 95%. The 95% confidence limits are for estimate 2.

| **Type** | **Name** | **Estimate 1** | **Estimate 2** | **95% LL** | **95% UL** |
| --- | --- | --- | --- | --- | --- |
| Intercept | Low site | 3.934 | 3.934 | 3.802 | 4.067 |
| Contrast 1 | Medium site | -0.047 | -0.047 | -0.159 | 0.065 |
| **Contrast 2** | **High site** | **-0.236** | **-0.236** | **-0.363** | **-0.109** |
| **Slope 1** | **Year** | **0.020** | **0.020** | **0.009** | **0.031** |
| **Slope 2** | **Beech tree mast score** | **0.067** | **0.067** | **0.045** | **0.089** |
| Slope 3 | Spruce tree mast score | 0.000 | 0.019 | -0.038 | 0.075 |
| Slope 4 | T1 | 0.000 | 0.080 | -0.032 | 0.193 |
| Slope 5 | RH1 | 0.000 | -0.007 | -0.066 | 0.052 |
| Slope 6 | SD1 | 0.000 | 0.026 | -0.049 | 0.101 |
| Slope 7 | PR | 0.000 | 0.035 | -0.012 | 0.082 |
| Slope 8 | T1_y-1_ | 0.000 | 0.039 | -0.089 | 0.166 |
| Slope 9 | RH1_y-1_ | 0.000 | 0.024 | -0.050 | 0.098 |
| Slope 10 | SD1_y-1_ | 0.000 | 0.016 | -0.072 | 0.105 |
| Slope 11 | PR_y-1_ | 0.000 | -0.013 | -0.064 | 0.039 |
| **Slope 12** | **T2** | **0.004** | **0.077** | **0.018** | **0.137** |
| **Slope 13** | **RH2** | **-0.061** | **-0.074** | **-0.117** | **-0.032** |
| **Slope 14** | **SD2** | **0.004** | **0.068** | **0.015** | **0.120** |
| Slope 15 | T2_y-1_ | 0.000 | 0.032 | -0.034 | 0.099 |
| **Slope 16** | **RH2_y-1_** | **-0.001** | **-0.055** | **-0.105** | **-0.005** |
| **Slope 17** | **SD2_y-1_** | **0.001** | **0.062** | **0.002** | **0.123** |
| Contrast 3 | Medium site:Year | 0.000 | -0.006 | -0.030 | 0.017 |
| Contrast 4 | High site:Year | -0.001 | -0.011 | -0.035 | 0.012 |
| Contrast 5 | Medium site:Beech tree mast score | 0.000 | 0.016 | -0.044 | 0.075 |
| Contrast 6 | High site: Beech tree mast score | 0.000 | 0.053 | -0.007 | 0.112 |
| Contrast 7 | Medium site:T1 | 0.000 | -0.080 | -0.292 | 0.133 |
| Contrast 8 | High site:T1 | 0.000 | -0.073 | -0.281 | 0.135 |
| Contrast 9 | Medium site:RH1 | 0.000 | 0.075 | -0.084 | 0.235 |
| Contrast 10 | High site:RH1 | 0.000 | 0.005 | -0.154 | 0.164 |
| Contrast 11 | Medium site:SD1 | 0.000 | -0.065 | -0.231 | 0.101 |
| Contrast 12 | High site:SD1 | 0.000 | 0.004 | -0.169 | 0.176 |
| Contrast 13 | Medium site:PR | 0.000 | 0.050 | -0.077 | 0.177 |
| Contrast 14 | High site:PR | 0.000 | -0.018 | -0.145 | 0.109 |
| Contrast 15 | Medium site:T1_y-1_ | 0.000 | -0.136 | -0.370 | 0.099 |
| Contrast 16 | High site:T1_y-1_ | 0.000 | -0.177 | -0.406 | 0.051 |
| Contrast 17 | Medium site:RH1_y-1_ | 0.000 | 0.013 | -0.168 | 0.194 |
| Contrast 18 | High site:RH1_y-1_ | 0.000 | -0.043 | -0.223 | 0.136 |
| Contrast 19 | Medium site:SD1_y-1_ | 0.000 | -0.038 | -0.241 | 0.165 |
| Contrast 20 | High site:SD1_y-1_ | 0.000 | -0.008 | -0.218 | 0.201 |
| Contrast 21 | Medium site:PR_y-1_ | 0.000 | -0.001 | -0.131 | 0.129 |
| Contrast 22 | High site:PR_y-1_ | 0.000 | -0.002 | -0.132 | 0.127 |
| Contrast 23 | Medium site:T2 | 0.000 | -0.064 | -0.242 | 0.115 |
| Contrast 24 | High site:T2 | 0.000 | 0.047 | -0.130 | 0.224 |
| Contrast 25 | Medium site:RH2 | 0.000 | -0.051 | -0.171 | 0.069 |
| Contrast 26 | High site:RH2 | 0.000 | -0.102 | -0.232 | 0.027 |
| Contrast 27 | Medium site:SD2 | 0.000 | 0.045 | -0.091 | 0.180 |
| Contrast 28 | High site:SD2 | 0.000 | 0.144 | -0.010 | 0.297 |
| Contrast 29 | Medium site:T2_y-1_ | 0.000 | 0.018 | -0.179 | 0.215 |
| Contrast 30 | High site:T2_y-1_ | 0.000 | 0.016 | -0.179 | 0.212 |
| Contrast 31 | Medium site:RH2_y-1_ | 0.000 | 0.013 | -0.117 | 0.143 |
| Contrast 32 | High site:RH2_y-1_ | 0.000 | -0.058 | -0.197 | 0.081 |
| Contrast 33 | Medium site:SD2_y-1_ | 0.000 | 0.008 | -0.139 | 0.156 |
| Contrast 34 | High site:SD2_y-1_ | 0.000 | 0.054 | -0.111 | 0.218 |

The acronyms for the explanatory variables are as follows: T1 = temperature from the Climap-net data, T1_y-1_ = temperature in year y-1 from the Climap-net data, RH1 = relative humidity from the Climap-net data, RH1_y-1_ = relative humidity in year y-1 from the Climap-net data, SD1 = saturation deficit from the Climap-net data, SD1_y-1_ = saturation deficit in year y-1 from the Climap-net data, PR = precipitation from the Climap-net data, and PR_y-1_ = precipitation in year y-1 from the Climap-net data, T2 = temperature from the field-collected data, T2_y-1_ = temperature in year y-1 from the field-collected data, RH2 = relative humidity from the field-collected data, RH2_y-1_ = relative humidity in year y-1 from the field-collected data, SD2 = saturation deficit from the field-collected data, SD2_y-1_ = saturation deficit in year y-1 from the field-collected data.


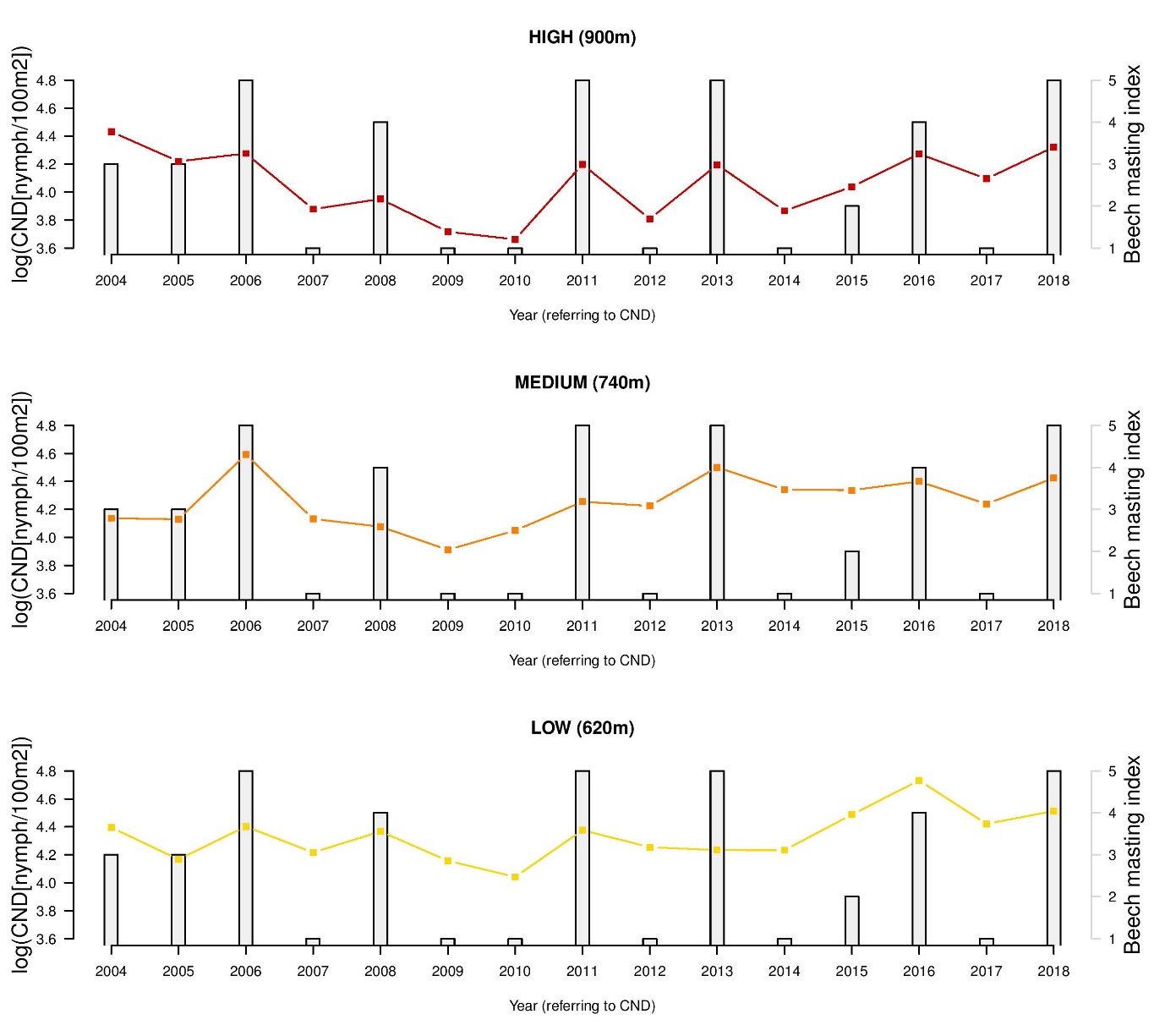


Figure S12. The log10-transformed cumulative nymphal density (CND) and the beech tree mast score over time is shown for each of the three elevations on Chaumont Mountain. The CND increased significantly over the 15-year study period (2004–2018). Years of high seed production by beech trees are strongly positively associated with high CND two years later. The CND is the total number of questing *I. ricinus* nymphs sampled by the dragging method each year. Beech tree mast scores range from 1 to 5 (1 = very poor mast; 2 = poor; 3 = moderate; 4 = good; and 5 = full mast year). The solid lines and barplots represent the CND and the beech tree mast score, respectively.
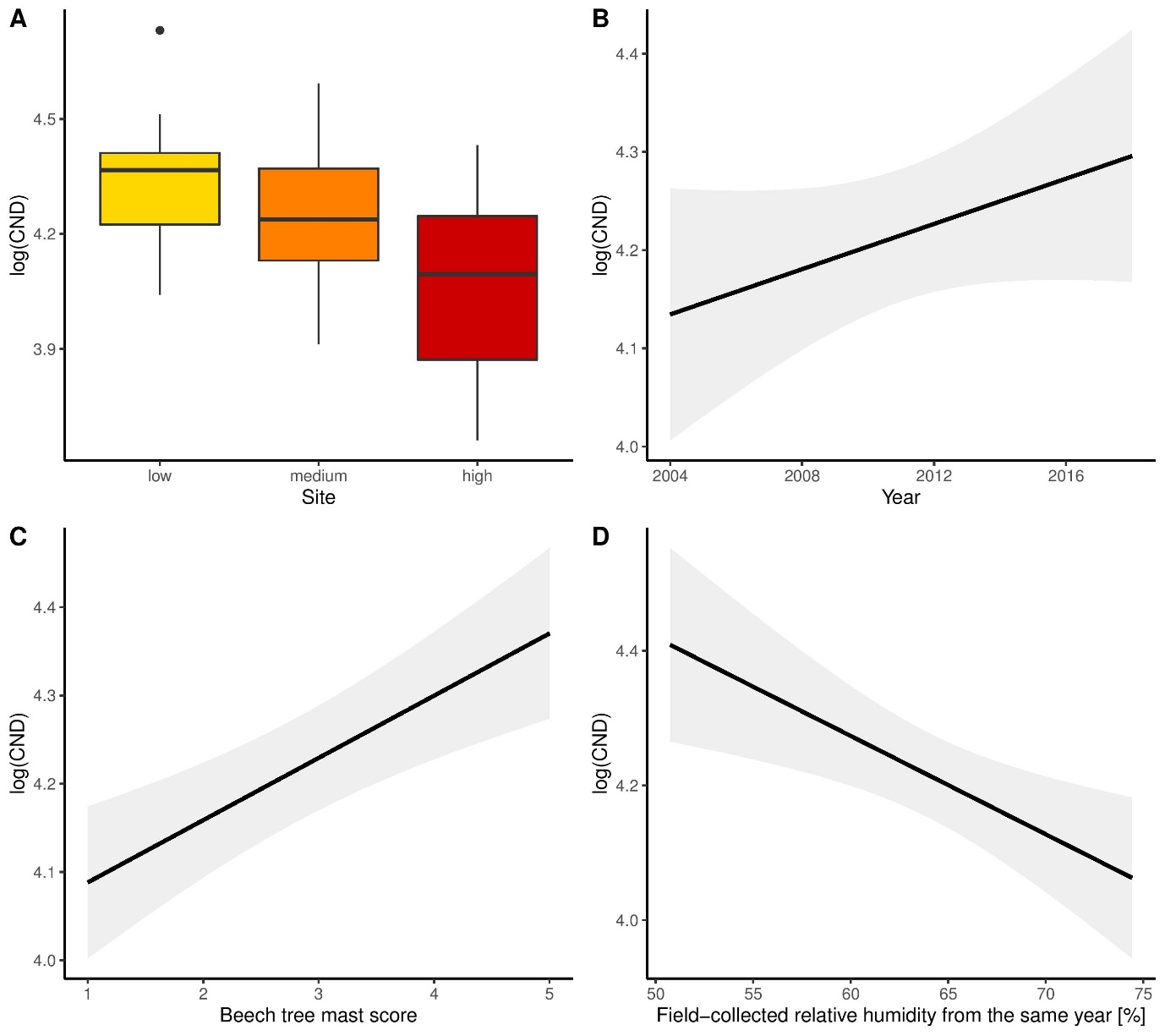
Figure S13. Effect sizes of the explanatory variables (elevation, year, beech mast score 2 years prior, and mean annual relative humidity in the same year) on the log10-transformed cumulative nymphal density (CND). The parameter estimates used to calculate the effect sizes were taken from the model-averaged in Table S16. (A) Effect of elevation on the CND. The CND at the low elevation was 10.3% higher than the medium elevation and 41.9% higher than the high elevation (partial r^2^ = 26.6%). (B) Effect of year (e.g. time) on the CND. The CND increased by 90.5% over the 15-year study period (partial r^2^ = 14.8%). (C) Effect of beech mast score on the CND. Increasing the beech mast score from 1 (poor mast) to 5 (full mast) increased the CND by 85.6% (partial r^2^ = 26.9%). (D) Effect of the mean annual field-collected relative humidity on the CND in the same year. Increasing the field-collected relative humidity from 50.0% to 75.0% decreased the CND by 46.4% (partial r^2^ = 7.6%).

# SECTION 7 – Assumptions of the linear models for the best models from the AIC-based model selection approach of the nymph and adult abundance for the three lowest elevation sites

**Methods:** Linear models assume that the residuals follow a normal distribution and that the variance of the residuals is the same over the range of predicted values (or between groups). We used the Shapiro-Wilk normality test to test the assumption of normality for the log10-transformed CND residuals. We used a Bartlett’s K-squared test to test whether the variance of the residuals was the same between the three sites.

**Results for nymphal abundance:** For the nymphal abundance, the residuals of the best model (model 1 in Table S8) followed a normal distribution (Shapiro-Wilk normality test: W = 0.985, p = 0.843) and these residuals had the same variance between the three elevation sites (Bartlett’s K-squared = 0.664, df = 2, p = 0.717).

**Results for adult tick abundance:** For the adult tick abundance, the residuals of the best model (model 1 in Table S11) followed a normal distribution (Shapiro-Wilk normality test: W = 0.985, p = 0.705) and these residuals had the same variance between the three elevation sites (Bartlett’s K-squared = 2.594, df = 2, p = 0.273).

# SECTION 8 – Parameter estimates of the top model in the model selection table of the nymph abundance for the three lowest elevation sites

Table S17. The parameter estimates from the top model in the model selection table (Table S14) are shown. In this top model, the log10-transformed CND response variable was modelled as a function of elevation site, year, beech tree mast score 2 years prior, and the field-collected mean annual relative humidity in the same year. Shown are the parameter types, parameter names, parameter estimates on the log10-transformed scale, standard errors (s.e.), t-statistic (t), and p-values (p).

| **Type** | **Name** | **Estimate** | **s.e** | **t** | **p** |
| --- | --- | --- | --- | --- | --- |
| **Intercept** | **Low site** | **3.939** | **0.059** | **66.209** | **< 0.001** |
| Contrast 1 | Medium site | -0.053 | 0.045 | -1.162 | 0.253 |
| **Contrast 2** | **High site** | **-0.245** | **0.047** | **-5.217** | **< 0.001** |
| **Slope 1** | **Year** | **0.020** | **0.005** | **4.262** | **< 0.001** |
| **Slope 2** | **Beech tree mast score** | **0.067** | **0.011** | **6.378** | **< 0.001** |
| **Slope 3** | **Relative humidity (field-collected)** | **-0.074** | **0.021** | **-3.570** | **0.001** |

# SECTION 9 – Time lag between the nymph abundance and beech masting

Methods – Relationship between beech masting and CND with different time lags: To test the relationship between beech masting and CND across years, we used linear models to model the log10-transformed CND as a function of the beech mast score but with different time lags. We analyzed 9 different beech mast score explanatory variables (beech_y-4_, beech_y-3_, beech_y-2_, beech_y-1_, beech, beech_y+1_, beech_y+2_, beech_y+3_, beech_y+4_) where the time lag was allowed to shift from – 4 years to +4 years relative to the CND, which was always the same. Each CND was modelled as an ANCOVA of the beech mast score, site, and their interaction (Table S18).

**Results – Relationship between beech masting and CND with different time lags:** For all models, the interaction between the beech mast score and site was not significant (Table S18). We therefore ran all models without the interaction and tested the main effects (models 10 to 18 in Table S19). As expected from the biological chain reported firstly in the USA (Ostfeld et al. 2006), the coefficient of determination between the CND and beech mast score was maximal for the y-2 time lag (Table S19).

**Methods – Variation in CND explained by time lag:** The previous analysis led us to determine how much of the variation was explained by the time lag. We modeled the log10-transformed CND as a function of 9 different times lagged beech mast score alone, and as a function of site alone (Table S20). We performed a variance partitioning of the main effects model (Table S19) to evaluate how much variation could be attributed to the time lag. In this approach, we compared the multiple r^2^ values between the main effect models (Table S19) and the models with a single explanatory factor (Table S20) to calculate the % variation in CND explained by the time lag (Table S21).

**Results – Variation in CND explained by time lag:** As expected, the best model had a two-year time lag between nymphs and beech mast score (model 12 in Table S19). This model had an r^2^ value of 50.2%, and the main effects of the beech_y-2_ (F_41, 45_ = 24.687, p < 0.001) and site (F_41, 43_ = 11.306, p < 0.001) were both highly significant. According to the variance partitioning method, beech_y-2_ and site accounted for a minimum of 25.6% and 2.4% of the variation in CND, respectively (Table S21). The parameter estimates for model 12 showed the expected positive relationship between beech_y-2_ and CND (slope = 0.070, s.e. of slope = 0.014; Table S22). Altogether, these results show that the emphasize of the 2-year biological correlation of our study is accurate and not overinterpreted.

Table S18. The log10-transformed CND was modeled as an ANCOVA of the beech mast score with different time lags, site, and their interaction. Shown are the time lags (Lag), sample size (N), F-statistic of the interaction (Finter), and p-value of the interaction (pinter).

| **Model No.** | **Model Structure** | **Lag** | **N** | **F_inter_** | **p_inter_** |
| --- | --- | --- | --- | --- | --- |
| 1 | log(CND) ~ beech_y-4_ + site + beech_y-4_:site | -4 | 45 | 0.201 | 0.819 |
| 2 | log(CND) ~ beech_y-3_ + site + beech_y-3_:site | -3 | 45 | 0.144 | 0.866 |
| 3 | log(CND) ~ beech_y-2_ + site + beech_y-2_:site | -2 | 45 | 1.310 | 0.281 |
| 4 | log(CND) ~ beech_y-1_ + site + beech_y-1_:site | -1 | 42 | 0.003 | 0.997 |
| 5 | log(CND) ~ beech+ site + beech:site | 0 | 39 | 0.350 | 0.707 |
| 6 | log(CND) ~ beech_y+1_ + site + beech_y+1_:site | +1 | 36 | 0.048 | 0.953 |
| 7 | log(CND) ~ beech_y+2_ + site + beech_y+2_:site | +2 | 33 | 0.398 | 0.676 |
| 8 | log(CND) ~ beech_y+3_ + site + beech_y+3_:site | +3 | 30 | 0.354 | 0.706 |
| 9 | log(CND) ~ beech_y+4_ + site + beech_y+4_:site | +4 | 27 | 0.347 | 0.711 |

Table S19. The log10-transformed CND was modeled as a linear model of the beech mast score with different time lags and site. Shown are the time lag (Lag), sample size (N), F-statistic of the model (F), p-value of the model (p), and the multiple r^2^ value of the model (expressed as a percent).

| **Model No.** | **Model Structure** | **Lag** | **N** | **F** | **p** | **r^2^ (%)** |
| --- | --- | --- | --- | --- | --- | --- |
| 10 | log(CND) ~ beech_y-4_ + site | -4 | 45 | 4.723 | 0.006 | 25.7 |
| 11 | log(CND) ~ beech_y-3_ + site | -3 | 45 | 6.222 | 0.001 | 31.3 |
| 12 | log(CND) ~ beech_y-2_ + site | -2 | 45 | 15.770 | < 0.001 | 53.6 |
| 13 | log(CND) ~ beech_y-1_ + site | -1 | 42 | 6.322 | 0.001 | 33.3 |
| 14 | log(CND) ~ beech + site | 0 | 39 | 5.722 | 0.003 | 32.9 |
| 15 | log(CND) ~ beech_y+1_ + site | 1 | 36 | 3.950 | 0.017 | 27.0 |
| 16 | log(CND) ~ beech_y+2_ + site | 2 | 33 | 3.878 | 0.019 | 28.6 |
| 17 | log(CND) ~ beech_y+3_ + site | 3 | 30 | 4.771 | 0.009 | 35.5 |
| 18 | log(CND) ~ beech_y+4_ + site | 4 | 27 | 4.263 | 0.016 | 35.7 |

Table S20. The log10-transformed CND is modeled as a linear function of (A) the lagged beech mast score and (B) site. Shown are the time lag (Lag), sample size (N), the F-statistic (F), p-value (p), and multiple r^2^ (expressed as a %).

| **Model No.** | **(A) Model Structure** | **Lag** | **N** | **F** | **p** | **r^2^ (%)** |
| --- | --- | --- | --- | --- | --- | --- |
| 19 | log(CND) ~ beech_y-4_ | -4 | 45 | 0.032 | 0.859 | 0.0 |
| 20 | log(CND) ~ beech_y-3_ | -3 | 45 | 2.588 | 0.115 | 5.7 |
| 21 | log(CND) ~ beech_y-2_ | -2 | 45 | 16.690 | < 0.001 | 28.0 |
| 22 | log(CND) ~ beech_y-1_ | -1 | 42 | 2.763 | 0.104 | 6.5 |
| 23 | log(CND) ~ beech | 0 | 39 | 3.047 | 0.089 | 7.6 |
| 24 | log(CND) ~ beech_y+1_ | 1 | 36 | 0.285 | 0.597 | 0.8 |
| 25 | log(CND) ~ beech_y+2_ | 2 | 33 | 1.777 | 0.192 | 5.4 |
| 26 | log(CND) ~ beech_y+3_ | 3 | 30 | 5.311 | 0.029 | 15.9 |
| 27 | log(CND) ~ beech_y+4_ | 4 | 27 | 4.044 | 0.055 | 13.9 |
| **Model No.** | **(B)** **Model Structure** | **Lag** | **N** | **F** | **p** | **r^2^ (%)** |
| 28 | log(CND) ~ site | -4 | 45 | 7.229 | 0.002 | 25.6 |
| 29 | log(CND) ~ site | -3 | 45 | 7.229 | 0.002 | 25.6 |
| 30 | log(CND) ~ site | -2 | 45 | 7.229 | 0.002 | 25.6 |
| 31 | log(CND) ~ site | -1 | 42 | 7.151 | 0.002 | 26.8 |
| 32 | log(CND) ~ site | 0 | 39 | 6.096 | 0.005 | 25.3 |
| 33 | log(CND) ~ site | 1 | 36 | 5.856 | 0.007 | 26.2 |
| 34 | log(CND) ~ site | 2 | 33 | 4.534 | 0.019 | 23.2 |
| 35 | log(CND) ~ site | 3 | 30 | 3.282 | 0.053 | 19.6 |
| 36 | log(CND) ~ site | 4 | 27 | 3.347 | 0.052 | 21.8 |

Table S21. Variance partitioning to determine how much variation in the CND could be attributed to the time lag between nymphs and beech mast score. Shown are the r^2^ values for the full models (models 10 to 18 models in Table S19) and the models containing site only (models 28 to 36 in Table S20). The difference between these two r^2^ values (Δ r^2^) indicates the variation that is caused by the time lag between nymphs and beech mast score.

| **Model comparison** | **Full model** | **Site model** | **Δ r^2^ (%)** |
| --- | --- | --- | --- |
| 10 vs 28 | 25.7 | 25.6 | 0.1 |
| 11 vs 29 | 31.3 | 25.6 | 5.7 |
| 12 vs 30 | 53.6 | 25.6 | 28.0 |
| 13 vs 31 | 33.3 | 26.8 | 6.5 |
| 14 vs 32 | 32.9 | 25.3 | 7.6 |
| 15 vs 33 | 27.0 | 26.2 | 0.8 |
| 16 vs 34 | 28.6 | 23.2 | 5.4 |
| 17 vs 35 | 35.5 | 19.6 | 15.9 |
| 18 vs 36 | 35.7 | 21.8 | 13.9 |

Table S22. The parameter estimates of the best model in Table S19 are shown. The best model (model 12 in Table S19) had the expected two-year time lag between high nymph abundance (CND) and high beech mast score (beech_y-2_). Shown are the parameter types, parameter names, parameter estimates on the logit scale, standard errors (s.e.), t-statistics (t), and p-values (p).

| **Type** | **Name** | **Estimates** | **s.e.** | **t** | **p** |
| --- | --- | --- | --- | --- | --- |
| Intercept | Low site | 4.136 | 0.057 | 72.164 | < 0.001 |
| Contrast 1 | Medium site | -0.083 | 0.058 | -1.418 | 0.164 |
| Contrast 2 | High site | -0.271 | 0.058 | -4.640 | < 0.001 |
| Slope 1 | Beech_y-2_ | 0.070 | 0.014 | 4.969 | < 0.001 |

# SECTION 10 – Climate change over the 15-year study period

**Methods:** We tested for climate change in the mean annual temperature over the 15-year study period for the field-collected data at each of the four elevation sites (low site, medium site, high site, top site) and for the Climap-net data for each of the two weather stations (Neuchâtel at 485 m ASL and Chaumont at 1136 m ASL) that are close to our study location. The mean annual temperature (n = 15) was modelled as a simple linear regression of the covariate year (rescaled as 1, 2, 3, … 15). We then used Pearson’s correlation test to determine whether the mean annual temperature was correlated between the field-collected data and the Climap-net data. The same approach was used to analyze the mean annual relative humidity, and the mean annual saturation deficit.

**Results – Climate change in field-collected variables and Climap-net variables:** The mean annual field-collected temperature decreased over the 15-year study period at the low elevation (F_1, 13_ = 0.057, p = 0.816), at the medium elevation (F_1, 13_ = 0.124, p = 0.731), at the high elevation (F_1, 13_ = 0.068, p = 0.799), and at the top elevation (F_1, 13_ = 0.007, p = 0.935), but these changes were not significant (Figure S14; Table S23). The mean annual field-collected relative humidity increased over the 15-year study period at the low elevation (F_1, 13_ = 0.677, p = 0.425), at the medium elevation (F_1, 13_ = 0.698, p = 0.419), at the high elevation (F_1, 13_ = 1.024, p = 0.330), and at the top elevation (F_1, 13_ = 0.116, p = 0.738), but these changes were not significant (Figure S15; Table S23). The mean annual field-collected saturation deficit decreased over the 15-year study period at the low elevation (F_1, 13_ = 0.455, p = 0.512), at the medium elevation (F_1, 13_ = 1.285, p = 0.277), at the high elevation (F_1, 13_ = 1.626, p = 0.225), and at the top elevation (F_1, 13_ = 0.462, p = 0.509) but these changes were not significant (Figure S16; Table S23). These analyses suggest that no significant climate change occurred with respect to any of the field-collected climate variables over the 15-year study period.

The mean annual Climap-net temperature increased significantly over the 15-year study period at the Neuchâtel weather station (F_1, 13_ =8.332, p = 0.013) and at the Chaumont weather station (F_1, 13_ = 7.540, p = 0.017) (Figure S17; Table S24). The mean annual Climap-net relative humidity increased over the 15-year study period at the Neuchâtel weather station (F_1, 13_ = 0.017, p = 0.898) and decreased at the Chaumont weather station (F_1, 13_ = 0.875, p = 0.367), but these changes were not significant (Figure S18; Table S24). The mean annual Climap-net saturation deficit increased over the 15-year study period at the Neuchâtel weather station (F_1, 13_ = 2.942, p = 0.110) and at the Chaumont weather station (F_1, 13_ = 6.225, p = 0.027), but these changes were only significant at the Chaumont weather station (Figure S19; Table S24). The mean annual Climap-net precipitation decreased over the 15-year study period at the Neuchâtel weather station (F_1, 13_ =0.949, p = 0.348) and at the Chaumont weather station (F_1, 13_ = 2.886, p = 0.113), but these changes were not significant (Figure S20; Table S24). These analyses suggest that significant climate change occurred with respect to temperature and saturation deficit from the Climap-net data over the 15-year study period.

**Results – Correlation between the field-collected and Climap-net annual temperature:** Across all four elevation sites, the mean annual temperature was weakly correlated between the field-collected data and the Neuchâtel weather station data (Pearson’s r = 0.318, n = 60, p = 0.013; Table S25). Taking each elevation site independently, the mean annual temperature was not correlated between the field-collected data and the Neuchâtel weather station data (Table S25).

Across all four elevation sites, the mean annual temperature was weakly correlated between the field-collected data and the Chaumont weather station data (Pearson’s r = 0.306, n = 60, p = 0.017; Table S25). Taking each elevation site independently, the mean annual temperature was not correlated between the field-collected data and the Chaumont weather station (Table S25). These analyses suggest that the mean annual temperature differed between the field-collected data and the Climap-net data over the 15-year study at Chaumont Mountain.

**Results – Correlation between the field-collected and Climap-net annual relative humidity:**

Across all four elevation sites, the mean annual relative humidity was weakly correlated between the field-collected data and the Neuchâtel weather station data (Pearson’s r = 0.367, n = 60, p = 0.004; Table S26). Taking each elevation site independently, the mean annual relative humidity was not correlated between the field-collected data and the Neuchâtel weather station data (Table S26).

Across all four elevation sites, the mean annual relative humidity was not correlated between the field-collected data and the Chaumont weather station data (Pearson’s r = 0.005, n = 60, p = 0.971; Table S26). Taking each elevation site independently, the mean annual relative humidity was not correlated between the field-collected data and the Chaumont weather station data (Table S26). These analyses suggest that the mean annual relative humidity differed between the field-collected data and the Climap-net data over the 15-year study at Chaumont Mountain.

**Results – Correlation between the field-collected and Climap-net annual saturation deficit:**

Across all four elevation sites, the mean annual saturation deficit was weakly correlated between the field-collected data and the Neuchâtel weather station data (Pearson’s r = 0.305, n = 60, p = 0.018; Table S27). Taking each elevation site independently, the mean annual saturation deficit was not correlated between the field-collected data and the Neuchâtel weather station data (Table S27).

Across all four elevation sites, the mean annual saturation deficit was not correlated between the field-collected data and the Chaumont weather station data (Pearson’s r = 0.162, n = 60, p = 0.218; Table S27). Taking each elevation site independently, the mean annual saturation deficit was not correlated between the field-collected data and the Chaumont weather station data (Table S27). These analyses suggest that the mean annual relative humidity differed between the field-collected data and the Climap-net data over the 15-year study at Chaumont Mountain.

Table S23. The simple linear regression models of each climate variable versus the covariate year are shown for the field-collected data. Shown are the parameter estimates, standard errors (s.e.), t-statistics (t), degrees of freedom (df), p-values (p), and the adjusted r^2^ values of the model (expressed as a percent).

| **Climate variable** | **Site** | **Parameters** | **Estimates** | **s.e.** | **t** | **df** | **p** | **r^2^(%)** |
| --- | --- | --- | --- | --- | --- | --- | --- | --- |
| Temperature | Low | (Intercept) | 15.553 | 0.673 | 23.097 | 13 | < 0.001 | 0.0 |
|  |  | Year | -0.018 | 0.074 | -0.238 | 1 | 0.816 |  |
| Temperature | Medium | (Intercept) | 14.574 | 0.813 | 17.929 | 13 | < 0.001 | 0.0 |
|  |  | Year | -0.031 | 0.089 | -0.351 | 1 | 0.731 |  |
| Temperature | High | (Intercept) | 13.418 | 0.827 | 16.230 | 13 | < 0.001 | 0.0 |
|  |  | Year | -0.024 | 0.091 | -0.260 | 1 | 0.799 |  |
| Temperature | Top | (Intercept) | 11.438 | 0.843 | 13.571 | 13 | < 0.001 | 0.0 |
|  |  | Year | -0.008 | 0.093 | -0.084 | 1 | 0.935 |  |
| Relative humidity | Low | (Intercept) | 59.385 | 3.473 | 17.101 | 13 | < 0.001 | 0.0 |
|  |  | Year | 0.314 | 0.382 | 0.823 | 1 | 0.425 |  |
| Relative humidity | Medium | (Intercept) | 60.904 | 3.755 | 16.219 | 13 | < 0.001 | 0.0 |
|  |  | Year | 0.345 | 0.413 | 0.835 | 1 | 0.419 |  |
| Relative humidity | High | (Intercept) | 63.598 | 3.199 | 19.880 | 13 | < 0.001 | 0.0 |
|  |  | Year | 0.356 | 0.352 | 1.012 | 1 | 0.330 |  |
| Relative humidity | Top | (Intercept) | 69.385 | 3.317 | 20.918 | 13 | < 0.001 | 0.0 |
|  |  | Year | 0.125 | 0.365 | 0.341 | 1 | 0.738 |  |
| Saturation deficit | Low | (Intercept) | 6.683 | 0.722 | 9.262 | 13 | < 0.001 | 0.0 |
|  |  | Year | -0.054 | 0.079 | -0.674 | 1 | 0.512 |  |
| Saturation deficit | Medium | (Intercept) | 6.174 | 0.704 | 8.765 | 13 | < 0.001 | 2.0 |
|  |  | Year | -0.088 | 0.077 | -1.134 | 1 | 0.277 |  |
| Saturation deficit | High | (Intercept) | 5.184 | 0.560 | 9.264 | 13 | < 0.001 | 4.3 |
|  |  | Year | -0.078 | 0.062 | -1.275 | 1 | 0.225 |  |
| Saturation deficit | Top | (Intercept) | 3.793 | 0.488 | 7.775 | 13 | < 0.001 | 0.0 |
|  |  | Year | -0.036 | 0.054 | -0.680 | 1 | 0.509 |  |

Table S24. The simple linear regression models of each climate variable versus the covariate year are shown for the field-collected data. Shown are the parameter estimates, standard errors (s.e.), t-statistics (t), degrees of freedom (df), p-values (p), and the adjusted r^2^ values of the model (expressed as a percent).

| **Climate variable** | **Station** | **Parameters** | **Estimates** | **s.e.** | **t** | **df** | **p** | **r^2^(%)** |
| --- | --- | --- | --- | --- | --- | --- | --- | --- |
| Temperature | Neuchâtel | (Intercept) | 10.118 | 0.260 | 38.865 | 13 | < 0.001 | 34.4 |
|  |  | Year | 0.083 | 0.029 | 2.886 | 1 | 0.013 |  |
| Temperature | Chaumont | (Intercept) | 6.177 | 0.315 | 19.607 | 13 | < 0.001 | 31.8 |
|  |  | Year | 0.095 | 0.035 | 2.746 | 1 | 0.017 |  |
| Relative humidity | Neuchâtel | (Intercept) | 73.021 | 0.815 | 89.625 | 13 | < 0.001 | 0.0 |
|  |  | Year | 0.012 | 0.090 | 0.131 | 1 | 0.898 |  |
| Relative humidity | Chaumont | (Intercept) | 78.126 | 0.796 | 98.185 | 13 | < 0.001 | 0.0 |
|  |  | Year | -0.082 | 0.088 | -0.936 | 1 | 0.367 |  |
| Saturation deficit | Neuchâtel | (Intercept) | 2.962 | 0.146 | 20.244 | 13 | < 0.001 | 12.2 |
|  |  | Year | 0.028 | 0.016 | 1.715 | 1 | 0.110 |  |
| Saturation deficit | Chaumont | (Intercept) | 1.887 | 0.103 | 18.263 | 13 | < 0.001 | 27.2 |
|  |  | Year | 0.028 | 0.011 | 2.495 | 1 | 0.027 |  |
| Precipitation | Neuchâtel | (Intercept) | 2.823 | 0.293 | 9.626 | 13 | < 0.001 | 0.0 |
|  |  | Year | -0.031 | 0.032 | -0.974 | 1 | 0.348 |  |
| Precipitation | Chaumont | (Intercept) | 3.674 | 0.275 | 13.371 | 13 | < 0.001 | 11.9 |
|  |  | Year | -0.051 | 0.030 | -1.699 | 1 | 0.113 |  |


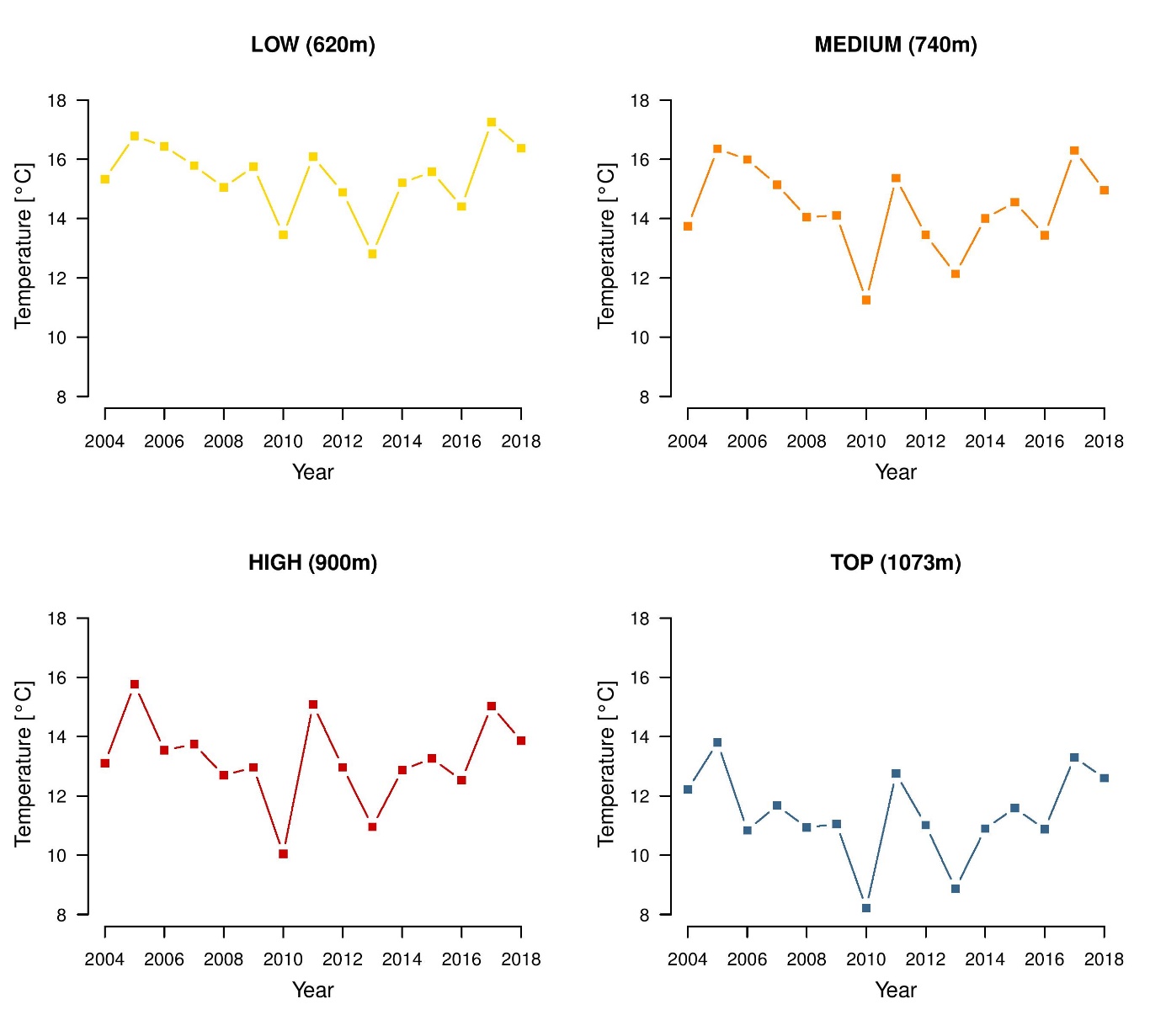
Figure S14. The change in the mean annual field-collected temperature over the 15-year study period is shown for each of the four elevations. Temperature decreased over time at each elevation but these changes were not significant.


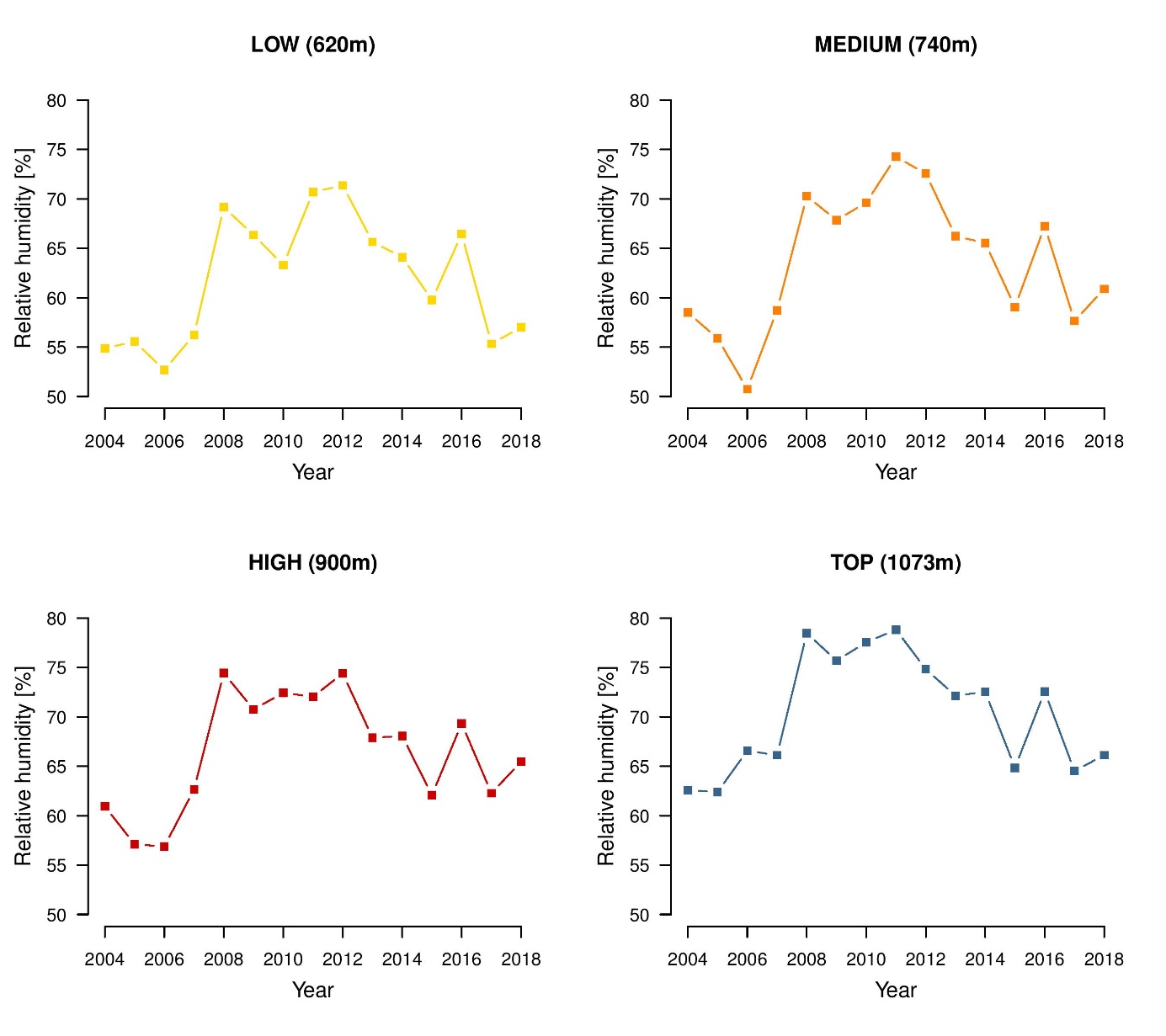
Figure S15. The change in the mean annual field-collected relative humidity over the 15-year study period is shown for each of the four elevations. Relative humidity increased over time at each elevation but these changes were not significant.


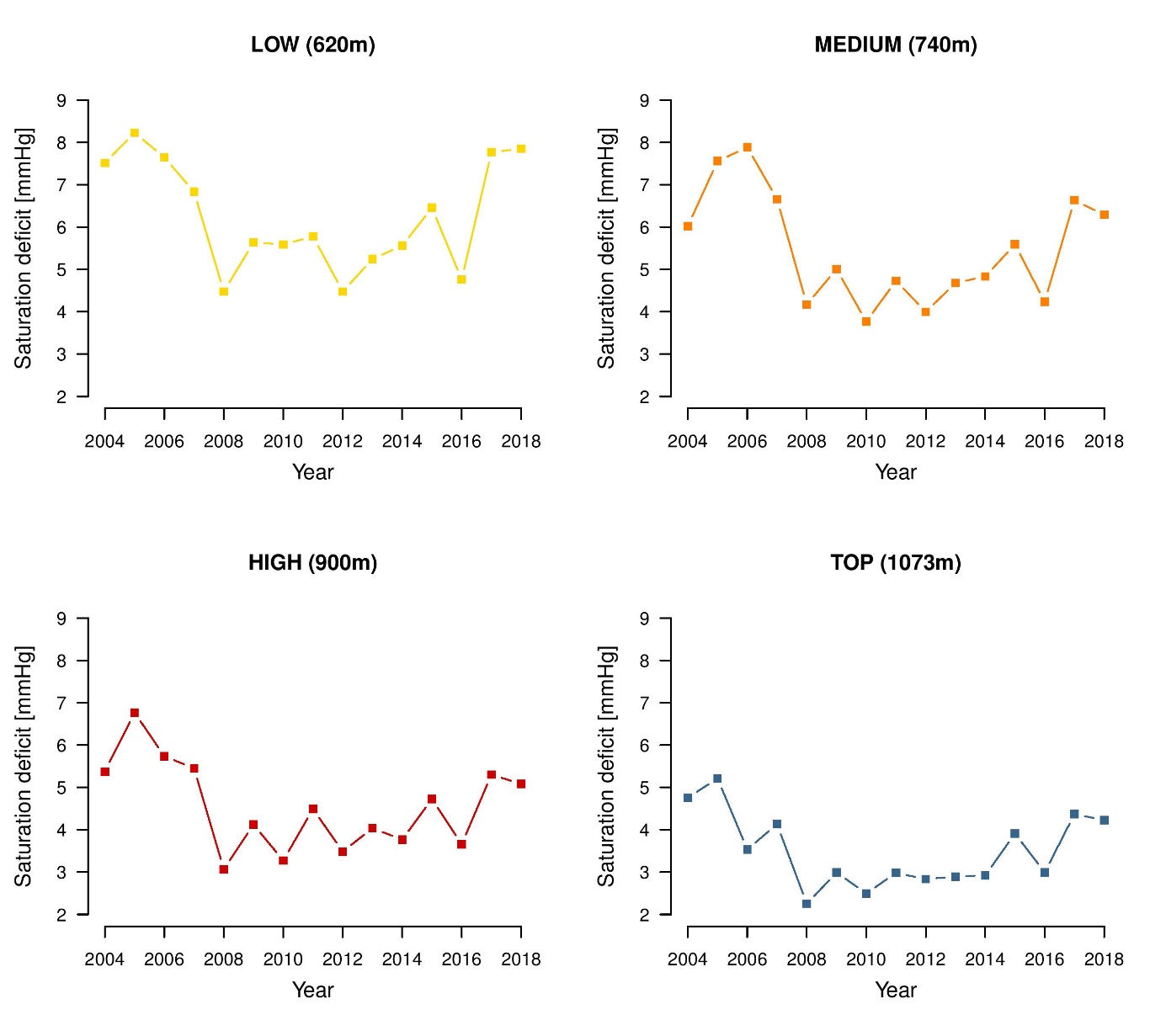
Figure S16. The change in the mean annual field-collected saturation deficit over the 15-year study period is shown for each of the four elevations. Saturation deficit decreased over time at each elevation but these changes were not significant.


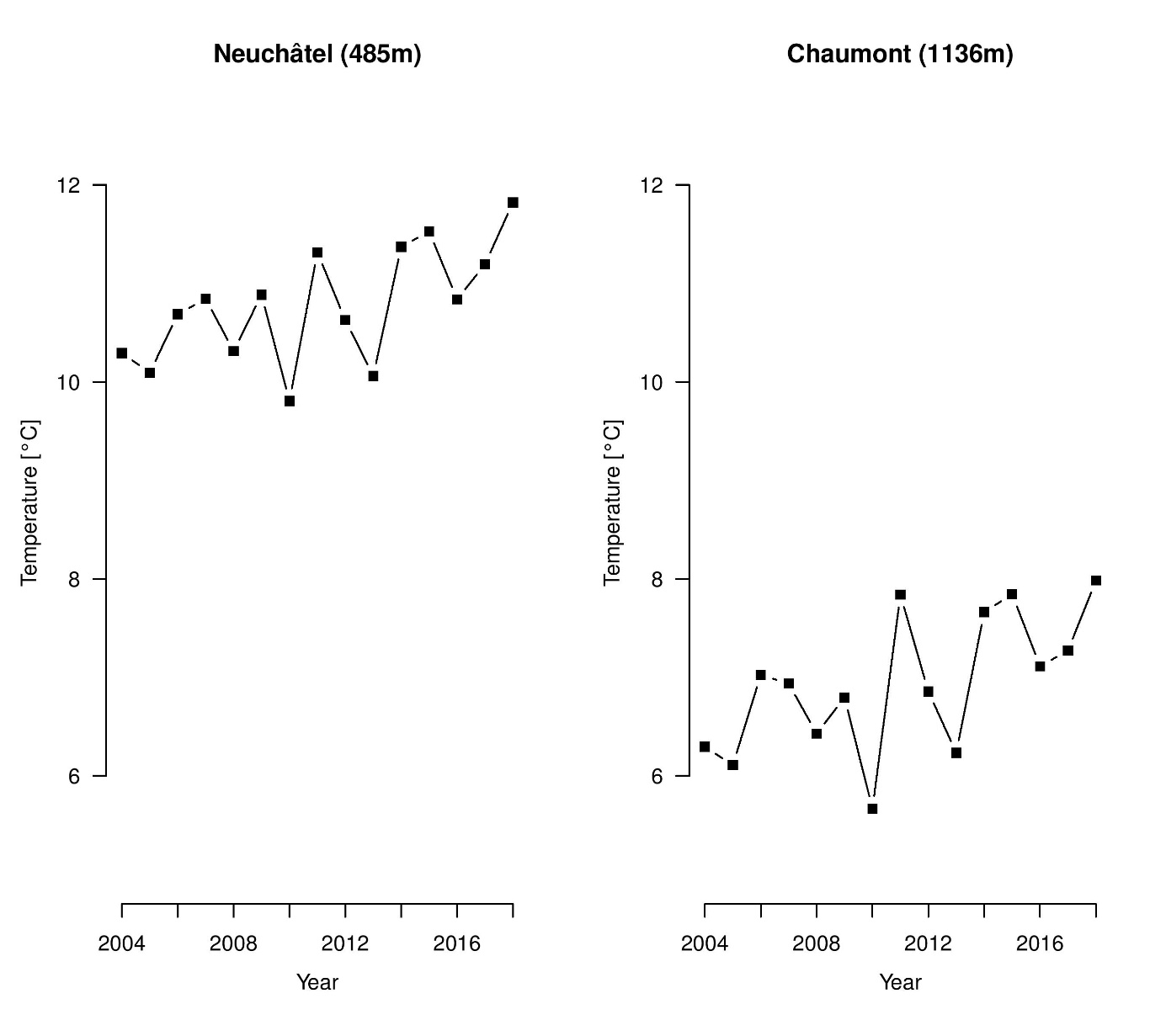
Figure S17. The change in the mean annual Climap-net temperature over the 15-year study period is shown for each of the two weather stations. Temperature increased significantly over time at each weather station.


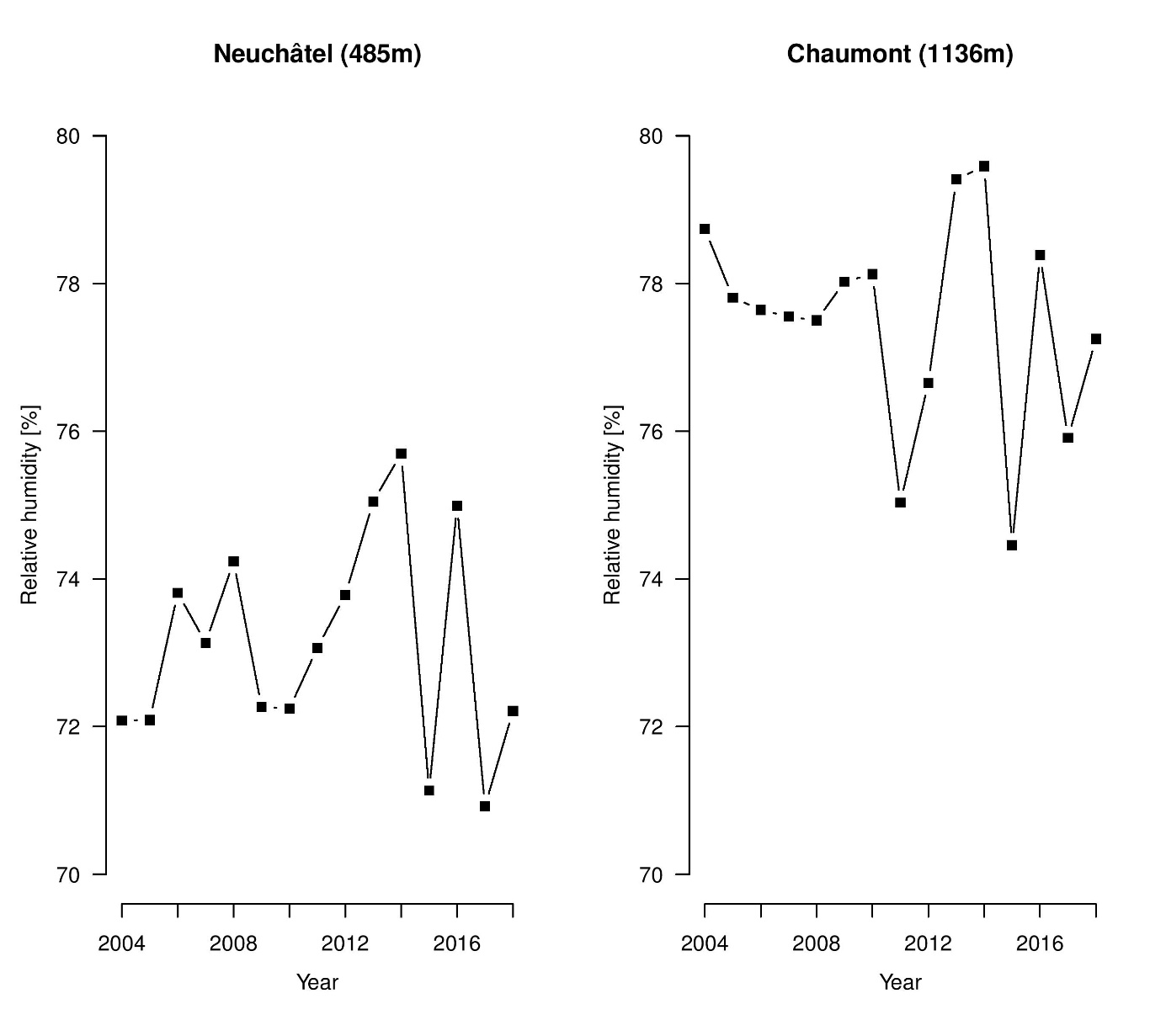
Figure S18. The change in the mean annual Climap-net relative humidity over the 15-year study period is shown for each of the two weather stations. Relative humidity increased over time at the Neuchatel weather station but decreased over time at the Chaumont weather station but this change was not significant.


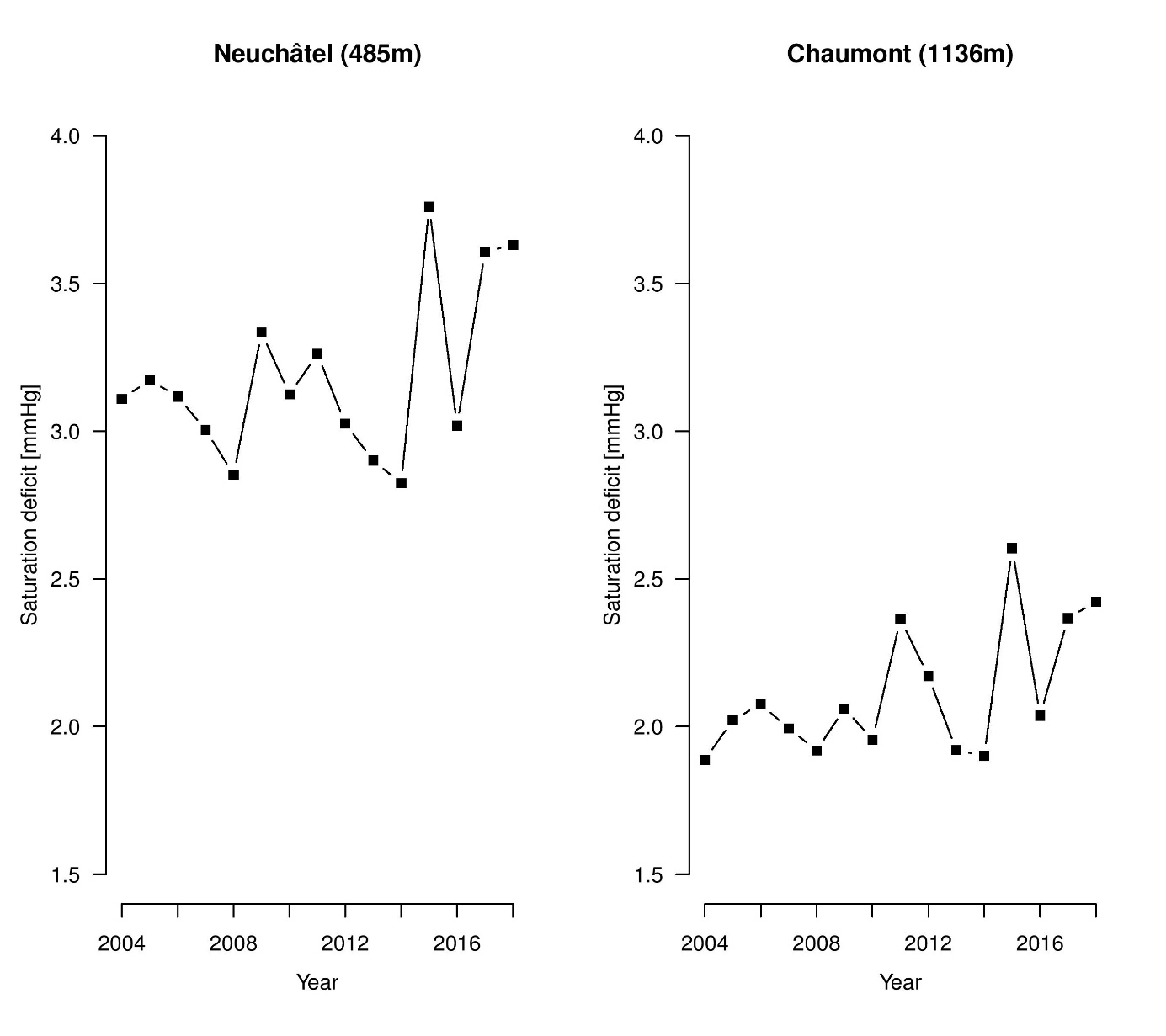
Figure S19. The change in the mean annual Climap-net saturation deficit over the 15-year study period is shown for each of the two weather stations. Saturation deficit increased over time, but this change was only significant for the Chaumont weather station.


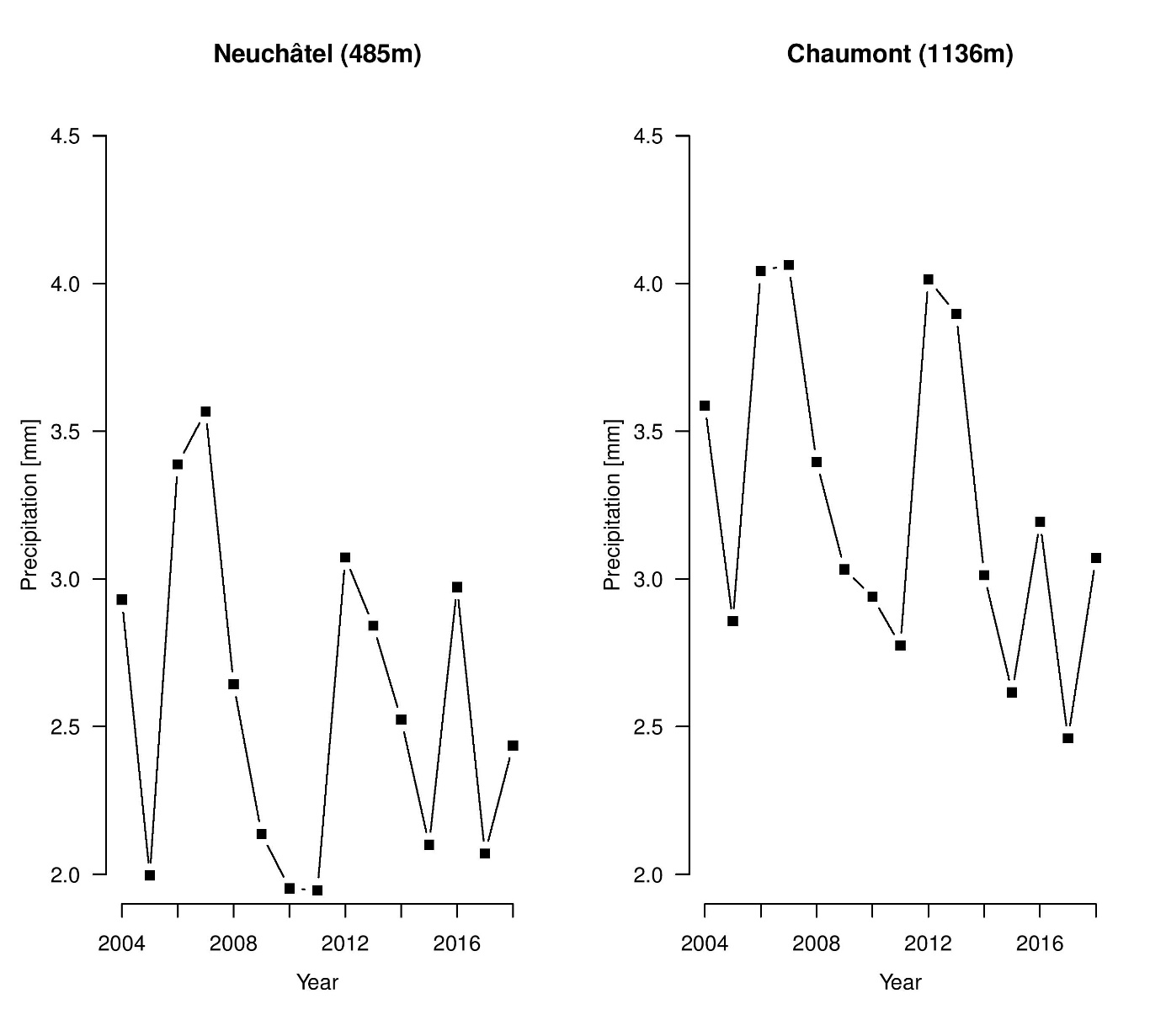
Figure S20. The change in the mean annual Climap-net precipitation over the 15-year study period is shown for each of the two weather stations. Precipitation decreased over time, but this change was not significant at either weather station.

Table S25. The correlation in the annual mean temperature between the field-collected data and the Climap-net data is shown for each of the eight combinations of the four elevation sites and the two weather stations. The four sites were located on the south side of Chaumont Mountain. The two weather stations in Neuchâtel and Chaumont were located at 485 m ASL and 1136 m ASL, respectively. The mean annual temperature for the field-collected data and the two weather stations were measured at 60 cm above ground and 200 cm above ground, respectively. Shown are Pearson’s correlation coefficient (r), the sample size (n), and the statistical significance of the correlation (p).

|  |  | **Neuchâtel^b^** |  |  | **Chaumont^c^** |  |
| --- | --- | --- | --- | --- | --- | --- |
| **Site** | **r** | **n** | **p** | **r** | **n** | **p** |
| Low | 0.520 | 15 | 0.047 | 0.472 | 15 | 0.075 |
| Medium | 0.464 | 15 | 0.081 | 0.464 | 15 | 0.081 |
| High | 0.451 | 15 | 0.092 | 0.454 | 15 | 0.089 |
| Top | 0.465 | 15 | 0.080 | 0.436 | 15 | 0.104 |
| All^a^ | 0.318 | 60 | 0.013 | 0.306 | 60 | 0.017 |

^a^ The daily mean temperatures were positively weakly correlated between the field-collected data and the Climap-net data from both weather stations.

Table S26. The correlation in the annual mean relative humidity between the field-collected data and the Climap-net data is shown for each of the eight combinations of the four elevation sites and the two weather stations. The four sites were located on the south side of Chaumont Mountain. The two weather stations in Neuchâtel and Chaumont were located at 485 m ASL and 1136 m ASL, respectively. The mean annual relative humidity for the field-collected data and the two weather stations were measured at 60 cm above ground and 200 cm above ground, respectively. Shown are Pearson’s correlation coefficient (r), the sample size (n), and the statistical significance of the correlation (p).

|  |  | **Neuchâtel^b^** |  |  | **Chaumont^c^** |  |
| --- | --- | --- | --- | --- | --- | --- |
| **Site** | **r** | **n** | **p** | **r** | **n** | **p** |
| Low | 0.475 | 15 | 0.074 | 0.037 | 15 | 0.895 |
| Medium | 0.359 | 15 | 0.189 | 0.017 | 15 | 0.953 |
| High | 0.375 | 15 | 0.169 | 0.026 | 15 | 0.928 |
| Top | 0.461 | 15 | 0.084 | 0.057 | 15 | 0.841 |
| All^a^ | 0.367 | 60 | 0.004 | 0.005 | 60 | 0.971 |

^a^ The daily mean relative humidity were positively weakly correlated between the field-collected data and the Climap-net data only from the Neuchâtel weather station.

Table S27. The correlation in the annual mean saturation deficit between the field-collected data and the Climap-net data is shown for each of the eight combinations of the four elevation sites and the two weather stations. The four sites were located on the south side of Chaumont Mountain. The two weather stations in Neuchâtel and Chaumont were located at 485 m ASL and 1136 m ASL, respectively. Shown are Pearson’s correlation coefficient (r), the sample size (n), and the statistical significance of the correlation (p).

|  |  | **Neuchâtel^b^** |  |  | **Chaumont^c^** |  |
| --- | --- | --- | --- | --- | --- | --- |
| **Site** | **r** | **n** | **p** | **r** | **n** | **p** |
| Low | 0.519 | 15 | 0.047 | 0.273 | 15 | 0.326 |
| Medium | 0.339 | 15 | 0.217 | 0.175 | 15 | 0.532 |
| High | 0.371 | 15 | 0.173 | 0.204 | 15 | 0.466 |
| Top | 0.459 | 15 | 0.086 | 0.242 | 15 | 0.384 |
| All^a^ | 0.305 | 60 | 0.018 | 0.162 | 60 | 0.218 |

^a^ The daily mean saturation deficit were positively weakly correlated between the field-collected data and the Climap-net data only from the Neuchâtel weather station.

# SECTION 11 – Analysis of CND using Generalized Linear Models with Negative Binomial Errors

The cumulative nymphal density (CND) estimates the cumulative number of questing nymphs that were removed from each elevation site over the year. Each estimate of the CND is based on 12 sampling dates, and each sampling date is a mean of the tick counts that were obtained from 5 or 6 drags. In the main manuscript, we analyzed the log10-transformed CND (or CAD) using linear models with normal errors. In Section 7 of the Additional file 1, we showed that the residuals of these linear models met the assumptions of normality and equal variances. Here we present an alternative approach where we analyze the count data using generalized linear models (GLMs) with a Poisson error distribution or a negative binomial error distribution.

**GLM with Poisson errors:** To analyze the CND as a GLM with Poisson errors, we rounded each estimate of the CND to the nearest integer. We modelled the CND as a GLM with Poisson errors with the following fixed factors: elevation site, year, beech, and the field-collected relative humidity. A major problem with this approach was that the ratio of the residual deviance to the residual degrees of freedom (58778/36 = 1632.722) was highly overdispersed. In R, one can use the quasipoisson distribution to deal with overdispersed count data. However, the disadvantage of using the quasipoisson distribution to deal with overdispersion is that you can no longer use model selection because the model does not estimate an AIC value. For this reason, we decided to use a GLM with negative binomial errors to analyze our data.

**GLM with negative binomial errors:** To analyze the CND as a GLM with Poisson errors, we rounded each estimate of the CND to the nearest integer. For the GLM with negative binomial errors of the CND, we used the same AIC-based model selection approach that we used for the linear model with normal errors of the log10-transformed CND. We present the model selection table for the GLM with negative binomial errors of the CND in Table S28. For ease of comparison, we present the model selection table of the linear model of the log10-transformed CND in Table S29. For both approaches, the best model included the main effects of elevation site, year, beech masting index, and the field-collected relative humidity. This comparison shows that the two approaches returned the same model selection results. For the GLM with negative binomial errors of the CND, we present the parameter estimates of the best model (model 1 in Table S28) in Table S30. For the linear model of the log10-transformed CND, we present the parameter estimates of the best model (model 1 in Table S29) in Table S31. The pattern of statistical significance is almost identical between the two tables. All the parameter estimates have the same sign. The magnitudes of the parameter estimates are not the same because the CNDs were log10-transformed for the linear model but not for the GLM. This comparison shows that these two approaches give the same results.

Table S28. Model selection results are shown for the negative binomial GLM of the cumulative nymph density response variable. The explanatory variables were site, year, tree masting variables obtained from MASTREE, and the climate variables obtained from the Climap-net database and collected from the field. The models are ranked according to their Akaike Information Criterion (AIC). Of the 52 models in the set, only the 6 top models are shown for which the cumulative support (Weight 2) is 95%. Shown for each model are the model rank (Rank), model structure (see below for explanation of explanatory variables), model degrees of freedom (Df), log-likelihood (logLik), Akaike information criterion (AIC), difference in the AIC value from the top model (ΔAIC), model weight (Weight1), cumulative weight (Weight2), and adjusted r-squared value (r^2^). Additional file 1: Section 6 shows the results from the full model selection. The acronyms for the explanatory variables are as follows: S = site, Y = year, B = beech mast score, S:Y = interaction between site and year, RH2 = relative humidity from the field data, SD2 = saturation deficit from the field data, T2 = temperature from the field data, RH2_y-1_ = relative humidity from the field data in year y-1, and SD2_y-1_ = saturation deficit from the field data in year y-1.

| **Rank** | **Model structure** | **Df** | **logLik** | **AIC** | **ΔAIC** | **Weight1** | **Weight2** | **r^2^** |
| --- | --- | --- | --- | --- | --- | --- | --- | --- |
| 1 | CND ~ S+Y+B+RH2 | 7 | -409.4 | 836.2 | 0.0 | 75.0 | 75.0 | NA |
| 2 | CND ~ S+Y+B+RH2+S:Y | 9 | -408.4 | 840.4 | 4.2 | 9.0 | 84.0 | NA |
| 3 | CND ~ S+Y+B+SD2 | 7 | -412.2 | 841.6 | 5.5 | 5.0 | 89.0 | NA |
| 4 | CND ~ S+Y+B+T2 | 7 | -412.3 | 841.9 | 5.8 | 4.0 | 93.0 | NA |
| 5 | CND ~ S+Y+B+RH2_y-1_ | 7 | -413.3 | 843.9 | 7.7 | 2.0 | 95.0 | NA |
| 6 | CND ~ S+Y+B+SD2_y-1_ | 7 | -413.4 | 844.1 | 7.9 | 1.0 | 96.0 | NA |

Table S29. Model selection results are shown for the linear models with normal errors of the log10-transformed CND response variable. The explanatory variables were site, year, tree masting variables obtained from MASTREE, and the climate variables obtained from the Climap-net database and collected from the field. The models are ranked according to their Akaike Information Criterion (AIC). Of the 52 models in the set, only the 6 top models are shown for which the cumulative support (Weight 2) is 95%. Shown for each model are the model rank (Rank), model structure (see below for explanation of explanatory variables), model degrees of freedom (Df), log-likelihood (logLik), Akaike information criterion (AIC), difference in the AIC value from the top model (ΔAIC), model weight (Weight1), cumulative weight (Weight2), and adjusted r-squared value (r^2^). Additional file 1: Section 6 shows the results from the full model selection. The acronyms for the explanatory variables are as follows: S = site, Y = year, B = beech mast score, S:Y = interaction between site and year, RH2 = relative humidity from the field data, SD2 = saturation deficit from the field data, T2 = temperature from the field data, RH2_y-1_ = relative humidity from the field data in year y-1, and SD2_y-1_ = saturation deficit from the field data in year y-1.

| **Rank** | **Model structure** | **Df** | **logLik** | **AIC** | **ΔAIC** | **Weight1** | **Weight2** | **r^2^** |
| --- | --- | --- | --- | --- | --- | --- | --- | --- |
| 1 | CND ~ S+Y+B+RH2 | 7 | 33.0 | -48.7 | 0.0 | 76.0 | 76.0 | 73.2 |
| 2 | CND ~ S+Y+B+RH2+S:Y | 9 | 33.6 | -43.6 | 5.2 | 6.0 | 82.0 | 72.4 |
| 3 | CND ~ S+Y+B+SD2 | 7 | 30.4 | -43.5 | 5.3 | 5.0 | 87.0 | 69.7 |
| 4 | CND ~ S+Y+B+T2 | 7 | 30.4 | -43.4 | 5.3 | 5.0 | 92.0 | 69.6 |
| 5 | CND ~ S+Y+B+RH2_y-1_ | 7 | 29.4 | -41.4 | 7.3 | 2.0 | 94.0 | 68.1 |
| 6 | CND ~ S+Y+B+SD2_y-1_ | 7 | 29.1 | -40.8 | 7.9 | 1.0 | 95.0 | 67.7 |

Table S30. The parameter estimates from the top model in the model selection table from negative binomial GLM are shown. In this top model, the CND response variable was modelled as a function of elevation site, year, beech tree mast score 2 years prior, and the field-collected mean annual relative humidity in the same year. Shown are the parameter types, parameter names, parameter estimates on the log10-transformed scale, standard errors (s.e.), t-statistic (t), and p-values (p).

| **Type** | **Name** | **Estimate** | **s.e** | **t** | **p** |
| --- | --- | --- | --- | --- | --- |
| **Intercept** | **Low site** | **9.102** | **0.127** | **71.645** | **< 0.001** |
| Contrast 1 | Medium site | -0.136 | 0.097 | -1.403 | 0.161 |
| **Contrast 2** | **High site** | **-0.590** | **0.100** | **-5.878** | **< 0.001** |
| **Slope 1** | **Year** | **0.046** | **0.010** | **4.641** | **< 0.001** |
| **Slope 2** | **Beech tree mast score** | **0.159** | **0.023** | **7.053** | **< 0.001** |
| **Slope 3** | **Relative humidity (field-collected)** | **-0.170** | **0.045** | **-3.814** | **< 0.001** |

Table S31. The parameter estimates from the top model in the model selection table from linear models are shown. In this top model, the log10-transformed CND response variable was modelled as a function of elevation site, year, beech tree mast score 2 years prior, and the field-collected mean annual relative humidity in the same year. Shown are the parameter types, parameter names, parameter estimates on the log10-transformed scale, standard errors (s.e.), t-statistic (t), and p-values (p).

| **Type** | **Name** | **Estimate** | **s.e** | **t** | **p** |
| --- | --- | --- | --- | --- | --- |
| **Intercept** | **Low site** | **3.939** | **0.059** | **66.209** | **< 0.001** |
| Contrast 1 | Medium site | -0.053 | 0.045 | -1.162 | 0.253 |
| **Contrast 2** | **High site** | **-0.245** | **0.047** | **-5.217** | **< 0.001** |
| **Slope 1** | **Year** | **0.020** | **0.005** | **4.262** | **< 0.001** |
| **Slope 2** | **Beech tree mast score** | **0.067** | **0.011** | **6.378** | **< 0.001** |
| **Slope 3** | **Relative humidity (field-collected)** | **-0.074** | **0.021** | **-3.570** | **0.001** |

REFERENCES

1. BikeAttitude: BikeAttitude, The World of Freeride. [www.neuchbikepark.ch](file:///F:\0%20P&V\0000%20EDITS\000%20EDITS%20MSs\284%20AGUSTIN%20AKK\www.neuchbikepark.ch) (2003). Accessed October 28 2019.

2. Neuchâtel LVd: Fête de Chaumont. [www.neuchatelville.ch/fileadmin/sites/ne_ville/fichiers/presse/communiques_presse/imported/2011/Fete_20de_20Chaumont.pdf](file:///F:\0%20P&V\0000%20EDITS\000%20EDITS%20MSs\284%20AGUSTIN%20AKK\www.neuchatelville.ch\fileadmin\sites\ne_ville\fichiers\presse\communiques_presse\imported\2011\Fete_20de_20Chaumont.pdf) (2011). Accessed October 28 2019.

3. Tack W, Madder M, Baeten L, Vanhellemont M, Verheyen K. Shrub clearing adversely affects the abundance of Ixodes ricinus ticks. Exp Appl Acarol. 2013;60:411-20.
